# Associations of MAFLD subtypes and air pollutants with multi-system morbidity and all-cause mortality: A prospective cohort study

**DOI:** 10.1016/j.ecoenv.2025.117893

**Published:** 2025-02

**Authors:** Jingyi Zhang, Shanshan Ran, Shengtao Wei, Fei Tian, Lan Chen, Zijun Yang, Ge Chen, Hualiang Lin

**Affiliations:** Department of Epidemiology, School of Public Health, Sun Yat-sen University, Guangzhou 510080, China

**Keywords:** MAFLD, Ambient air pollution, Cirrhosis, ASCVD, CKD

## Abstract

**Background:**

Metabolic dysfunction-associated fatty liver disease (MAFLD) and air pollution are both significant health concerns. However, their combined effects on multi-system morbidity and all-cause mortality remain poorly understood.

**Methods:**

We analyzed data from 434,417 UK Biobank participants, categorizing them into four groups: non-MAFLD, MAFLD-diabetes, MAFLD-lean, and MAFLD-overweight/obesity. To evaluate the long-term effects of air pollution exposure, we used time-varying Cox proportional hazard models to assess four air pollutants: particulate matter with an aerodynamic diameter < 2.5 μm (PM_2.5_), PM_10_, nitrogen dioxide (NO_2_), and nitrogen oxides (NO_x_). We examined the associations between these air pollutants, MAFLD subtypes, and their joint impact on multi-system morbidity and all-cause mortality. Furthermore, we explored the additive and multiplicative interactions between air pollutants and MAFLD subtypes.

**Results:**

At baseline, 15,325 participants were classified as MAFLD-diabetes, 3341 as MAFLD-lean, and 140,934 as MAFLD-overweight/obesity. Among these groups, MAFLD-diabetes was most strongly associated with adverse outcomes compared to other subtypes. Air pollution exposure had a synergistic effect on cirrhosis risk across all MAFLD subtypes, with the most pronounced effects observed for PM_2.5_ [relative excess risk due to interaction (RERI): 2.10 (0.94, 3.26)] and NO_2_ [RERI:1.85 (0.67, 3.04)] in MAFLD-lean group. Positive additive and multiplicative interactions between air pollutants and MAFLD subtypes were also observed for coronary artery disease (CAD), with the exception of nitrogen oxide in the MAFLD-lean group. Additionally, only the MAFLD-diabetes demonstrated significant positive additive interactions with all four air pollutants in relation to chronic kidney disease (CKD).

**Conclusions:**

This study highlights the distinct impacts of MAFLD subtypes on multi-system morbidity and all-cause mortality, underscoring the critical need for targeted prevention and treatment strategies, particularly for individuals with MAFLD-diabetes. Our findings reveal significant additive and synergistic effects of air pollution exposure on the risks of cirrhosis, CAD, and CKD among MAFLD patients.

## Highlights

• MAFLD subtypes affect outcomes differently, highlighting the need for tailored care.
• Synergistic effects of air pollutants and MAFLD raise risks of cirrhosis, CAD, and CKD.
• Addressing environmental factors is key to managing public health risks of MAFLD.

## 1 Introduction

Metabolic dysfunction-associated fatty liver disease (MAFLD) is characterized by the coexistence of metabolic disorders and hepatic steatosis (Eslam et al., 2020). Affecting approximately 25 % of the global adult population, MAFLD represents a major public health challenge, particularly in the absence of effective pharmacotherapy (Younossi et al., 2016). While traditionally associated with intrahepatic complications, there is growing recognition of MAFLD’s impact on multi-system health, including metabolic, cardiovascular, and renal disease (Byrne and Targher, 2015, Crane et al., 2024, Kim et al., 2021, Liu et al., 2022b). MAFLD is a heterogeneous condition comprising distinct subtypes: MAFLD-diabetes, MAFLD-lean, and MAFLD-overweight/obesity (Huang et al., 2021). Each subtype carries varying levels of risk for cardiovascular outcomes and mortality, depending on the underlying metabolic dysfunctions (Chen et al., 2021, Chung et al., 2023). However, the comprehensive health effects of MAFLD subtypes remain insufficiently explored.

Ambient air pollution is a modifiable risk factor linked to a wide range of health conditions, including diabetes, cardiovascular, kidney, and digestive diseases (Collaborators, 2020). Emerging evidence also associates air pollution with increased MAFLD prevalence. Long term exposure to ambient PM and NO_2_ has been shown to heighten MAFLD risk by inducing oxidative stress and inflammation (Guo et al., 2022). These mechanisms can lead to conditions resembling non-alcoholic steatohepatitis (NASH), further resulting in insulin resistance, glucose intolerance, and hepatic fibrogenesis (Zheng et al., 2013). Moreover, air pollution might exacerbate the adverse effects of metabolic dysfunction, acting synergistically to worsen disease outcomes (Yang et al., 2019). Studies have also demonstrated that prolonged exposure to PM can accelerate liver fibrosis in individuals with MAFLD (Xing et al., 2023). However, much of the existing research is limited by cross-sectional study designs or a narrow focus on intrahepatic outcomes.

ASCVD and CKD are particularly relevant endpoints due to their shared pathophysiological mechanisms with MAFLD, including chronic inflammation, oxidative stress, and metabolic dysfunction, all of which might be further exacerbated by external environmental factors, such as air pollution (Ndumele et al., 2023). However, most existing studies have focused on the individual effects of air pollutants and MAFLD, often overlooking their potential interactions. Exploring the synergistic effects of MAFLD and air pollution is especially critical, as both factors share common biological pathways-such as oxidative stress, inflammation, and systemic metabolic dysregulation-that may collectively amplify the risk of multi-system diseases. Gaining a deeper understanding of these interactive effects could provide a strong scientific foundation to implement coordinated strategies for controlling both metabolic and environmental risk factors.

Given the varying clinical outcomes associated with different MAFLD subtypes, as well as the potential interaction between air pollution and MAFLD, this study aims to investigate the differential risks of multi-system diseases and all-cause mortality across MAFLD subgroups. Additionally, it examines the joint effects of MAFLD subtypes and long-term air pollution exposure on health outcomes, providing insights into their combined impact on systemic disease burden.

## 2 Methods

### 2.1 Study population

The UK Biobank is a large-scale cohort study comprising over 500,000 participants aged 37–73 years from the United Kingdom (UK). Conducted from 2006 to 2010, the baseline survey incorporated touchscreen questionnaires, interviews, physical measurements, and the collection of blood, urine, and saliva samples for laboratory analysis. Participants were subsequently followed up until October 31, 2022 for those in England, May 31, 2022 for those in Wales, and August 31, 2022 for those in Scotland (Sudlow et al., 2015). Ethical approval was obtained from the North West Multi-Centre Research Ethics Committee (Ref: 11/NW/03820), with all participants providing written informed consent.

### 2.2 Definitions of MAFLD and its types

Although ultrasonography is recognized as a first-line screening technique in clinical practice (Chalasani et al., 2012), however, these specific imaging and histological data were not broadly available in the UK Biobank. Consequently, the diagnosis of MAFLD was established based on international consensus (Eslam et al., 2020), utilizing the Fatty liver index (FLI) as a valid indicator of hepatic steatosis in the absence of imaging and histological data, defined as FLI ≥ 60 (Castera et al., 2019, Liu et al., 2022b). The FLI proves practical for screening the general population in epidemiologic studies, demonstrating a strong correlation with ultrasound diagnoses of MAFLD, supported by a specificity of 92.5 %, sensitivity of 96 %, and area under curve (AUC) of 0.92 for detecting fatty liver on ultrasound (Jones et al., 2022, Vamja et al., 2024). The FLI is calculated using four parameters: body mass index (BMI), waist circumference (WC), γ-glutamyltransferase (GGT), and serum triglyceride (TG), measured in the following units: BMI in kg/m^2^, WC in cm, GGT in U/L, and TG in mmol/L.

The diagnosis of MAFLD required confirmed hepatic steatosis alongside at least two of the following condition: type 2 diabetes mellitus (T2DM), overweight or obesity (BMI ≥25 kg/m^2^), or at least two metabolic dysregulations. T2DM was identified by glycated hemoglobin (HbA1c) ≥ 48 mmol/mol at baseline, a self-reported history, or any antihyperglycemic medications history. Metabolic dysregulations included blood pressure ≥ 130/85 mmHg or medication use, waist circumference ≥ 102/88 cm (male/female), high-density lipoprotein cholesterol (HDL) < 1.0/1.3 mmol/L for male/female, triglycerides ≥ 1.70 mmol/L or medication use, subclinical inflammation (i.e., C-reactive protein level >2 mg/L), pre-diabetes (defined as no self-reported T2DM and HbA1c ≥39 and <48 mmol/mol (Honigberg et al., 2021a)), and insulin resistance. The insulin resistance was not evaluated due to the unavailability of serum insulin data in the UK Biobank.

Moreover, MAFLD demonstrates considerable heterogeneity and is categorized into subtypes. Participants with a history of diabetes were classified as MAFLD-diabetes, while those without diabetes but with a BMI ≥ 25 kg/m^2^ were defined as MAFLD-overweight/obesity. Lean individuals without diabetes but with ≥ 2 metabolic dysregulations were classified as MAFLD-lean.

### 2.3 Environmental exposure assessment

We obtained average yearly concentrations of PM_10_, PM_2.5_, NO_x_, and NO_2_ from the UK’s Department for Environment, Food and Rural Affairs (DEFRA), which provides high-resolution near-surface air pollution data in the UK from 2002 to 2022[15]. Annual pollutant concentration maps were constructed on a 1 km × 1 km grid using an air dispersion model that integrates various sources from the National Atmospheric Emissions Inventory, combining measurement data for secondary inorganic aerosol and models for sources such as dust resuspension. The concentrations were calibrated using measured values from background sites within DEFRA’s Automatic Urban and Rural Network. The agreement between measured and modeled concentrations demonstrated good predictive ability, as quantified by R^2^ values of 0.85, 0.67, 0.80, and 0.60 for NO_2_, NO_x_, PM_2.5_, and PM_10_, respectively (UK’s Department for Environment, Food and Rural Affairs, 2022).

To evaluate each participant's exposure to air pollutants, we gathered their residential address history, including the dates of residence at each location. We estimated annual mean air pollution levels at each residence by assigning the current year's mean pollutant concentration to participants based on the 1 km × 1 km grid cells where they resided, utilizing bilinear interpolation. For time-varying exposure, we computed average concentrations of the air pollutants over the three years preceding the investigation date (3-year moving average concentrations) (Ai et al., 2024). The duration of residence at each location was considered, weighing the exposure by the length of time that participants lived at each address.

### 2.4 Outcomes

The endpoints of interest were the occurrence of intrahepatic disease, atherosclerotic cardiovascular disease, renal disease, or all-cause mortality. Intrahepatic disease was defined as cirrhosis or liver cancer; atherosclerotic cardiovascular disease was defined as coronary artery disease (CAD), ischemic stroke (IS), or peripheral artery disease (PAD); renal disease was defined as chronic kidney disease (CKD). Hospital admissions data were sourced from Hospital Episode Statistics for England, Patient Episode Database for Wales, and Scottish Morbidity Record. Death data were available until November 30, 2023 for participants in England and Wales, and until December 31, 2023 for participants in Scotland. Algorithmic defined cases were identified using algorithms developed by the UK Biobank Outcome Adjudication group. These algorithms utilized data from multiple sources, including self-reported information, hospital admission records, and death registers (UK Biobank, 2022). Follow-up time was calculated from the date of recruitment to the diagnosis of outcomes, the date of death, or the censoring date, whichever occurred first. Incident cases were primarily identified using the International Classification of Diseases, 10th edition (ICD-10) or Surveys: Classification of Interventions and Procedures (OPCS-4) codes (Supplemental Table 1) (Honigberg et al., 2021b).

### 2.5 Covariates

Covariates were chosen based on previous studies (Guo et al., 2022, Luo et al., 2022) and were determined using a directed acyclic graph (DAG) created with DAGitty’s online tool (www.dagitty.net) (Supplemental Figure 1). We specifically considered factors such as age, sex (male/female), and ethnicity (White/non-White). Alcohol consumption was categorized as never, low-to-moderate, and high, with high alcohol intake defined as a daily consumption of 30/20 g for men/women. Smoking status was categorized as never, previous, or current. The diet score was derived from a combination of vegetable, fruit, fish, unprocessed red meat, and processed meat intake, classified as poor (score 0–1), intermediate (score 2–3), or healthy (score 4–5). Physical activity were measured in weekly total metabolic equivalent task (MET) minutes: low (0 to <600 MET-minutes/week), medium (600 to <1200 MET-minutes/week), and high (≥1200 MET-minutes/week). Other covariates included annual household income (less than £18,000, £18,000–51,999, and greater than £52,000), education (college education, any school degree, vocational degree, and other), and the Townsend Deprivation Index (TDI). TDI is a composite measure of material deprivation that encompasses factors such as unemployment, lack of car ownership, non-home ownership, and household overcrowding (Townsend, 1987). It serves as an indicator of socioeconomic disadvantage, which is linked to both exposure to environmental risk factors and adverse health outcomes, including metabolic diseases (Jiang et al., 2023, Young et al., 2012). Missing data were imputed by calculating means for continuous variables or adding a missing indicator for categorical variables.

### 2.6 Statistical analysis

The baseline characteristics were described as means and standard deviations (SD) for continuous variables, and as counts with percentages for categorical variables, stratified by MAFLD subtypes. Time-varying Cox proportional hazards models were utilized to assess the associations between long-term air pollutant exposure and MAFLD subtypes, as well as their joint effects on health outcomes. Annual average pollutant concentrations were calculated as a 3-year moving average. Unlike conventional methods that use cumulative exposures or fixed values (such as the baseline year exposure), this approach minimizes potential exposure bias during long-term follow-ups periods (Liu et al., 2022a). The proportional hazards assumption was verified using Schoenfeld residuals, revealing no significant violations. Results were presented as hazard ratios (HRs) with 95 % confidence intervals (CIs). The models were adjusted for variables such as age, sex, ethnicity, alcohol consumption, smoking habits, diet score, physical activity level, annual household income, education, and the TDI.

We further explored potential additive and multiplicative interactions between air pollutant exposure and MAFLD. For the additive interactions, a term with 12 categories was created to reflect MAFLD subtypes and the tertiles of pollutant exposure (Overall tertile for PM_2.5_ was low: <8.93 μg/m^3^, intermediate: 8.93–10.70 μg/m^3^, and high: >10.70 μg/m^3^; for PM_10_ was low: <13.36 μg/m^3^, intermediate: 13.36–15.67 μg/m^3^, and high: >15.67 μg/m^3^; for NO_2_ was low: <14.65 μg/m^3^, intermediate: 14.65–19.89 μg/m^3^, and high: >19.89 μg/m^3^; for NO_x_ was low: <20.61 μg/m^3^, intermediate: 20.61–29.46 μg/m^3^, and high: >29.46 μg/m^3^). The detail information of pollutant tertiles was displayed in Supplemental Table 2.

The relative excess risk due to interaction (RERI) and attributable proportion (AP) were calculated, along with 95 % CIs (Chu et al., 2011). To investigate whether air pollutants modified the associations between MAFLD subtypes and health risk on a multiplicative scale, we calculated *p* for interaction by incorporating an interaction term into the model. Cumulative incidences were assessed using the cumulative incidence function provided in the ‘survminer’ R package. Additionally, restricted cubic splines with three knots were applied to model the concentration-response relationship.

Several sensitivity analyses were conducted to verify the robustness of the models by (1) excluding events within the first two years of follow-up; (2) additionally adjusting for the inflammation biomarker [C-reactive protein (CRP)], the circulating lipids biomarkers (triglycerides and HDL cholesterol), or estimated Glomerular Filtration Rate (eGFR) to evaluate whether the associations could be partially explained by systemic inflammation, blood lipids, or renal function; (3) altering the exposure time frame from the 3-year to 1-year or 5-year averages; (4) constructing two-pollutant models that included other air pollutants in the same analyses (e.g., adjusting PM_2.5_ for NO_2_ or NO_x_, and NO_2_ for PM_2.5_ or PM_10_).

All the analyses were performed using R software (version 4.1.1), with statistical significance was set at a two-sided p-value < 0.05.

## 3 Results

### 3.1 Descriptive results

The UK Biobank initially recruited 502,411 participants. After excluding those with pre-existing diseases, 152,421 out of 434,417 were diagnosed with MAFLD (Supplemental Figure 2). The included participants generally exhibited similar demographic characteristics, lifestyle behaviors, clinical traits, and laboratory measurements compared to both the overall population and the excluded participants (Supplemental Table 3). Over a median follow-up of 12.6 years, 13,789 participants had developed further liver diseases, including cirrhosis and liver cancer. Additionally, there were 45,305 participants developed incident atherosclerotic cardiovascular disease such as CAD (n = 33,168), IS (n = 6678), and PAD (n = 5459), 18,180 participants experienced CKD and 32,737 individuals died during the follow-up. Participants with MAFLD were generally older, predominantly male, and exhibited higher BMI or waist circumference compared to those without MAFLD (Table 1). In addition, individuals with MAFLD were more likely to consume alcohol and smoke, while also maintaining poorer dietary habits and engaging in lower levels of physical activity. The serum levels of laboratory biomarkers were also elevated in participants with MAFLD, expect for HDL-cholesterol. The average concentrations of air pollutants among all participants were as follows: PM_2.5_ at 9.87 μg/m^3^, PM_10_ at 14.66 μg/m^3^, NO_2_ at 17.93 μg/m^3^, and NO_x_ at 26.76 μg/m^3^, respectively (Supplemental Table 2).

**Table 1:** Basic characteristics of participants by the status of MAFLD.

	**Overall**	**Non-MAFLD**	**MAFLD-diabetes**	**MAFLD-lean**	**MAFLD-overweight/obesity**	***p*****Value**
**No. of participants, n**	434417	274817	15325	3341	140934	
***Demographics***						
Age (years), mean (SD)	56.16 (8.08)	55.80 (8.20)	58.85 (7.17)	56.80 (7.88)	56.56 (7.88)	< 0.001
Sex, %						< 0.001
Female	241306 (55.5)	181851 (66.2)	5770 (37.7)	492 (14.7)	53193 (37.7)	
Male	193111 (44.5)	92966 (33.8)	9555 (62.3)	2849 (85.3)	87741 (62.3)	
Ethnicity, %						< 0.001
White	409977 (94.4)	259525 (94.4)	13582 (88.6)	3123 (93.5)	133747 (94.9)	
Nonwhite	22462 (5.2)	14136 (5.1)	1632 (10.6)	200 (6.0)	6494 (4.6)	
Missing	1978 (0.5)	1156 (0.4)	111 (0.7)	18 (0.5)	693 (0.5)	
Household income, %						< 0.001
Less than 18,000	79900 (18.4)	46551 (16.9)	4447 (29.0)	743 (22.2)	28159 (20.0)	
18,000–51,999	193083 (44.4)	122013 (44.4)	6303 (41.1)	1476 (44.2)	63291 (44.9)	
Greater than 52,000	99550 (22.9)	66725 (24.3)	1990 (13.0)	682 (20.4)	30153 (21.4)	
Missing	61884 (14.2)	39528 (14.4)	2585 (16.9)	440 (13.2)	19331 (13.7)	
Education, %						< 0.001
College education	144348 (33.2)	100440 (36.5)	3531 (23.0)	1058 (31.7)	39319 (27.9)	
Any school degree	165617 (38.1)	105177 (38.3)	5262 (34.3)	1225 (36.7)	53953 (38.3)	
Vocational qualifications	27948 (6.4)	14839 (5.4)	1363 (8.9)	300 (9.0)	11446 (8.1)	
Other	91602 (21.1)	51594 (18.8)	4848 (31.6)	720 (21.6)	34440 (24.4)	
Missing	4902 (1.1)	2767 (1.0)	321 (2.1)	38 (1.1)	1776 (1.3)	
Townsend deprivation index, mean (SD)	−1.36 (3.06)	−1.51 (2.98)	−0.42 (3.41)	−1.11 (3.17)	−1.18 (3.13)	< 0.001
***Lifestyle behaviors***						
Alcohol consumption, %						< 0.001
Never	42074 (9.7)	25766 (9.4)	2745 (17.9)	232 (6.9)	13331 (9.5)	
Low to moderate	235442 (54.2)	156799 (57.1)	6761 (44.1)	1558 (46.6)	70324 (49.9)	
Heavy	93327 (21.5)	53137 (19.3)	2750 (17.9)	1245 (37.3)	36195 (25.7)	
Missing	63574 (14.6)	39115 (14.2)	3069 (20.0)	306 (9.2)	21084 (15.0)	
Smoking status, %						< 0.001
Never	240964 (55.5)	162256 (59.0)	6889 (45.0)	1461 (43.7)	70358 (49.9)	
Previous	146304 (33.7)	84522 (30.8)	6557 (42.8)	1159 (34.7)	54066 (38.4)	
Current	45097 (10.4)	26913 (9.8)	1725 (11.3)	708 (21.2)	15751 (11.2)	
Missing	2052 (0.5)	1126 (0.4)	154 (1.0)	13 (0.4)	759 (0.5)	
Diet score, %						< 0.001
0–1	49727 (11.4)	25655 (9.3)	2096 (13.7)	652 (19.5)	21324 (15.1)	
2–3	202559 (46.6)	123400 (44.9)	7396 (48.3)	1727 (51.7)	70036 (49.7)	
4–5	164013 (37.8)	115775 (42.1)	4774 (31.2)	786 (23.5)	42678 (30.3)	
Missing	18118 (4.2)	9987 (3.6)	1059 (6.9)	176 (5.3)	6896 (4.9)	
Physical active, %						< 0.001
Low	64717 (14.9)	34312 (12.5)	3470 (22.6)	575 (17.2)	26360 (18.7)	
Medium	143792 (33.1)	91702 (33.4)	4718 (30.8)	1173 (35.1)	46199 (32.8)	
High	143618 (33.1)	98472 (35.8)	3672 (24.0)	976 (29.2)	40498 (28.7)	
Missing	82290 (18.9)	50331 (18.3)	3465 (22.6)	617 (18.5)	27877 (19.8)	
***Clinical characteristics***						
FLI, mean (SD)	46.95 (29.95)	27.24 (16.85)	86.52 (11.14)	69.37 (7.60)	80.54 (11.34)	< 0.001
Waist circumference (cm), mean (SD)	89.74 (13.28)	82.49 (8.90)	107.89 (11.80)	90.98 (5.52)	101.89 (9.35)	< 0.001
BMI (kg/m^2^), mean (SD)	27.28 (4.73)	24.88 (2.85)	33.45 (5.36)	24.09 (0.85)	31.36 (4.20)	< 0.001
Hypertension, %	300895 (69.3)	166896 (60.7)	14086 (91.9)	2629 (78.7)	117284 (83.2)	< 0.001
Pre-diabetes, %	56477 (13.7)	25723 (9.9)	3799 (25.9)	464 (13.9)	26491 (19.8)	< 0.001
Diabetes, %	21676 (5.0)	6351 (2.3)	15325 (100.0)	0 (0.0)	0 (0.0)	< 0.001
***Laboratory measurements***						
TG (mmol/L), mean (SD)	1.73 (1.02)	1.34 (0.62)	2.47 (1.32)	3.18 (1.45)	2.38 (1.18)	< 0.001
GGT (U/L), mean (SD)	36.37 (39.78)	25.84 (19.21)	60.23 (64.21)	111.74 (125.06)	52.53 (51.63)	< 0.001
HbA1c (mmol/mol), mean (SD)	35.83 (6.31)	34.84 (4.83)	54.07 (14.55)	34.99 (3.83)	35.79 (3.93)	< 0.001
HDL-cholesterol (mmol/L), mean (SD)	1.46 (0.38)	1.58 (0.38)	1.15 (0.27)	1.33 (0.37)	1.27 (0.29)	< 0.001
CRP (mg/L), mean (SD)	2.53 (4.23)	1.92 (3.77)	4.17 (5.48)	2.83 (4.70)	3.53 (4.64)	< 0.001

Values are mean SD or n (%). Characteristics were compared by MAFLD status using Pearson chi-squared test for categorical variables, and analysis of variance test for continuous variables.

**Abbreviation:** MAFLD, metabolic-associated fatty liver disease; FLI, fatty liver index; BMI, body mass index; TG, triglyceride; GGT, gamma-glutamyltransferase; HbA1c, glycated hemoglobin; HDL: high density lipoprotein; CRP: C-reactive protein.

### 3.2 Associations of MAFLD subtypes and air pollutants exposure with intrahepatic and extrahepatic morbidity and all-cause mortality

After adjusting for potential covariates, individuals with MAFLD-diabetes subtype exhibited highest HRs and 95 % CIs compared to non-MAFLD individuals (Supplemental Table 4). The MAFLD-overweight/obesity subtype was significantly associated with all study outcomes, while the MAFLD-lean subtype demonstrated significant associations with cirrhosis (HR = 2.97, 95 % CI: 2.56–3.44), CAD (HR = 1.33, 95 % CI: 1.19–1.48), PAD (HR = 1.51, 95 % CI: 1.22–1.88), and all-cause mortality (HR = 1.45, 95 % CI: 1.32–1.60).

An interquartile range (IQR) increase in the four air pollutants were variably associated with elevated risks outcomes, depending on MAFLD subtypes. The association between liver disease and air pollution was stronger in those with the MAFLD-lean subtype, although some associations with liver cancer were not statistically significant. For ASCVD, significantly higher associations for CAD were observed with PM_2.5_ [HR (95 % CI): 1.07 (1.00, 1.14)] and PM_10_ [HR (95 % CI): 1.08 (1.02, 1.15)] in individuals with the MAFLD-diabetes subtype. Consistent positive associations were found for all four air pollutants with increased CKD risk in the MAFLD-diabetes subtype.

Restricted cubic spline models indicated an increasing association of PM_2.5_ and PM_10_ with the risk of cirrhosis, liver cancer, CKD, and all-cause mortality in MAFLD participants (Supplemental Figure 3). Additionally, increasing associations of NO_2_ and NO_x_ were observed with CAD risk, while the associations with cirrhosis and CKD exhibited a flatter slope at higher ranges.

### 3.3 Joint association of air pollutants exposure and MAFLD subtypes with intrahepatic and extrahepatic morbidity and all-cause mortality

**Fig. 1:**
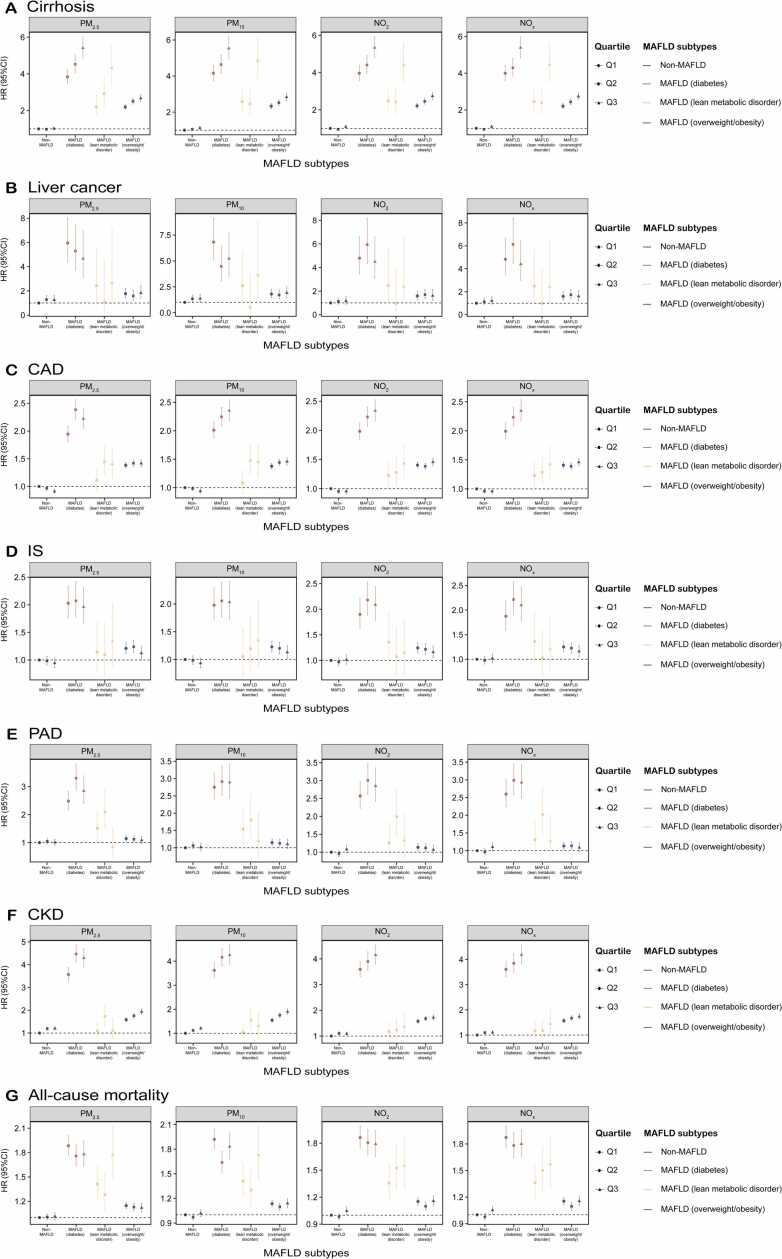
**The joint impact of long-term air pollutants exposure and different subtypes of MAFLD on (A) cirrhosis, (B) liver cancer, (C) CAD, (D) IS, (E) PAD, (F) CKD, and (G) all-cause mortality**. PM_2.5_, PM_10_, NO_2_, and NO_x_ were divided into quartile. The non-MAFLD participants with low quartile of air pollutants was set as the reference group. The hazard ratios and 95 % confidence intervals were calculated using the time-varying Cox proportional hazard regression models adjusted for age, sex, ethnicity, alcohol consumption, smoking status, diet score, physical activity, annual household income, education, and Townsend Deprivation Index. **Abbreviation:** MAFLD, metabolic-associated fatty liver disease; PM_2.5_, fine particulate matter with diameter < 2.5 μm; PM_10_, particulate matter with diameter < 10 μm; NO_2_, nitrogen dioxide; NO_x_, nitrogen oxides; CAD, coronary artery disease; IS, ischemic stroke; PAD, peripheral artery disease; CKD, chronic kidney disease.

**Fig. 2:**
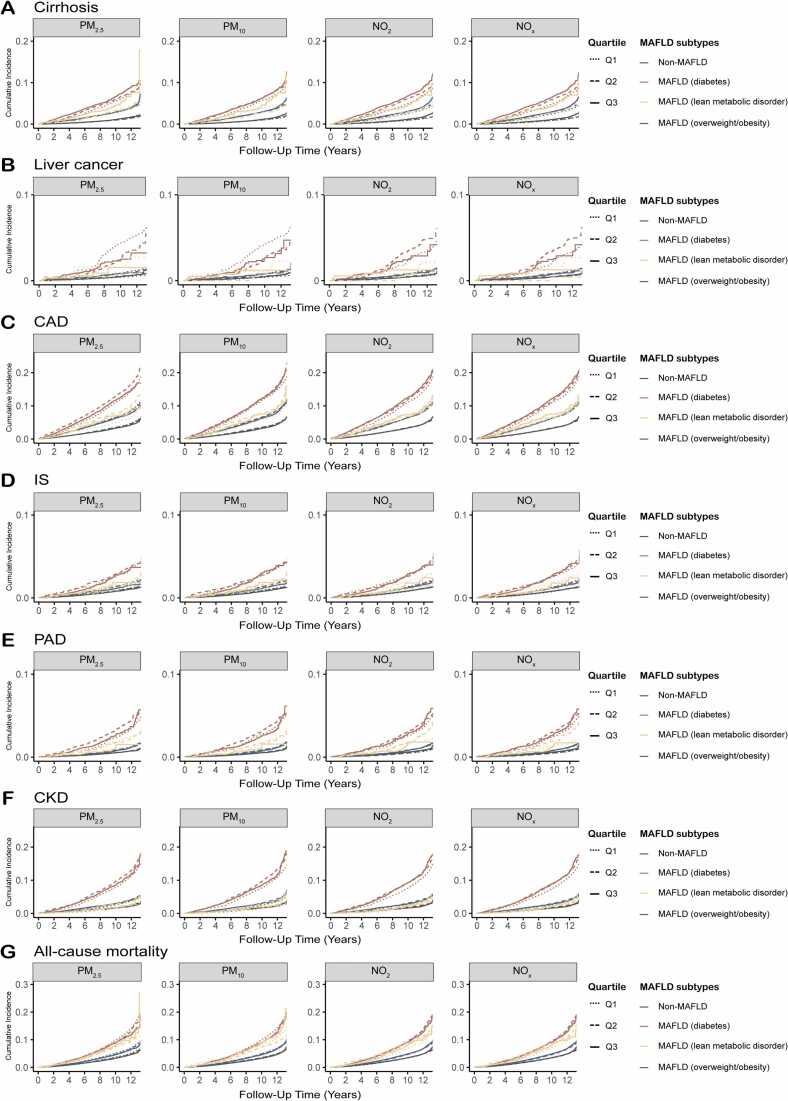
**The cumulative risks of (A) cirrhosis, (B) liver cancer, (C) CAD, (D) IS, (E) PAD, (F) CKD, and (G) all-cause mortality among the UK Biobank population by MAFLD status and long-term air pollutants exposure.** PM_2.5_, PM_10_, NO_2_, and NO_x_ were divided into quartile. The non-MAFLD participants with low quartile of air pollutants was set as the reference group. **Abbreviation:** MAFLD, metabolic-associated fatty liver disease; PM_2.5_, fine particulate matter with diameter < 2.5 μm; PM_10_, particulate matter with diameter < 10 μm; NO_2_, nitrogen dioxide; NO_x_, nitrogen oxides; CAD, coronary artery disease; IS, ischemic stroke; PAD, peripheral artery disease; CKD, chronic kidney disease.

We further evaluated the joint effects of air pollutants exposure and MAFLD subtypes on the risk of developing intrahepatic and extrahepatic morbidity, as well as all-cause mortality (Fig. 1 and Supplemental Table 5). Participants with higher air pollutant exposure and the MAFLD-diabetes subtype experienced a greater risk of developing health issues and mortality compared to those with lower exposure and non-MAFLD. Additionally, individuals with MAFLD-lean and higher air pollution exposure exhibited significantly increased risks of cirrhosis (PM_2.5_: HR = 4.32, 95 % CI: 3.39–5.51; PM_10_: HR = 4.86, 95 % CI: 3.83–6.17; NO_2_: HR = 4.42, 95 % CI: 3.50–5.58; NO_x_: HR = 4.47, 95 % CI: 3.54–5.63) and all-cause mortality (PM_2.5_: HR = 1.77, 95 % CI: 1.48–2.13; PM_10_: HR = 1.73, 95 % CI: 1.44–2.08; NO_2_: HR = 1.55, 95 % CI: 1.29–1.87; NO_x_: HR = 1.57, 95 % CI: 1.30, 1.89). This was followed by those in the MAFLD-overweight/obesity group compared to non-MAFLD participants. The cumulative incidence curve based on MAFLD subtypes also showed a similar trend (Fig. 2).

Significant interactions were observed between higher air pollutant exposure and all MAFLD subtypes concerning the risk of cirrhosis and CAD on both additive and multiplicative scales (Table 2). Based on the RERI, there was evidence of a joint effect larger than the sum of effects (positive additive) of air pollution exposure and MAFLD subtypes on the risk of cirrhosis, CAD and CKD. Notably, those with MAFLD-lean and high pollutant exposure had relatively heightened risks of cirrhosis, with RERIs of 2.10 (0.94, 3.26) for PM_2.5_, 2.16 (0.87, 3.45) for PM_10_, 1.85 (0.67, 3.04) for NO_2_, and 1.92 (0.74, 3.10), respectively. The risks of CAD were also elevated, with RERIs of 0.38 (0.04, 0.72) associated with PM_2.5_ and 0.44 (0.09, 0.78) associated with PM_10_. Furthermore, a higher risk of CKD was found in participants with MAFLD-diabetic and increased air pollutant exposure on the additive scale.

**Table 2:** Additive and multiplicative interactions between air pollutants and MAFLD subtype on the risk of intrahepatic and extrahepatic morbidity and all-cause mortality.

**Air pollution**	**MAFLD subtype**								
	**MAFLD-diabetes**			**MAFLD-lean**			**MAFLD-overweight/obesity**		
	**RERI (95 % CI)**	**AP (95 % CI)**	***p*****for interaction**	**RERI (95 % CI)**	**AP (95 % CI)**	***p*****for interaction**	**RERI (95 % CI)**	**AP (95 % CI)**	***p*****for interaction**
**Cirrhosis**									
PM_2.5_									
Intermediate	**0.71 (0.14, 1.28)**	**0.16 (0.04, 0.27)**	**< 0.001**	0.74 (−0.21, 1.69)	0.25 (−0.02, 0.53)	**0.001**	**0.34 (0.18, 0.49)**	**0.13 (0.08, 0.19)**	**< 0.001**
High	**1.55 (0.91, 2.18)**	**0.29 (0.19, 0.38)**		**2.10 (0.94, 3.26)**	**0.49 (0.31, 0.66)**		**0.45 (0.28, 0.63)**	**0.17 (0.11, 0.23)**	
PM_10_									
Intermediate	0.43 (−0.15, 1.02)	0.09 (−0.03, 0.21)	**0.008**	−0.18 (−1.11, 0.75)	−0.07 (−0.47, 0.32)	**0.001**	0.14 (−0.01, 0.30)	0.06 (−0.00, 0.12)	**0.011**
High	**1.26 (0.60, 1.92)**	**0.23 (0.13, 0.33)**		**2.16 (0.87, 3.45)**	**0.44 (0.26, 0.63)**		**0.38 (0.20, 0.56)**	**0.13 (0.07, 0.19)**	
NO_2_									
Intermediate	0.49 (−0.08, 1.07)	0.11 (−0.01, 0.23)	**0.004**	−0.00 (−0.92, 0.91)	−0.00 (−0.38, 0.38)	**0.001**	**0.30 (0.14, 0.45)**	**0.12 (0.06, 0.18)**	**0.011**
High	**1.28 (0.66, 1.91)**	**0.24 (0.14, 0.34)**		**1.85 (0.67, 3.04)**	**0.42 (0.23, 0.61)**		**0.44 (0.26, 0.61)**	**0.16 (0.10, 0.22)**	
NO_x_									
Intermediate	0.35 (−0.22, 0.92)	0.08 (−0.04, 0.21)	**0.006**	−0.02 (−0.93, 0.89)	−0.01 (−0.39, 0.38)	**0.001**	**0.29 (0.14, 0.44)**	**0.12 (0.06, 0.18)**	**0.014**
High	**1.31 (0.68, 1.94)**	**0.24 (0.14, 0.34)**		**1.92 (0.74, 3.10)**	**0.43 (0.24, 0.62)**		**0.44 (0.27, 0.61)**	**0.16 (0.10, 0.22)**	
**Liver cancer**									
PM_2.5_									
Intermediate	−0.95 (−3.15, 1.25)	−0.18 (−0.63, 0.27)	**0.032**	−1.65 (−4.15, 0.86)	−1.53 (−5.49, 2.43)	0.727	−0.47 (−0.99, 0.04)	−0.30 (−0.65, 0.05)	0.241
High	−1.55 (−3.78, 0.68)	−0.33 (−0.90, 0.23)		−0.06 (−3.31, 3.20)	−0.02 (−1.27, 1.23)		−0.19 (−0.74, 0.36)	−0.10 (−0.41, 0.21)	
PM_10_									
Intermediate	−2.69 (−4.94, −0.43)	−0.60 (−1.23, 0.03)	**0.008**	−2.44 (−4.86, −0.02)	−4.70 (−16.59, 7.19)	0.644	−0.44 (−0.97, 0.09)	−0.26 (−0.58, 0.07)	0.139
High	−1.95 (−4.42, 0.52)	−0.37 (−0.94, 0.19)		0.64 (−3.17, 4.46)	0.18 (−0.75, 1.11)		−0.25 (−0.83, 0.33)	−0.13 (−0.45, 0.19)	
NO_2_									
Intermediate	1.02 (−1.07, 3.11)	0.17 (−0.15, 0.49)	0.356	−1.65 (−4.08, 0.78)	−1.72 (−6.04, 2.59)	0.981	−0.02 (−0.48, 0.45)	−0.01 (−0.29, 0.26)	0.240
High	−0.48 (−2.49, 1.53)	−0.11 (−0.58, 0.37)		−0.25 (−3.33, 2.84)	−0.10 (−1.47, 1.26)		−0.15 (−0.65, 0.36)	−0.09 (−0.41, 0.23)	
NO_x_									
Intermediate	1.17 (−0.95, 3.29)	0.19 (−0.12, 0.50)	0.342	−1.65 (−4.10, 0.80)	−1.71 (−6.00, 2.59)	0.959	0.01 (−0.46, 0.48)	0.01 (−0.26, 0.28)	0.217
High	−0.63 (−2.65, 1.38)	−0.14 (−0.64, 0.35)		−0.29 (−3.40, 2.82)	−0.12 (−1.50, 1.26)		−0.20 (−0.71, 0.31)	−0.13 (−0.45, 0.20)	
**CAD**									
PM_2.5_									
Intermediate	**0.47 (0.27, 0.68)**	**0.20 (0.12, 0.28)**	**< 0.001**	**0.36 (0.04, 0.68)**	**0.25 (0.06, 0.44)**	**0.006**	**0.07 (0.00, 0.13)**	**0.05 (0.00, 0.09)**	**< 0.001**
High	**0.37 (0.16, 0.57)**	**0.17 (0.08, 0.25)**		**0.38 (0.04, 0.72)**	**0.27 (0.07, 0.47)**		**0.12 (0.06, 0.19)**	**0.09 (0.04, 0.13)**	
PM_10_									
Intermediate	**0.25 (0.05, 0.45)**	**0.11 (0.03, 0.20)**	**< 0.001**	**0.41 (0.10, 0.73)**	**0.28 (0.10, 0.46)**	**0.025**	**0.08 (0.02, 0.15)**	**0.06 (0.01, 0.10)**	**< 0.001**
High	**0.41 (0.19, 0.62)**	**0.17 (0.09, 0.26)**		**0.44 (0.09, 0.78)**	**0.30 (0.11, 0.49)**		**0.14 (0.07, 0.21)**	**0.10 (0.05, 0.14)**	
NO_2_									
Intermediate	**0.29 (0.09, 0.49)**	**0.13 (0.05, 0.22)**	**< 0.001**	0.10 (−0.22, 0.42)	0.08 (−0.16, 0.32)	**0.034**	0.03 (−0.04, 0.09)	0.02 (−0.03, 0.06)	**< 0.001**
High	**0.40 (0.18, 0.61)**	**0.17 (0.09, 0.25)**		0.25 (−0.09, 0.60)	0.18 (−0.04, 0.40)		**0.10 (0.03, 0.17)**	**0.07 (0.02, 0.11)**	
NO_x_									
Intermediate	**0.28 (0.08, 0.48)**	**0.13 (0.04, 0.21)**	**< 0.001**	0.10 (−0.22, 0.42)	0.08 (−0.16, 0.31)	**0.044**	0.02 (−0.04, 0.09)	0.02 (−0.03, 0.06)	**< 0.001**
High	**0.39 (0.18, 0.61)**	**0.17 (0.09, 0.25)**		0.23 (−0.11, 0.58)	0.16 (−0.06, 0.39)		**0.09 (0.02, 0.16)**	**0.06 (0.02, 0.11)**	
**IS**									
PM_2.5_									
Intermediate	0.06 (−0.35, 0.46)	0.03 (−0.17, 0.22)	0.933	−0.03 (−0.66, 0.60)	−0.03 (−0.62, 0.56)	0.683	0.04 (−0.09, 0.18)	0.04 (−0.07, 0.14)	0.641
High	−0.01 (−0.42, 0.40)	−0.01 (−0.22, 0.20)		0.25 (−0.45, 0.95)	0.19 (−0.28, 0.65)		−0.03 (−0.17, 0.11)	−0.03 (−0.15, 0.10)	
PM_10_									
Intermediate	0.09 (−0.31, 0.49)	0.04 (−0.15, 0.23)	0.905	0.16 (−0.47, 0.78)	0.13 (−0.36, 0.62)	0.614	−0.01 (−0.14, 0.12)	−0.01 (−0.12, 0.10)	0.443
High	0.12 (−0.30, 0.53)	0.06 (−0.14, 0.26)		0.36 (−0.34, 1.06)	0.26 (−0.17, 0.70)		−0.03 (−0.17, 0.11)	−0.03 (−0.15, 0.10)	
NO_2_									
Intermediate	0.31 (−0.10, 0.73)	0.14 (−0.03, 0.32)	0.505	−0.25 (−0.92, 0.41)	−0.24 (−0.93, 0.45)	0.969	0.00 (−0.13, 0.14)	0.00 (−0.11, 0.11)	0.321
High	0.17 (−0.24, 0.59)	0.08 (−0.11, 0.27)		−0.22 (−0.92, 0.49)	−0.19 (−0.87, 0.49)		−0.09 (−0.24, 0.05)	−0.08 (−0.21, 0.05)	
NO_x_									
Intermediate	0.36 (−0.05, 0.78)	0.16 (−0.01, 0.34)	0.589	−0.31 (−0.98, 0.35)	−0.31 (−1.04, 0.43)	0.976	−0.00 (−0.14, 0.13)	−0.00 (−0.11, 0.11)	0.294
High	0.21 (−0.20, 0.62)	0.10 (−0.09, 0.29)		−0.17 (−0.89, 0.55)	−0.14 (−0.78, 0.50)		−0.10 (−0.25, 0.04)	−0.09 (−0.22, 0.04)	
**PAD**									
PM_2.5_									
Intermediate	**0.77 (0.25, 1.30)**	**0.23 (0.10, 0.37)**	0.393	0.54 (−0.31, 1.39)	0.26 (−0.08, 0.59)	0.194	−0.07 (−0.22, 0.08)	−0.06 (−0.20, 0.07)	0.259
High	0.36 (−0.19, 0.90)	0.12 (−0.05, 0.30)		−0.68 (−1.38, 0.02)	−0.80 (−2.02, 0.41)		−0.07 (−0.23, 0.09)	−0.06 (−0.22, 0.09)	
PM_10_									
Intermediate	0.09 (−0.42, 0.61)	0.03 (−0.14, 0.21)	0.614	0.20 (−0.59, 0.99)	0.11 (−0.30, 0.52)	0.303	−0.08 (−0.23, 0.06)	−0.07 (−0.21, 0.06)	0.209
High	0.12 (−0.44, 0.69)	0.04 (−0.15, 0.23)		−0.36 (−1.15, 0.43)	−0.30 (−1.08, 0.48)		−0.06 (−0.22, 0.11)	−0.05 (−0.20, 0.10)	
NO_2_									
Intermediate	0.48 (−0.04, 0.99)	**0.16 (0.00, 0.32)**	0.748	0.77 (−0.02, 1.56)	**0.39 (0.08, 0.69)**	0.526	0.02 (−0.12, 0.17)	0.02 (−0.11, 0.15)	**0.040**
High	0.20 (−0.34, 0.74)	0.07 (−0.11, 0.25)		−0.02 (−0.77, 0.73)	−0.01 (−0.59, 0.56)		−0.15 (−0.31, 0.01)	−0.14 (−0.29, 0.02)	
NO_x_									
Intermediate	0.42 (−0.09, 0.94)	0.14 (−0.02, 0.30)	0.707	0.74 (−0.06, 1.55)	**0.37 (0.06, 0.68)**	0.509	0.04 (−0.11, 0.18)	0.03 (−0.09, 0.16)	0.061
High	0.22 (−0.32, 0.77)	0.08 (−0.10, 0.26)		−0.14 (−0.89, 0.61)	−0.11 (−0.74, 0.52)		−0.15 (−0.31, 0.01)	−0.14 (−0.29, 0.02)	
**CKD**									
PM_2.5_									
Intermediate	**0.71 (0.28, 1.14)**	**0.16 (0.07, 0.25)**	0.704	0.44 (−0.13, 1.01)	0.26 (−0.02, 0.53)	0.894	−0.02 (−0.13, 0.09)	−0.01 (−0.08, 0.05)	0.423
High	**0.53 (0.07, 0.98)**	**0.12 (0.03, 0.22)**		−0.18 (−0.72, 0.35)	−0.17 (−0.70, 0.37)		**0.15 (0.03, 0.28)**	**0.08 (0.02, 0.14)**	
PM_10_									
Intermediate	0.42 (−0.01, 0.85)	**0.10 (0.00, 0.20)**	0.753	0.38 (−0.14, 0.90)	0.25 (−0.04, 0.54)	0.844	0.09 (−0.01, 0.20)	0.05 (−0.01, 0.11)	0.369
High	0.43 (−0.02, 0.89)	**0.10 (0.00, 0.20)**		0.06 (−0.50, 0.62)	0.05 (−0.37, 0.47)		**0.15 (0.03, 0.27)**	**0.08 (0.02, 0.14)**	
NO_2_									
Intermediate	0.20 (−0.22, 0.63)	0.05 (−0.05, 0.16)	0.254	−0.05 (−0.55, 0.45)	−0.04 (−0.46, 0.38)	0.490	−0.00 (−0.11, 0.10)	−0.00 (−0.06, 0.06)	0.743
High	**0.47 (0.05, 0.89)**	**0.11 (0.02, 0.21)**		0.10 (−0.47, 0.66)	0.07 (−0.32, 0.47)		0.05 (−0.06, 0.17)	0.03 (−0.04, 0.10)	
NO_x_									
Intermediate	0.15 (−0.28, 0.57)	0.04 (−0.07, 0.15)	0.265	−0.09 (−0.58, 0.40)	−0.08 (−0.51, 0.36)	0.515	0.01 (−0.09, 0.11)	0.01 (−0.06, 0.07)	0.668
High	**0.47 (0.05, 0.90)**	**0.11 (0.02, 0.21)**		0.17 (−0.40, 0.75)	0.12 (−0.25, 0.49)		0.06 (−0.05, 0.18)	0.04 (−0.03, 0.10)	
**All-cause mortality**									
PM_2.5_									
Intermediate	−0.13 (−0.30, 0.04)	−0.08 (−0.18, 0.03)	0.146	−0.14 (−0.44, 0.17)	−0.11 (−0.36, 0.15)	0.490	−0.03 (−0.08, 0.03)	−0.02 (−0.07, 0.03)	0.164
High	−0.12 (−0.30, 0.07)	−0.07 (−0.17, 0.04)		0.35 (−0.02, 0.72)	**0.20 (0.02, 0.38)**		−0.04 (−0.10, 0.02)	−0.04 (−0.09, 0.02)	
PM_10_									
Intermediate	−0.25 (−0.42, −0.08)	−0.15 (−0.27, −0.04)	0.241	−0.08 (−0.38, 0.22)	−0.06 (−0.30, 0.18)	0.422	−0.01 (−0.07, 0.05)	−0.01 (−0.06, 0.04)	0.208
High	−0.11 (−0.29, 0.08)	−0.06 (−0.16, 0.05)		0.30 (−0.08, 0.67)	0.17 (−0.02, 0.36)		−0.01 (−0.08, 0.05)	−0.01 (−0.07, 0.05)	
NO_2_									
Intermediate	−0.04 (−0.22, 0.14)	−0.02 (−0.12, 0.08)	0.352	0.19 (−0.13, 0.50)	0.12 (−0.07, 0.31)	0.361	−0.04 (−0.09, 0.02)	−0.03 (−0.09, 0.02)	**0.022**
High	−0.12 (−0.30, 0.06)	−0.07 (−0.17, 0.04)		0.15 (−0.20, 0.50)	0.09 (−0.12, 0.31)		−0.04 (−0.11, 0.02)	−0.04 (−0.09, 0.02)	
NO_x_									
Intermediate	−0.07 (−0.25, 0.11)	−0.04 (−0.14, 0.06)	0.471	0.16 (−0.15, 0.48)	0.11 (−0.09, 0.31)	0.349	−0.03 (−0.09, 0.02)	−0.03 (−0.09, 0.02)	**0.018**
High	−0.13 (−0.31, 0.05)	−0.07 (−0.17, 0.03)		0.16 (−0.20, 0.51)	0.10 (−0.11, 0.31)		−0.05 (−0.12, 0.01)	−0.04 (−0.10, 0.01)	

*P* for interaction in bold represent significance at *p* < 0.05. If 0 is outside the CIs of RERIs and APs, it means that there is a significant additive interaction and marked in bold. Models were adjusted for age, sex, ethnicity, alcohol consumption, smoking status, diet score, physical activity, annual household income, education, and Townsend Deprivation Index. PM_2.5_ (low <8.93 μg/m^3^, intermediate 8.93–10.70 μg/m^3^, high >10.70 μg/m^3^); PM_10_ (low <13.36 μg/m^3^, intermediate 13.36–15.67 μg/m^3^, high >15.67 μg/m^3^); NO_2_ (low <14.65 μg/m^3^, intermediate 14.65–19.89 μg/m^3^, high >19.89 μg/m^3^); NO_x_ (low <20.61 μg/m^3^, intermediate 20.61–29.46 μg/m^3^, high >29.46 μg/m^3^).

**Abbreviation:** MAFLD, metabolic-associated fatty liver disease; PM_2.5_, fine particulate matter with diameter ≤ 2.5 μm; PM_10_, particulate matter with diameter ≤ 10 μm; NO_2_, nitrogen dioxide; NO_x_, nitrogen oxides; CAD, coronary artery disease; IS, ischemic stroke; PAD, peripheral artery disease; CKD, chronic kidney disease; RERI, relative excess risk due to interaction; AP, attributable proportion.

### 3.4 Sensitivity analyses

In sensitivity analyses, the associations between long-term air pollutants exposure and the risk of intrahepatic and extrahepatic morbidity, as well as all-cause mortality remained consistent when we excluded participants diagnosed with outcomes within the first 2 years of follow-up (Supplemental Table 6), when further adjusting for circulating levels of TG, HDL cholesterol, CRP, and eGFR (Supplemental Table 7), when varying the exposure time window from 3 years to 1 or 5 years (Supplemental Table 8–9), and when applying two-pollutant models (Supplemental Table 10).

## 4 Discussion

To the best of our knowledge, this study represents the largest prospective cohort investigation examining the independent and combined effects of MAFLD subtypes and long-term exposure to air pollutants on multi-system morbidity and all-cause mortality within the UK Biobank population. Our findings indicate that MAFLD-diabetes carries the highest risk for all study outcomes, while MAFLD-lean is associated with increased risks of intrahepatic morbidity and all-cause mortality compared to MAFLD-overweight/obesity. Additionally, participants exposed to higher levels of air pollutants across all MAFLD subtypes exhibited greater risks of cirrhosis and CAD. Notably, MAFLD-diabetes also presented a higher risk of CKD than non-MAFLD participants who experienced lower levels of air pollutant exposure.

Both MAFLD and ambient air pollution pose significant public health challenges that contribute to elevated morbidity and mortality. Evolving from the previous term of NAFLD, MAFLD provides a more precise identification of individuals with hepatic fat accumulation and related metabolic disorders (Tilg and Effenberger, 2020). This refined classification highlights the association between MAFLD and increased risks for both intrahepatic and extrahepatic conditions, taking into account established risk factors. Among the various MAFLD subtypes, MAFLD-diabetes demonstrated the highest morbidity and mortality risk, consistent with its links to dyslipidemia, endothelial dysfunction, and prolonged hyperglycemia (Poznyak et al., 2020). These factors significantly contribute to complications such as cirrhosis, atherosclerosis, diabetic nephropathy, carcinogenesis, and overall mortality (Ali et al., 2022, Anders et al., 2018, Castera and Cusi, 2023, Shlomai et al., 2016). While individuals with MAFLD-overweight/obesity faced higher risks of extrahepatic morbidity, they had lower intrahepatic morbidity and mortality risks compared to those with MAFLD-lean. Supporting previous findings (Chen et al., 2023, Zhang et al., 2023b), MAFLD-overweight/obesity is significantly associated with atherosclerotic plaques, stenosis, and heightened risks of myocardial infarction and stroke. Data from the NHANES III study further indicated that individuals with MAFLD-diabetes (HR = 1.275, 95 CI%: 1.075–1.512) and MAFLD-lean (HR = 1.296, 95 CI%: 1.064–1.578) have higher all-cause mortality risks compared to those with MAFLD-overweight/obesity (HR = 0.992, 95 CI%: 0.893–1.102), indicating the variability in prognoses based on diagnostic criteria (Chen et al., 2021).

The adverse health effects of air pollution are well-documented. In our study, we found that increased exposure to four air pollutants significantly elevated the risks of cirrhosis, CAD, PAD, and CKD. Similarly, a previous study involving 456,687 UK residents reported that each IQR increase in PM_2.5_, PM_10_, NO_2_, NO_x_ was associated with HR of 1.21 (95 % CI: 1.03, 1.41), 1.27 (95 % CI: 1.06, 1.52), 1.39 (95 % CI: 1.16, 1.66) and 1.21 (95 % CI: 1.03, 1.43) for cirrhosis, respectively (Li et al., 2023). Additionally, Yuan et.al found that a 10 μg/m^3^ increase in PM_2.5_ was associated with 18 % increased risk of ASCVD (Yuan et al., 2023). Another time-varying analysis involving U.S. veteran showed a 10 µg/m^3^ increase in PM_2.5_ associated with 26–28 % higher risk of incident CKD, CKD progression, and development of end-stage renal disease (Bowe et al., 2018). Our findings align with these studies, highlighting air pollution as a significant risk factor for liver, cardiovascular, and renal diseases.

While previous research has explored the health effects of MAFLD subtypes and air pollution separately, this study is the first, to our knowledge, to assess their potential interactions. We found that the combined exposure to air pollution and MAFLD subtypes could synergistically increase the risks of cirrhosis, CAD, and CKD on either an additive or multiplicative scale. The shared biological pathways between air pollution and MAFLD render these interactions biologically plausible. Lipid accumulation leading to low-grade inflammation, insulin resistance, and oxidative stress are major causative factors of MAFLD (Badmus et al., 2022). Furthermore, a comprehensive lipidomic approach revealed that ambient PM_2.5_ exposure induces inflammation, increases oxidative stress, and worsens whole-body insulin sensitivity, affecting hepatic lipogenesis including species of triglyceride, ceramide, and sphingomyelin, which might be potential lipo-toxicity biomarkers contributing to metabolic dysfunction induced by PM_2.5_ (Li et al., 2020, Xu et al., 2019). These mechanisms help elucidate how air pollution interacts with MAFLD, thereby increasing the risk of liver, cardiovascular, and renal disease.

Our findings indicate that MAFLD-lean participants face a higher relative excess risk associated with air pollution, particularly concerning the further risk in cirrhosis and CAD. Previous studies suggested that beyond simple differences in BMI values, MAFLD-lean might represent a distinct pathophysiological entity compared to MAFLD-overweight/obesity. Individuals with MAFLD-lean often possess specific genetic profiles, such as PNPLA3 or TM6SF2 risk alleles, and unique gut microbiomes that contribute to fatty liver development (Chen et al., 2020). Additionally, metabolic adaptations in MAFLD-lean individuals, such as increased bile acid secretion and heightened farnesoid X receptor activity in response to an ‘obesogenic’ environment, might fail to prevent hepatic inflammation and fibrosis as liver disease progresses, ultimately leading to adverse long-term outcomes (Hagstrom et al., 2018). Metabolically obese individuals with normal weight might exhibit abnormal body fat distribution, insulin resistance, atherogenic dyslipidemia, and increased hepatic fat accumulation, elevating their risk for further diseases (Mathew et al., 2016). Therefore, MAFLD-lean patients should probably consider implementing protective measures against the risks of cirrhosis and CAD associated with air pollution.

The strengths of our study include large sample size, extended follow-up period, and consistent data collection, enhancing the robust and generalizable of our conclusions. Additionally, we utilized a highly detailed spatiotemporal model to assess air pollution, covering the follow-up period, contributing valuable insights into the impact of long-term air pollution exposure and MAFLD with the risk of related health outcomes. Our findings highlight the importance of air pollution prevention and control, particularly for individuals with MAFLD, who would benefit significantly from such measures. Future prevention strategies for MAFLD should prioritize modifiable risk factors, such as PM_2.5_ exposure, by implementing stricter air pollution control policies and promoting personal protective measures, including wearing masks, using air purifiers, and minimizing outdoor activities during high pollution periods (Yang et al., 2018, Zhang et al., 2023a). Enhancing management and intervention efforts for high-risk populations alongside overall improvements in air quality represents a promising approach for the secondary prevention of MAFLD. However, our findings should be considered within the context of several potential limitations. First, ambient air pollution estimates were based on home addresses, possibly overlooking indoor pollution and time spent in traffic, leading to exposure misclassification. Second, as an observational study, residual confounding from unknown or unmeasured factors cannot be completely ruled out. Third, there might be potential reverse causality; however, the consistency of results remained after excluding participants with outcomes occurring within two years of follow-up, supporting the robustness of our findings. Fourth, fatty liver diagnosis relied on serum biomarkers rather than imaging or biopsy. Nevertheless, the FLI has demonstrated acceptable sensitivity and specificity among Caucasians and has been externally validated and widely used in population-based study [39]. Finally, the UK Biobank primarily recruited Caucasians from developed countries with relatively low air pollution and better access to healthcare, indicating a need for further studies in diverse populations.

## 5 Conclusions

These findings highlight the importance of screening and preventing MAFLD, particularly the diabetic subtype, to reduce the risk of multi-system diseases and all-cause mortality. Our results also suggest that long-term exposure to air pollution might exacerbate the risks of further cirrhosis, CAD and CKD in MAFLD participants, underscoring the need for optimizing risk factor control for MAFLD.

## Ethical Approval

The project has approval from the North West Multi-Center Research Ethics Committee (MREC) (REC reference: 21/NW/0157), and informed written consent was obtained from each participant.

## Funding

This work was supported by the 10.13039/501100001809National Natural Science Foundation of China (82373534), and by the 10.13039/100000865Bill & Melinda Gates Foundation [Grant Number: INV-016826].

## CRediT authorship contribution statement

**Zhang Jingyi:** Writing – review & editing, Writing – original draft, Formal analysis, Conceptualization. **Ran Shanshan:** Writing – review & editing, Formal analysis. **Wei Shengtao:** Formal analysis. **Tian Fei:** Formal analysis. **Chen Lan:** Formal analysis. **Yang Zijun:** Formal analysis. **Chen Ge:** Writing – review & editing. **Lin Hualiang:** Writing – review & editing, Supervision, Funding acquisition, Conceptualization.

## Declaration of Competing Interest

The authors declare the following financial interests/personal relationships which may be considered as potential competing interests. Hualiang Lin reports financial support was provided by Bill & Melinda Gates Foundation

## Data Availability

Data from the UK Biobank (https://www.ukbiobank.ac.uk/) are available for researchers on application.
