# Supplementary material for: Associations of MAFLD subtypes and air pollutants with multi-system morbidity and all-cause mortality: A prospective cohort study
Source: Ecotoxicol Environ Saf. 2025 Feb;291:117893. doi: 10.1016/j.ecoenv.2025.117893 (PMC11860302; doi:10.1016/j.ecoenv.2025.117893)
Supplement: Supplementary file 1 — Supplementary material [file mmc1.docx]

**Supplemental Table 1. Definition of incident outcomes**

| **Outcome** | **ICD-10** | **OPCS-4** | **Biomarkers** | **Others** |
| --- | --- | --- | --- | --- |
| Cirrhosis | K72, K73, K74, K76 | - | - | - |
| Liver cancer | C22 | - | - | - |
| Coronary artery disease | I21, I22, I23, I24, I25 | K40, K41, K42, K43, K44, K45, K46, K49, K50, K75 | - | - |
| Ischemic stroke | - | - | - | Algorithmic outcomes |
| Peripheral artery disease | I70, I73.8, I73.9 | X09.3, X09.4, X09.5, L21.6, L51.3, L51.6, L51.8, L52.1, L52.2, L54.1, L54.4, L54.8, L59.1, L59.2, L59.3, L59.4, L59.5, L59.6, L59.7, L59.8, L60.1, L60.2, L63.1, L63.5, L63.9, L66.7 | - | - |
| Chronic kidney disease | N18 | - | eGFR<60 mL/min/1.73 m^2^ | - |

**Supplemental Table 2. Range of air pollutants concentrations**

| **Air pollutants** | **Mean (SD),** **μg/m^3^** | **IQR, μg/m^3^** | **T1, μg/m^3^** | **T2, μg/m^3^** | **T3, μg/m^3^** |
| --- | --- | --- | --- | --- | --- |
| **Overall** | | | | | |
| PM_2.5_ | 9.87 (2.07) | 2.76 | <8.93 | 8.93-10.70 | >10.70 |
| PM_10_ | 14.66 (2.74) | 3.59 | <13.36 | 13.36-15.67 | >15.67 |
| NO_2_ | 17.93 (6.58) | 8.16 | <14.65 | 14.65-19.89 | >19.89 |
| NO_x_ | 26.76 (11.97) | 13.87 | <20.61 | 20.61-29.46 | >29.46 |
| **Non-MAFLD** | | | | | |
| PM_2.5_ | 9.87 (2.10) | 2.79 | <8.92 | 8.92-10.71 | >10.71 |
| PM_10_ | 14.67 (2.78) | 3.64 | <13.35 | 13.35-15.68 | >15.68 |
| NO_2_ | 17.91 (6.68) | 8.24 | <14.56 | 14.56-19.84 | >19.84 |
| NO_x_ | 26.74 (12.17) | 13.96 | <20.48 | 20.48-29.39 | >29.39 |
| **MAFLD-diabetes** | | | | | |
| PM_2.5_ | 10.06 (2.08) | 2.75 | <9.12 | 9.12-10.89 | >10.89 |
| PM_10_ | 14.91 (2.74) | 3.61 | <13.61 | 13.61-15.93 | >15.93 |
| NO_2_ | 18.73 (6.66) | 8.50 | <15.38 | 15.38-20.86 | >20.86 |
| NO_x_ | 28.17 (12.23) | 14.71 | <21.77 | 21.77-31.20 | >31.20 |
| **MAFLD-lean** | | | | | |
| PM_2.5_ | 9.96 (2.04) | 2.72 | <9.04 | 9.04-10.80 | >10.80 |
| PM_10_ | 14.78 (2.70) | 3.57 | <13.52 | 13.52-15.78 | >15.78 |
| NO_2_ | 18.19 (6.57) | 8.12 | <14.96 | 14.96-20.17 | >20.17 |
| NO_x_ | 27.22 (12.04) | 13.93 | <21.14 | 21.14-29.96 | >29.96 |
| **MAFLD-overweight/obesity** | | | | | |
| PM_2.5_ | 9.83 (2.01) | 2.70 | <8.92 | 8.92-10.66 | >10.66 |
| PM_10_ | 14.61 (2.66) | 3.50 | <13.37 | 13.37-15.62 | >15.62 |
| NO_2_ | 17.88 (6.35) | 7.98 | <14.74 | 14.74-19.87 | >19.87 |
| NO_x_ | 26.63 (11.52) | 13.56 | <20.75 | 20.75-29.43 | >29.43 |

**Abbreviations:** SD, standard deviation; Interquartile Range; PM_2.5_, fine particulate matter with diameter <2.5 μm; PM_10_, particulate matter with diameter <10 μm; NO_2_, nitrogen dioxide; NO_x_, nitrogen oxides; T, tertile.

**Supplemental Table 3. Baseline characteristics of the overall, included and excluded participants**

|  | **Overall** | **Included** | **Excluded** |
| --- | --- | --- | --- |
| **No. of participants, n** | 502411 | 67994 | 434417 |
| ***Demographics*** |  |  |  |
| Age (years), mean (SD) | 56.53 (8.10) | 58.89 (7.77) | 56.16 (8.08) |
| Sex, % |  |  |  |
| Female | 273327 (54.4) | 32021 (47.1) | 241306 (55.5) |
| Male | 229084 (45.6) | 35973 (52.9) | 193111 (44.5) |
| Ethnicity, % |  |  |  |
| White | 472614 (94.1) | 62637 (92.1) | 409977 (94.4) |
| Nonwhite | 27021 (5.4) | 4559 (6.7) | 22462 (5.2) |
| Missing | 2776 (0.6) | 798 (1.2) | 1978 (0.5) |
| Household income, % |  |  |  |
| Less than 18,000 | 97182 (19.3) | 17282 (25.4) | 79900 (18.4) |
| 18,000 to 51,999 | 218910 (43.6) | 25827 (38.0) | 193083 (44.4) |
| Greater than 52,000 | 109177 (21.7) | 9627 (14.2) | 99550 (22.9) |
| Missing | 77142 (15.4) | 15258 (22.4) | 61884 (14.2) |
| Education, % |  |  |  |
| College education | 161130 (32.1) | 16782 (24.7) | 144348 (33.2) |
| Any school degree | 187370 (37.3) | 21753 (32.0) | 165617 (38.1) |
| Vocational qualifications | 32724 (6.5) | 4776 (7.0) | 27948 (6.4) |
| Other | 111058 (22.1) | 19456 (28.6) | 91602 (21.1) |
| Missing | 10129 (2.0) | 5227 (7.7) | 4902 (1.1) |
| Townsend deprivation index, mean (SD) | -1.29 (3.09) | -0.88 (3.29) | -1.36 (3.06) |
| ***Lifestyle behaviors*** |  |  |  |
| Alcohol consumption, % |  |  |  |
| Never | 51504 (10.3) | 9430 (13.9) | 42074 (9.7) |
| Low to moderate | 270119 (53.8) | 34677 (51.0) | 235442 (54.2) |
| Heavy | 106270 (21.2) | 12943 (19.0) | 93327 (21.5) |
| Missing | 74518 (14.8) | 10944 (16.1) | 63574 (14.6) |
| Smoking status, % |  |  |  |
| Never | 273476 (54.4) | 32512 (47.8) | 240964 (55.5) |
| Previous | 173025 (34.4) | 26721 (39.3) | 146304 (33.7) |
| Current | 52962 (10.5) | 7865 (11.6) | 45097 (10.4) |
| Missing | 2948 (0.6) | 896 (1.3) | 2052 (0.5) |
| Diet score, % |  |  |  |
| 0-1 | 57754 (11.5) | 8027 (11.8) | 49727 (11.4) |
| 2-3 | 233733 (46.5) | 31174 (45.8) | 202559 (46.6) |
| 4-5 | 188457 (37.5) | 24444 (36.0) | 164013 (37.8) |
| Missing | 22467 (4.5) | 4349 (6.4) | 18118 (4.2) |
| Physical active, % |  |  |  |
| Low | 76194 (15.2) | 11477 (16.9) | 64717 (14.9) |
| Medium | 164000 (32.6) | 20208 (29.7) | 143792 (33.1) |
| High | 162112 (32.3) | 18494 (27.2) | 143618 (33.1) |
| Missing | 100105 (19.9) | 17815 (26.2) | 82290 (18.9) |
| ***Clinical characteristics*** |  |  |  |
| FLI, mean (SD) | 48.06 (30.09) | 62.94 (27.88) | 46.95 (29.95) |
| Waist circumference (cm), mean (SD) | 90.31 (13.49) | 94.05 (14.21) | 89.74 (13.28) |
| BMI (kg/m^2^), mean (SD) | 27.43 (4.80) | 28.46 (5.18) | 27.28 (4.73) |
| Hypertension, % | 354423 (70.5) | 53528 (78.7) | 300895 (69.3) |
| Pre-diabetes, % | 68380 (14.7) | 11903 (21.9) | 56477 (13.7) |
| Diabetes, % | 30108 (6.0) | 8432 (12.4) | 21676 (5.0) |
| ***Laboratory measurements*** |  |  |  |
| TG (mmol/L), mean (SD) | 1.75 (1.03) | 1.94 (1.11) | 1.73 (1.02) |
| GGT (U/L), mean (SD) | 37.39 (42.09) | 50.06 (62.86) | 36.37 (39.78) |
| HbA1c (mmol/mol), mean (SD) | 36.13 (6.78) | 38.43 (9.27) | 35.83 (6.31) |
| HDL-cholesterol (mmol/L), mean (SD) | 1.45 (0.38) | 1.28 (0.36) | 1.46 (0.38) |
| CRP (mg/L), mean (SD) | 2.60 (4.36) | 3.47 (5.70) | 2.53 (4.23) |

Values are mean SD or n (%). **Abbreviation:** MAFLD, metabolic-associated fatty liver disease; FLI, fatty liver index; BMI, body mass index; TG, triglyceride; GGT, gamma-glutamyltransferase; HbA1c, glycated hemoglobin; HDL: high density lipoprotein; CRP: C-reactive protein.

**Supplemental Table 4.** **Associations of MAFLD subtypes and air pollutants exposure with intrahepatic and extrahepatic morbidity and all-cause mortality**

|  | **HR (95% CI)** | | | | |
| --- | --- | --- | --- | --- | --- |
|  | **Overall** | **Non-MAFLD** | **MAFLD-diabetes** | **MAFLD-lean** | **MAFLD-overweight/obesity** |
| **No. of participants, n** | 434417 | 274817 | 15325 | 3341 | 140934 |
| **Cirrhosis** |  |  |  |  |  |
| MAFLD | - | Ref | **4.46 (4.18, 4.76)** | **2.97 (2.56, 3.44)** | **2.40 (2.31, 2.50)** |
| Air pollutants (per IQR increase) |  |  |  |  |  |
| PM_2.5_ | **1.15 (1.12, 1.19)** | **1.19 (1.13, 1.25)** | **1.18 (1.07, 1.29)** | **1.43 (1.13, 1.82)** | **1.13 (1.09, 1.18)** |
| PM_10_ | **1.18 (1.15, 1.22)** | **1.24 (1.18, 1.29)** | **1.17 (1.07, 1.28)** | **1.43 (1.14, 1.79)** | **1.15 (1.11, 1.20)** |
| NO_2_ | **1.13 (1.10, 1.16)** | **1.15 (1.10, 1.20)** | **1.17 (1.08, 1.27)** | **1.22 (1.01, 1.47)** | **1.12 (1.08, 1.17)** |
| NO_x_ | **1.12 (1.10, 1.15)** | **1.15 (1.10, 1.19)** | **1.16 (1.08, 1.25)** | **1.20 (1.03, 1.40)** | **1.12 (1.08, 1.16)** |
| **Liver cancer** |  |  |  |  |  |
| MAFLD | - | Ref | **4.67 (3.83, 5.70)** | 1.78 (0.99, 3.20) | **1.50 (1.30, 1.74)** |
| Air pollutants (per IQR increase) |  |  |  |  |  |
| PM_2.5_ | 1.07 (0.96, 1.19) | 1.15 (0.97, 1.36) | 1.02 (0.75, 1.38) | 2.02 (0.61, 6.65) | 1.01 (0.86, 1.19) |
| PM_10_ | 1.06 (0.95, 1.17) | **1.18 (1.01, 1.39)** | 0.93 (0.69, 1.25) | 1.83 (0.60, 5.64) | 0.97 (0.84, 1.14) |
| NO_2_ | 1.06 (0.97, 1.17) | 1.12 (0.95, 1.31) | 1.25 (0.99, 1.58) | 1.85 (0.93, 3.67) | 0.96 (0.83, 1.11) |
| NO_x_ | 1.06 (0.97, 1.16) | 1.12 (0.97, 1.29) | **1.23 (1.00, 1.52)** | **1.74 (1.10, 2.75)** | 0.96 (0.83, 1.10) |
| **CAD** |  |  |  |  |  |
| MAFLD | - | Ref | **2.23 (2.13, 2.33)** | **1.33 (****1.19, 1.48)** | **1.45 (1.41, 1.48)** |
| Air pollutants (per IQR increase) |  |  |  |  |  |
| PM_2.5_ | 1.01 (0.99, 1.02) | 0.98 (0.96, 1.01) | **1.07 (1.00, 1.14)** | 1.15 (0.98, 1.34) | 1.02 (1.00, 1.05) |
| PM_10_ | **1.03 (1.01, 1.05)** | 1.01 (0.99, 1.04) | **1.08 (1.02, 1.15)** | 1.12 (0.96, 1.30) | **1.05 (1.02, 1.07)** |
| NO_2_ | 0.99 (0.97, 1.01) | 0.98 (0.95, 1.00) | 1.04 (0.98, 1.11) | 1.10 (0.95, 1.28) | 1.00 (0.98, 1.03) |
| NO_x_ | 0.99 (0.97, 1.01) | 0.98 (0.95, 1.00) | 1.03 (0.98, 1.09) | 1.08 (0.94, 1.25) | 1.00 (0.98, 1.03) |
| **IS** |  |  |  |  |  |
| MAFLD | - | Ref | **2.07 (1.89, 2.27)** | 1.21 (0.96, 1.53) | **1.22 (1.16, 1.29)** |
| Air pollutants (per IQR increase) |  |  |  |  |  |
| PM_2.5_ | 0.96 (0.92, 1.00) | 0.96 (0.91, 1.02) | 0.98 (0.86, 1.13) | 1.18 (0.82, 1.70) | 0.94 (0.88, 1.00) |
| PM_10_ | 0.95 (0.91, 1.00) | 0.96 (0.91, 1.01) | 0.96 (0.84, 1.10) | 1.13 (0.79, 1.63) | 0.93 (0.87, 1.00) |
| NO_2_ | 1.01 (0.97, 1.04) | 1.02 (0.97, 1.07) | 1.07 (0.94, 1.21) | 0.98 (0.67, 1.42) | 0.98 (0.93, 1.04) |
| NO_x_ | 1.00 (0.97, 1.04) | 1.01 (0.97, 1.06) | 1.05 (0.93, 1.19) | 0.98 (0.68, 1.40) | 0.98 (0.93, 1.03) |
| **PAD** |  |  |  |  |  |
| MAFLD | - | Ref | **2.77 (2.53, 3.03)** | **1.51 (****1.22, 1.88)** | **1.11 (1.04, 1.18)** |
| Air pollutants (per IQR increase) |  |  |  |  |  |
| PM_2.5_ | 1.04 (0.99, 1.09) | 1.06 (0.99, 1.13) | 0.91 (0.65, 1.26) | 1.08 (0.96, 1.23) | 1.01 (0.93, 1.08) |
| PM_10_ | **1.07 (1.02, 1.11)** | **1.09 (1.02, 1.15)** | 1.00 (0.72, 1.38) | 1.10 (0.98, 1.24) | 1.04 (0.97, 1.11) |
| NO_2_ | **1.05 (1.01, 1.09)** | **1.07 (1.01, 1.13)** | 0.99 (0.73, 1.34) | 1.08 (0.96, 1.22) | 1.01 (0.95, 1.09) |
| NO_x_ | **1.04 (1.00, 1.08)** | **1.06 (1.00, 1.12)** | 0.98 (0.73, 1.31) | 1.07 (0.96, 1.19) | 1.02 (0.95, 1.08) |
| **CKD** |  |  |  |  |  |
| MAFLD | - | Ref | **3.63 (3.45, 3.83)** | 1.17 (0.98, 1.40) | **1.56 (1.50, 1.61)** |
| Air pollutants (per IQR increase) |  |  |  |  |  |
| PM_2.5_ | **1.12 (1.09, 1.15)** | **1.10 (1.06, 1.14)** | **1.19 (1.11, 1.28)** | 1.13 (0.81, 1.59) | **1.13 (1.09, 1.17)** |
| PM_10_ | **1.15 (1.12, 1.18)** | **1.13 (1.09, 1.17)** | **1.20 (1.12, 1.29)** | 1.17 (0.84, 1.64) | **1.16 (1.12, 1.20)** |
| NO_2_ | **1.02 (1.00, 1.05)** | 1.01 (0.97, 1.04) | **1.14 (1.06, 1.22)** | 1.14 (0.84, 1.56) | 1.02 (0.99, 1.06) |
| NO_x_ | **1.03 (1.00, 1.05)** | 1.01 (0.98, 1.04) | **1.13 (1.06, 1.20)** | 1.13 (0.85, 1.51) | 1.03 (0.99, 1.06) |
| **All-cause mortality** |  |  |  |  |  |
| MAFLD | - | Ref | **1.81 (1.74, 1.90)** | **1.45 (****1.32, 1.60)** | **1.13 (1.10, 1.16)** |
| Air pollutants (per IQR increase) |  |  |  |  |  |
| PM_2.5_ | 1.00 (0.98, 1.01) | 0.99 (0.97, 1.02) | 1.01 (0.94, 1.08) | 1.01 (0.86, 1.19) | 1.00 (0.97, 1.03) |
| PM_10_ | 1.02 (1.00, 1.03) | 1.02 (0.99, 1.04) | 1.04 (0.97, 1.10) | 1.05 (0.90, 1.23) | 1.01 (0.99, 1.04) |
| NO_2_ | 1.00 (0.99, 1.02) | 1.01 (0.99, 1.04) | 1.04 (0.97, 1.11) | 0.98 (0.85, 1.14) | 0.99 (0.96, 1.02) |
| NO_x_ | 1.01 (0.99, 1.02) | 1.01 (0.99, 1.04) | 1.05 (0.99, 1.12) | 0.99 (0.86, 1.13) | 0.99 (0.97, 1.02) |

The hazard ratios and 95% confidence intervals were calculated using the time-varying Cox proportional hazard regression models adjusted for age, sex, ethnicity, alcohol consumption, smoking status, diet score, physical activity, annual household income, education, and Townsend Deprivation Index.

**Abbreviation:** MAFLD, metabolic-associated fatty liver disease; PM_2.5_, fine particulate matter with diameter <2.5 μm; PM_10_, particulate matter with diameter <10 μm; NO_2_, nitrogen dioxide; NO_x_, nitrogen oxides; CAD, coronary artery disease; IS, ischemic stroke; PAD, peripheral artery disease; CKD, chronic kidney disease.

**Supplemental Table 5. Hazard ratios (95% CIs) for the intrahepatic and extrahepatic morbidity and all-cause mortality associated with the four air pollutants among participants with different subtypes of MAFLD in UK Biobank**

| **Air pollution** | **Non-MAFLD** | | **MAFLD-diabetes** | | **MAFLD-lean** | | **MAFLD-overweight/obesity** | |
| --- | --- | --- | --- | --- | --- | --- | --- | --- |
|  | **HR (95% CI)** | ***p* for trend** | **HR (95% CI)** | ***p* for trend** | **HR (95% CI)** | ***p* for trend** | **HR (95% CI)** | ***p* for trend** |
| **Cirrhosis** |  |  |  |  |  |  |  |  |
| PM_2.5_ |  |  |  |  |  |  |  |  |
| Low | Ref | <0.001 | 3.84 (3.47, 4.25) | 0.042 | 2.20 (1.71, 2.84) | 0.007 | 2.19 (2.07, 2.32) | 0.003 |
| Intermediate | 0.98 (0.91, 1.05) |  | 4.53 (4.06, 5.05) |  | 2.92 (2.23, 3.83) |  | 2.51 (2.35, 2.68) |  |
| High | 1.02 (0.95, 1.10) |  | 5.41 (4.85, 6.03) |  | 4.32 (3.39, 5.51) |  | 2.67 (2.48, 2.86) |  |
| PM_10_ |  |  |  |  |  |  |  |  |
| Low | Ref | <0.001 | 4.16 (3.76, 4.60) | 0.082 | 2.59 (2.02, 3.31) | 0.018 | 2.33 (2.20, 2.48) | 0.004 |
| Intermediate | 1.05 (0.98, 1.12) |  | 4.64 (4.16, 5.16) |  | 2.46 (1.85, 3.27) |  | 2.53 (2.37, 2.69) |  |
| High | 1.12 (1.04, 1.21) |  | 5.54 (4.96, 6.18) |  | 4.86 (3.83, 6.17) |  | 2.83 (2.64, 3.04) |  |
| NO_2_ |  |  |  |  |  |  |  |  |
| Low | Ref | <0.001 | 3.97 (3.57, 4.41) | 0.012 | 2.48 (1.92, 3.19) | 0.122 | 2.21 (2.08, 2.35) | <0.001 |
| Intermediate | 0.95 (0.89, 1.02) |  | 4.41 (3.95, 4.92) |  | 2.42 (1.82, 3.22) |  | 2.45 (2.30, 2.61) |  |
| High | 1.09 (1.01, 1.17) |  | 5.33 (4.80, 5.93) |  | 4.42 (3.50, 5.58) |  | 2.73 (2.55, 2.93) |  |
| NO_x_ |  |  |  |  |  |  |  |  |
| Low | Ref | <0.001 | 4.00 (3.60, 4.44) | 0.010 | 2.46 (1.91, 3.17) | 0.076 | 2.21 (2.08, 2.34) | <0.001 |
| Intermediate | 0.94 (0.88, 1.01) |  | 4.29 (3.85, 4.79) |  | 2.38 (1.79, 3.18) |  | 2.44 (2.29, 2.60) |  |
| High | 1.09 (1.01, 1.17) |  | 5.39 (4.85, 5.99) |  | 4.47 (3.54, 5.63) |  | 2.73 (2.55, 2.93) |  |
| **Liver cancer** |  |  |  |  |  |  |  |  |
| PM_2.5_ |  |  |  |  |  |  |  |  |
| Low | Ref | 0.204 | 5.96 (4.43, 8.01) | 0.895 | 2.44 (1.07, 5.57) | 0.899 | 1.77 (1.42, 2.21) | 0.872 |
| Intermediate | 1.28 (1.01, 1.63) |  | 5.29 (3.73, 7.51) |  | 1.08 (0.26, 4.38) |  | 1.58 (1.22, 2.06) |  |
| High | 1.26 (0.96, 1.65) |  | 4.66 (3.12, 6.96) |  | 2.64 (0.97, 7.20) |  | 1.84 (1.39, 2.43) |  |
| PM_10_ |  |  |  |  |  |  |  |  |
| Low | Ref | 0.038 | 6.82 (5.09, 9.13) | 0.819 | 2.62 (1.14, 5.98) | 0.740 | 1.81 (1.44, 2.27) | 0.938 |
| Intermediate | 1.34 (1.06, 1.70) |  | 4.48 (3.10, 6.46) |  | 0.52 (0.07, 3.72) |  | 1.71 (1.33, 2.20) |  |
| High | 1.36 (1.04, 1.78) |  | 5.23 (3.53, 7.75) |  | 3.62 (1.47, 8.92) |  | 1.92 (1.46, 2.53) |  |
| NO_2_ |  |  |  |  |  |  |  |  |
| Low | Ref | 0.495 | 4.80 (3.47, 6.64) | 0.183 | 2.47 (1.09, 5.64) | 0.364 | 1.59 (1.26, 1.99) | 0.715 |
| Intermediate | 1.13 (0.89, 1.43) |  | 5.95 (4.32, 8.19) |  | 0.96 (0.23, 3.89) |  | 1.70 (1.33, 2.17) |  |
| High | 1.17 (0.89, 1.54) |  | 4.49 (3.07, 6.57) |  | 2.40 (0.88, 6.53) |  | 1.61 (1.22, 2.13) |  |
| NO_x_ |  |  |  |  |  |  |  |  |
| Low | Ref | 0.352 | 4.83 (3.49, 6.68) | 0.262 | 2.49 (1.09, 5.68) | 0.317 | 1.60 (1.27, 2.01) | 0.627 |
| Intermediate | 1.12 (0.89, 1.43) |  | 6.12 (4.45, 8.41) |  | 0.97 (0.24, 3.93) |  | 1.73 (1.36, 2.21) |  |
| High | 1.21 (0.92, 1.59) |  | 4.40 (3.00, 6.47) |  | 2.41 (0.89, 6.58) |  | 1.61 (1.22, 2.12) |  |
| **CAD** |  |  |  |  |  |  |  |  |
| PM_2.5_ |  |  |  |  |  |  |  |  |
| Low | Ref | 0.041 | 1.94 (1.81, 2.09) | 0.057 | 1.11 (0.93, 1.33) | 0.132 | 1.38 (1.34, 1.43) | 0.541 |
| Intermediate | 0.97 (0.93, 1.01) |  | 2.39 (2.22, 2.56) |  | 1.44 (1.21, 1.73) |  | 1.42 (1.36, 1.48) |  |
| High | 0.91 (0.87, 0.95) |  | 2.22 (2.06, 2.40) |  | 1.41 (1.16, 1.71) |  | 1.42 (1.35, 1.48) |  |
| PM_10_ |  |  |  |  |  |  |  |  |
| Low | Ref | 0.408 | 2.01 (1.87, 2.16) | 0.042 | 1.08 (0.89, 1.30) | 0.058 | 1.38 (1.33, 1.43) | 0.357 |
| Intermediate | 0.98 (0.95, 1.02) |  | 2.25 (2.09, 2.41) |  | 1.48 (1.25, 1.75) |  | 1.44 (1.39, 1.50) |  |
| High | 0.94 (0.90, 0.98) |  | 2.36 (2.18, 2.55) |  | 1.45 (1.19, 1.77) |  | 1.46 (1.40, 1.53) |  |
| NO_2_ |  |  |  |  |  |  |  |  |
| Low | Ref | 0.654 | 1.98 (1.85, 2.13) | 0.008 | 1.22 (1.02, 1.46) | 0.441 | 1.40 (1.35, 1.45) | 0.873 |
| Intermediate | 0.95 (0.92, 0.99) |  | 2.23 (2.07, 2.40) |  | 1.28 (1.06, 1.54) |  | 1.38 (1.33, 1.44) |  |
| High | 0.96 (0.92, 1.00) |  | 2.34 (2.16, 2.52) |  | 1.43 (1.18, 1.74) |  | 1.46 (1.39, 1.52) |  |
| NO_x_ |  |  |  |  |  |  |  |  |
| Low | Ref | 0.902 | 1.99 (1.86, 2.14) | 0.010 | 1.23 (1.03, 1.47) | 0.487 | 1.41 (1.36, 1.46) | 0.856 |
| Intermediate | 0.96 (0.92, 1.00) |  | 2.23 (2.08, 2.40) |  | 1.29 (1.07, 1.55) |  | 1.39 (1.34, 1.45) |  |
| High | 0.96 (0.92, 1.00) |  | 2.35 (2.17, 2.53) |  | 1.42 (1.17, 1.73) |  | 1.46 (1.40, 1.53) |  |
| **IS** |  |  |  |  |  |  |  |  |
| PM_2.5_ |  |  |  |  |  |  |  |  |
| Low | Ref | 0.203 | 2.03 (1.76, 2.35) | 0.852 | 1.14 (0.78, 1.67) | 0.271 | 1.21 (1.12, 1.31) | 0.210 |
| Intermediate | 0.98 (0.91, 1.07) |  | 2.07 (1.78, 2.42) |  | 1.09 (0.72, 1.67) |  | 1.24 (1.13, 1.35) |  |
| High | 0.94 (0.86, 1.04) |  | 1.96 (1.66, 2.32) |  | 1.34 (0.88, 2.03) |  | 1.12 (1.01, 1.24) |  |
| PM_10_ |  |  |  |  |  |  |  |  |
| Low | Ref | 0.162 | 1.98 (1.71, 2.29) | 0.711 | 1.05 (0.71, 1.56) | 0.228 | 1.23 (1.13, 1.33) | 0.132 |
| Intermediate | 0.99 (0.91, 1.07) |  | 2.06 (1.76, 2.39) |  | 1.20 (0.80, 1.78) |  | 1.20 (1.10, 1.31) |  |
| High | 0.93 (0.85, 1.02) |  | 2.03 (1.72, 2.40) |  | 1.34 (0.88, 2.05) |  | 1.13 (1.02, 1.25) |  |
| NO_2_ |  |  |  |  |  |  |  |  |
| Low | Ref | 0.789 | 1.90 (1.62, 2.22) | 0.385 | 1.36 (0.94, 1.95) | 0.380 | 1.24 (1.14, 1.35) | 0.170 |
| Intermediate | 0.97 (0.89, 1.05) |  | 2.18 (1.87, 2.54) |  | 1.07 (0.70, 1.63) |  | 1.22 (1.11, 1.33) |  |
| High | 1.01 (0.93, 1.11) |  | 2.09 (1.78, 2.44) |  | 1.15 (0.74, 1.79) |  | 1.16 (1.05, 1.29) |  |
| NO_x_ |  |  |  |  |  |  |  |  |
| Low | Ref | 0.706 | 1.88 (1.60, 2.20) | 0.293 | 1.36 (0.95, 1.96) | 0.504 | 1.25 (1.15, 1.36) | 0.123 |
| Intermediate | 0.98 (0.90, 1.06) |  | 2.22 (1.91, 2.58) |  | 1.03 (0.67, 1.58) |  | 1.23 (1.12, 1.34) |  |
| High | 1.02 (0.93, 1.11) |  | 2.10 (1.79, 2.46) |  | 1.21 (0.78, 1.87) |  | 1.16 (1.05, 1.28) |  |
| **PAD** |  |  |  |  |  |  |  |  |
| PM_2.5_ |  |  |  |  |  |  |  |  |
| Low | Ref | 0.755 | 2.48 (2.16, 2.85) | 0.100 | 1.51 (1.10, 2.08) | 0.365 | 1.14 (1.05, 1.25) | 0.345 |
| Intermediate | 1.05 (0.95, 1.15) |  | 3.30 (2.86, 3.82) |  | 2.10 (1.50, 2.95) |  | 1.12 (1.01, 1.24) |  |
| High | 1.01 (0.91, 1.13) |  | 2.85 (2.40, 3.38) |  | 0.85 (0.46, 1.54) |  | 1.08 (0.96, 1.22) |  |
| PM_10_ |  |  |  |  |  |  |  |  |
| Low | Ref | 0.699 | 2.75 (2.40, 3.16) | 0.835 | 1.54 (1.11, 2.13) | 0.863 | 1.15 (1.05, 1.25) | 0.736 |
| Intermediate | 1.06 (0.97, 1.17) |  | 2.91 (2.51, 3.37) |  | 1.80 (1.27, 2.55) |  | 1.13 (1.02, 1.25) |  |
| High | 1.02 (0.91, 1.13) |  | 2.89 (2.44, 3.43) |  | 1.19 (0.72, 1.99) |  | 1.10 (0.98, 1.25) |  |
| NO_2_ |  |  |  |  |  |  |  |  |
| Low | Ref | 0.573 | 2.57 (2.22, 2.98) | 0.232 | 1.26 (0.87, 1.84) | 0.814 | 1.14 (1.04, 1.24) | 0.600 |
| Intermediate | 0.96 (0.87, 1.05) |  | 3.00 (2.60, 3.47) |  | 1.99 (1.43, 2.76) |  | 1.12 (1.01, 1.24) |  |
| High | 1.08 (0.97, 1.20) |  | 2.85 (2.42, 3.35) |  | 1.32 (0.85, 2.07) |  | 1.07 (0.95, 1.20) |  |
| NO_x_ |  |  |  |  |  |  |  |  |
| Low | Ref | 0.286 | 2.60 (2.25, 3.00) | 0.206 | 1.31 (0.91, 1.89) | 0.865 | 1.13 (1.03, 1.24) | 0.813 |
| Intermediate | 0.97 (0.88, 1.06) |  | 2.99 (2.58, 3.45) |  | 2.02 (1.46, 2.80) |  | 1.14 (1.03, 1.26) |  |
| High | 1.10 (1.00, 1.22) |  | 2.93 (2.49, 3.44) |  | 1.27 (0.80, 2.01) |  | 1.09 (0.97, 1.22) |  |
| **CKD** |  |  |  |  |  |  |  |  |
| PM_2.5_ |  |  |  |  |  |  |  |  |
| Low | Ref | <0.001 | 3.57 (3.29, 3.88) | <0.001 | 1.10 (0.83, 1.45) | 0.549 | 1.58 (1.51, 1.67) | <0.001 |
| Intermediate | 1.19 (1.13, 1.26) |  | 4.47 (4.11, 4.86) |  | 1.73 (1.31, 2.29) |  | 1.75 (1.66, 1.86) |  |
| High | 1.20 (1.13, 1.27) |  | 4.29 (3.91, 4.72) |  | 1.11 (0.75, 1.65) |  | 1.94 (1.82, 2.07) |  |
| PM_10_ |  |  |  |  |  |  |  |  |
| Low | Ref | <0.001 | 3.62 (3.33, 3.94) | <0.001 | 1.03 (0.77, 1.39) | 0.120 | 1.54 (1.46, 1.62) | <0.001 |
| Intermediate | 1.12 (1.06, 1.18) |  | 4.16 (3.82, 4.53) |  | 1.54 (1.16, 2.03) |  | 1.75 (1.66, 1.85) |  |
| High | 1.20 (1.13, 1.28) |  | 4.26 (3.88, 4.68) |  | 1.30 (0.90, 1.87) |  | 1.90 (1.78, 2.02) |  |
| NO_2_ |  |  |  |  |  |  |  |  |
| Low | Ref | 0.021 | 3.59 (3.31, 3.90) | <0.001 | 1.17 (0.89, 1.54) | 0.557 | 1.58 (1.50, 1.66) | 0.024 |
| Intermediate | 1.10 (1.05, 1.16) |  | 3.90 (3.56, 4.28) |  | 1.23 (0.90, 1.68) |  | 1.68 (1.59, 1.77) |  |
| High | 1.09 (1.03, 1.16) |  | 4.15 (3.80, 4.54) |  | 1.36 (0.97, 1.92) |  | 1.72 (1.61, 1.83) |  |
| NO_x_ |  |  |  |  |  |  |  |  |
| Low | Ref | 0.014 | 3.61 (3.33, 3.91) | <0.001 | 1.17 (0.89, 1.54) | 0.374 | 1.57 (1.49, 1.65) | 0.009 |
| Intermediate | 1.09 (1.04, 1.15) |  | 3.85 (3.51, 4.22) |  | 1.17 (0.85, 1.62) |  | 1.67 (1.58, 1.77) |  |
| High | 1.10 (1.04, 1.17) |  | 4.18 (3.83, 4.58) |  | 1.44 (1.03, 2.02) |  | 1.73 (1.63, 1.85) |  |
| **All-cause mortality** |  |  |  |  |  |  |  |  |
| PM_2.5_ |  |  |  |  |  |  |  |  |
| Low | Ref | 0.566 | 1.88 (1.77, 2.01) | 0.94 | 1.41 (1.23, 1.62) | 0.29 | 1.15 (1.11, 1.19) | 0.919 |
| Intermediate | 1.01 (0.97, 1.05) |  | 1.76 (1.63, 1.90) |  | 1.28 (1.07, 1.54) |  | 1.13 (1.08, 1.18) |  |
| High | 1.01 (0.97, 1.06) |  | 1.78 (1.63, 1.94) |  | 1.77 (1.48, 2.13) |  | 1.12 (1.07, 1.18) |  |
| PM_10_ |  |  |  |  |  |  |  |  |
| Low | Ref | 0.676 | 1.92 (1.80, 2.04) | 0.879 | 1.41 (1.22, 1.63) | 0.304 | 1.14 (1.10, 1.18) | 0.651 |
| Intermediate | 0.97 (0.94, 1.01) |  | 1.64 (1.51, 1.78) |  | 1.30 (1.09, 1.55) |  | 1.10 (1.05, 1.14) |  |
| High | 1.02 (0.98, 1.06) |  | 1.83 (1.68, 1.99) |  | 1.73 (1.44, 2.08) |  | 1.14 (1.09, 1.20) |  |
| NO_2_ |  |  |  |  |  |  |  |  |
| Low | Ref | 0.37 | 1.86 (1.74, 1.99) | 0.709 | 1.36 (1.17, 1.57) | 0.829 | 1.15 (1.11, 1.19) | 0.514 |
| Intermediate | 0.98 (0.95, 1.02) |  | 1.81 (1.67, 1.95) |  | 1.52 (1.29, 1.79) |  | 1.10 (1.05, 1.14) |  |
| High | 1.05 (1.00, 1.09) |  | 1.79 (1.65, 1.94) |  | 1.55 (1.29, 1.87) |  | 1.16 (1.10, 1.21) |  |
| NO_x_ |  |  |  |  |  |  |  |  |
| Low | Ref | 0.222 | 1.87 (1.76, 2.00) | 0.708 | 1.36 (1.17, 1.58) | 0.883 | 1.15 (1.11, 1.20) | 0.570 |
| Intermediate | 0.98 (0.94, 1.01) |  | 1.78 (1.65, 1.93) |  | 1.50 (1.27, 1.77) |  | 1.10 (1.05, 1.14) |  |
| High | 1.05 (1.01, 1.10) |  | 1.80 (1.66, 1.95) |  | 1.57 (1.30, 1.89) |  | 1.16 (1.10, 1.21) |  |

The hazard ratios and 95% confidence intervals were calculated using the time-varying Cox proportional hazard regression models adjusted for age, sex, ethnicity, alcohol consumption, smoking status, diet score, physical activity, annual household income, education, and Townsend Deprivation Index.

**Abbreviation:** MAFLD, metabolic-associated fatty liver disease; PM_2.5_, fine particulate matter with diameter <2.5 μm; PM_10_, particulate matter with diameter <10 μm; NO_2_, nitrogen dioxide; NO_x_, nitrogen oxides; CAD, coronary artery disease; IS, ischemic stroke; PAD, peripheral artery disease; CKD, chronic kidney disease.

**Supplemental Table 6. Hazard ratios (95% CIs) for the intrahepatic and extrahepatic morbidity and all-cause mortality associated with the four air pollutants among participants with different subtypes of MAFLD** **after excluding events within the first two years of follow-up**

| **Air pollution** | **MAFLD subtype** | | | |
| --- | --- | --- | --- | --- |
|  | **Non-MAFLD** | **MAFLD-diabetes** | **MAFLD-lean** | **MAFLD-overweight/obesity** |
| **Cirrhosis** |  |  |  |  |
| PM_2.5_ |  |  |  |  |
| Low | Ref | 3.76 (3.38, 4.17) | 2.17 (1.67, 2.81) | 2.15 (2.03, 2.28) |
| Intermediate | 0.99 (0.93, 1.07) | 4.63 (4.13, 5.19) | 2.87 (2.16, 3.82) | 2.46 (2.30, 2.63) |
| High | 1.05 (0.97, 1.14) | 5.17 (4.59, 5.83) | 4.17 (3.20, 5.44) | 2.61 (2.42, 2.82) |
| PM_10_ |  |  |  |  |
| Low | Ref | 4.14 (3.74, 4.60) | 2.58 (2.01, 3.31) | 2.30 (2.16, 2.44) |
| Intermediate | 1.06 (0.99, 1.13) | 4.65 (4.16, 5.21) | 2.43 (1.81, 3.27) | 2.46 (2.30, 2.63) |
| High | 1.19 (1.10, 1.28) | 5.32 (4.71, 6.01) | 4.74 (3.63, 6.17) | 2.81 (2.61, 3.03) |
| NO_2_ |  |  |  |  |
| Low | Ref | 3.95 (3.54, 4.40) | 2.42 (1.86, 3.13) | 2.16 (2.03, 2.30) |
| Intermediate | 0.95 (0.89, 1.02) | 4.27 (3.81, 4.79) | 2.46 (1.83, 3.30) | 2.39 (2.23, 2.55) |
| High | 1.11 (1.03, 1.20) | 5.18 (4.62, 5.81) | 4.14 (3.20, 5.36) | 2.65 (2.47, 2.86) |
| NO_x_ |  |  |  |  |
| Low | Ref | 3.98 (3.57, 4.43) | 2.40 (1.85, 3.11) | 2.16 (2.03, 2.29) |
| Intermediate | 0.95 (0.88, 1.01) | 4.17 (3.72, 4.69) | 2.41 (1.79, 3.24) | 2.37 (2.22, 2.53) |
| High | 1.11 (1.02, 1.19) | 5.19 (4.63, 5.82) | 4.18 (3.23, 5.40) | 2.64 (2.45, 2.85) |
| **Liver cancer** |  |  |  |  |
| PM_2.5_ |  |  |  |  |
| Low | Ref | 5.67 (4.17, 7.70) | 2.46 (1.08, 5.62) | 1.75 (1.40, 2.19) |
| Intermediate | 1.23 (0.96, 1.58) | 5.34 (3.72, 7.65) | 0.57 (0.08, 4.09) | 1.43 (1.08, 1.89) |
| High | 1.15 (0.86, 1.55) | 4.65 (3.04, 7.10) | 2.20 (0.69, 6.97) | 1.67 (1.23, 2.25) |
| PM_10_ |  |  |  |  |
| Low | Ref | 6.59 (4.89, 8.89) | 2.62 (1.15, 6.00) | 1.77 (1.41, 2.23) |
| Intermediate | 1.30 (1.02, 1.66) | 4.55 (3.13, 6.63) | 0.54 (0.08, 3.90) | 1.62 (1.25, 2.11) |
| High | 1.31 (0.98, 1.75) | 5.27 (3.45, 8.04) | 2.61 (0.82, 8.27) | 1.76 (1.31, 2.38) |
| NO_2_ |  |  |  |  |
| Low | Ref | 4.85 (3.49, 6.73) | 2.52 (1.10, 5.74) | 1.57 (1.25, 1.98) |
| Intermediate | 1.09 (0.85, 1.40) | 5.82 (4.18, 8.11) | 1.01 (0.25, 4.11) | 1.63 (1.27, 2.10) |
| High | 1.11 (0.83, 1.48) | 4.40 (2.93, 6.60) | 1.36 (0.34, 5.52) | 1.42 (1.05, 1.92) |
| NO_x_ |  |  |  |  |
| Low | Ref | 4.88 (3.51, 6.78) | 2.54 (1.11, 5.79) | 1.58 (1.25, 1.99) |
| Intermediate | 1.08 (0.84, 1.39) | 6.00 (4.31, 8.34) | 1.02 (0.25, 4.14) | 1.65 (1.28, 2.12) |
| High | 1.15 (0.86, 1.54) | 4.29 (2.84, 6.48) | 1.37 (0.34, 5.55) | 1.43 (1.06, 1.94) |
| **CAD** |  |  |  |  |
| PM_2.5_ |  |  |  |  |
| Low | Ref | 1.95 (1.81, 2.10) | 1.12 (0.93, 1.35) | 1.39 (1.34, 1.44) |
| Intermediate | 0.98 (0.94, 1.02) | 2.42 (2.24, 2.62) | 1.43 (1.18, 1.74) | 1.43 (1.37, 1.50) |
| High | 0.92 (0.88, 0.96) | 2.28 (2.10, 2.49) | 1.48 (1.20, 1.83) | 1.42 (1.36, 1.50) |
| PM_10_ |  |  |  |  |
| Low | Ref | 2.02 (1.87, 2.17) | 1.06 (0.87, 1.30) | 1.39 (1.34, 1.44) |
| Intermediate | 0.99 (0.96, 1.03) | 2.31 (2.14, 2.49) | 1.52 (1.27, 1.81) | 1.45 (1.40, 1.51) |
| High | 0.95 (0.91, 1.00) | 2.38 (2.18, 2.60) | 1.51 (1.21, 1.89) | 1.47 (1.40, 1.54) |
| NO_2_ |  |  |  |  |
| Low | Ref | 2.00 (1.86, 2.15) | 1.20 (1.00, 1.45) | 1.41 (1.36, 1.47) |
| Intermediate | 0.96 (0.92, 1.00) | 2.28 (2.11, 2.46) | 1.31 (1.08, 1.59) | 1.39 (1.33, 1.44) |
| High | 0.95 (0.91, 1.00) | 2.32 (2.13, 2.53) | 1.45 (1.17, 1.80) | 1.44 (1.38, 1.52) |
| NO_x_ |  |  |  |  |
| Low | Ref | 2.00 (1.86, 2.15) | 1.22 (1.01, 1.47) | 1.41 (1.36, 1.47) |
| Intermediate | 0.96 (0.93, 1.00) | 2.29 (2.12, 2.47) | 1.31 (1.08, 1.59) | 1.39 (1.34, 1.45) |
| High | 0.96 (0.92, 1.00) | 2.32 (2.13, 2.53) | 1.44 (1.16, 1.78) | 1.44 (1.37, 1.51) |
| **IS** |  |  |  |  |
| PM_2.5_ |  |  |  |  |
| Low | Ref | 1.94 (1.66, 2.26) | 1.06 (0.70, 1.59) | 1.21 (1.11, 1.32) |
| Intermediate | 0.99 (0.91, 1.08) | 1.98 (1.67, 2.34) | 1.08 (0.68, 1.70) | 1.21 (1.10, 1.33) |
| High | 0.92 (0.83, 1.02) | 1.90 (1.58, 2.28) | 1.19 (0.73, 1.93) | 1.10 (0.98, 1.23) |
| PM_10_ |  |  |  |  |
| Low | Ref | 1.95 (1.68, 2.28) | 0.99 (0.65, 1.51) | 1.24 (1.14, 1.35) |
| Intermediate | 0.99 (0.91, 1.08) | 1.85 (1.56, 2.19) | 1.18 (0.77, 1.80) | 1.18 (1.08, 1.30) |
| High | 0.92 (0.83, 1.02) | 2.04 (1.70, 2.46) | 1.17 (0.70, 1.95) | 1.08 (0.97, 1.21) |
| NO_2_ |  |  |  |  |
| Low | Ref | 1.80 (1.52, 2.12) | 1.25 (0.85, 1.85) | 1.23 (1.13, 1.34) |
| Intermediate | 0.96 (0.88, 1.05) | 2.00 (1.69, 2.36) | 1.03 (0.66, 1.63) | 1.21 (1.10, 1.32) |
| High | 0.98 (0.89, 1.08) | 2.08 (1.75, 2.47) | 0.97 (0.57, 1.66) | 1.10 (0.99, 1.23) |
| NO_x_ |  |  |  |  |
| Low | Ref | 1.77 (1.50, 2.10) | 1.26 (0.86, 1.86) | 1.23 (1.13, 1.34) |
| Intermediate | 0.97 (0.89, 1.05) | 2.03 (1.72, 2.40) | 1.04 (0.66, 1.63) | 1.21 (1.10, 1.33) |
| High | 0.98 (0.89, 1.08) | 2.09 (1.76, 2.48) | 0.97 (0.57, 1.65) | 1.10 (0.99, 1.23) |
| **PAD** |  |  |  |  |
| PM_2.5_ |  |  |  |  |
| Low | Ref | 2.41 (2.08, 2.79) | 1.54 (1.11, 2.14) | 1.17 (1.07, 1.28) |
| Intermediate | 1.06 (0.97, 1.17) | 3.39 (2.91, 3.95) | 2.07 (1.44, 2.98) | 1.13 (1.01, 1.26) |
| High | 1.01 (0.90, 1.13) | 3.03 (2.52, 3.65) | 0.76 (0.38, 1.54) | 1.14 (1.00, 1.30) |
| PM_10_ |  |  |  |  |
| Low | Ref | 2.68 (2.32, 3.10) | 1.55 (1.11, 2.17) | 1.18 (1.07, 1.29) |
| Intermediate | 1.08 (0.98, 1.19) | 3.05 (2.62, 3.55) | 1.83 (1.27, 2.63) | 1.15 (1.04, 1.28) |
| High | 1.02 (0.91, 1.14) | 2.94 (2.43, 3.56) | 1.04 (0.56, 1.95) | 1.15 (1.01, 1.31) |
| NO_2_ |  |  |  |  |
| Low | Ref | 2.55 (2.19, 2.98) | 1.30 (0.89, 1.90) | 1.15 (1.05, 1.26) |
| Intermediate | 0.97 (0.88, 1.07) | 3.05 (2.62, 3.55) | 2.04 (1.45, 2.87) | 1.16 (1.05, 1.29) |
| High | 1.07 (0.96, 1.19) | 2.85 (2.39, 3.41) | 1.15 (0.67, 1.95) | 1.07 (0.94, 1.21) |
| NO_x_ |  |  |  |  |
| Low | Ref | 2.58 (2.21, 3.01) | 1.35 (0.93, 1.96) | 1.15 (1.04, 1.26) |
| Intermediate | 0.97 (0.88, 1.07) | 3.03 (2.60, 3.53) | 2.07 (1.47, 2.91) | 1.18 (1.07, 1.31) |
| High | 1.08 (0.97, 1.21) | 2.91 (2.43, 3.47) | 1.07 (0.62, 1.85) | 1.07 (0.94, 1.22) |
| **CKD** |  |  |  |  |
| PM_2.5_ |  |  |  |  |
| Low | Ref | 3.57 (3.28, 3.88) | 1.11 (0.84, 1.46) | 1.59 (1.51, 1.67) |
| Intermediate | 1.16 (1.10, 1.23) | 4.31 (3.95, 4.72) | 1.75 (1.32, 2.33) | 1.72 (1.62, 1.82) |
| High | 1.17 (1.09, 1.24) | 4.13 (3.73, 4.57) | 1.17 (0.78, 1.76) | 1.85 (1.73, 1.98) |
| PM_10_ |  |  |  |  |
| Low | Ref | 3.60 (3.30, 3.91) | 1.07 (0.80, 1.43) | 1.54 (1.46, 1.62) |
| Intermediate | 1.09 (1.03, 1.14) | 4.06 (3.72, 4.44) | 1.56 (1.18, 2.06) | 1.71 (1.62, 1.81) |
| High | 1.15 (1.08, 1.22) | 3.94 (3.56, 4.37) | 1.25 (0.84, 1.87) | 1.78 (1.67, 1.90) |
| NO_2_ |  |  |  |  |
| Low | Ref | 3.50 (3.22, 3.81) | 1.18 (0.90, 1.55) | 1.57 (1.49, 1.65) |
| Intermediate | 1.05 (1.00, 1.11) | 3.79 (3.44, 4.17) | 1.24 (0.90, 1.70) | 1.61 (1.52, 1.70) |
| High | 1.04 (0.97, 1.10) | 3.90 (3.54, 4.29) | 1.36 (0.95, 1.96) | 1.62 (1.51, 1.73) |
| NO_x_ |  |  |  |  |
| Low | Ref | 3.53 (3.25, 3.83) | 1.16 (0.88, 1.52) | 1.56 (1.48, 1.64) |
| Intermediate | 1.04 (0.99, 1.10) | 3.74 (3.39, 4.12) | 1.21 (0.88, 1.67) | 1.61 (1.52, 1.70) |
| High | 1.04 (0.97, 1.11) | 3.90 (3.54, 4.30) | 1.45 (1.02, 2.06) | 1.62 (1.51, 1.73) |
| **All-cause mortality** |  |  |  |  |
| PM_2.5_ |  |  |  |  |
| Low | Ref | 1.91 (1.79, 2.04) | 1.39 (1.20, 1.61) | 1.15 (1.11, 1.19) |
| Intermediate | 1.01 (0.97, 1.05) | 1.80 (1.66, 1.96) | 1.23 (1.01, 1.50) | 1.15 (1.10, 1.20) |
| High | 1.02 (0.97, 1.06) | 1.82 (1.67, 2.00) | 1.78 (1.47, 2.16) | 1.12 (1.06, 1.18) |
| PM_10_ |  |  |  |  |
| Low | Ref | 1.95 (1.83, 2.08) | 1.39 (1.20, 1.61) | 1.14 (1.10, 1.18) |
| Intermediate | 0.97 (0.94, 1.01) | 1.68 (1.54, 1.82) | 1.27 (1.06, 1.53) | 1.11 (1.06, 1.15) |
| High | 1.02 (0.98, 1.07) | 1.87 (1.71, 2.05) | 1.71 (1.40, 2.10) | 1.15 (1.09, 1.21) |
| NO_2_ |  |  |  |  |
| Low | Ref | 1.89 (1.76, 2.02) | 1.35 (1.16, 1.57) | 1.16 (1.12, 1.21) |
| Intermediate | 0.99 (0.95, 1.02) | 1.84 (1.69, 2.00) | 1.45 (1.22, 1.73) | 1.10 (1.06, 1.15) |
| High | 1.04 (0.99, 1.09) | 1.84 (1.69, 2.01) | 1.55 (1.26, 1.90) | 1.16 (1.10, 1.22) |
| NO_x_ |  |  |  |  |
| Low | Ref | 1.90 (1.77, 2.03) | 1.34 (1.15, 1.56) | 1.16 (1.12, 1.21) |
| Intermediate | 0.98 (0.94, 1.01) | 1.81 (1.66, 1.96) | 1.45 (1.21, 1.73) | 1.10 (1.05, 1.15) |
| High | 1.04 (1.00, 1.09) | 1.85 (1.70, 2.02) | 1.55 (1.27, 1.90) | 1.15 (1.10, 1.21) |

The hazard ratios and 95% confidence intervals were calculated using the time-varying Cox proportional hazard regression models adjusted for age, sex, ethnicity, alcohol consumption, smoking status, diet score, physical activity, annual household income, education, and Townsend Deprivation Index.

**Abbreviation:** MAFLD, metabolic-associated fatty liver disease; PM_2.5_, fine particulate matter with diameter <2.5 μm; PM_10_, particulate matter with diameter <10 μm; NO_2_, nitrogen dioxide; NO_x_, nitrogen oxides; CAD, coronary artery disease; IS, ischemic stroke; PAD, peripheral artery disease; CKD, chronic kidney disease.

**Supplemental Table 7. Hazard ratios (95% CIs) for the intrahepatic and extrahepatic morbidity and all-cause mortality associated with the four air pollutants among participants with different subtypes of MAFLD** **after further adjusting for biomarkers**

| **Air pollution** | **MAFLD subtype** | | | |
| --- | --- | --- | --- | --- |
|  | **Non-MAFLD** | **MAFLD-diabetes** | **MAFLD-lean** | **MAFLD-overweight/obesity** |
| **Cirrhosis** |  |  |  |  |
| PM_2.5_ |  |  |  |  |
| Low | Ref | 3.42 (3.06, 3.81) | 2.10 (1.61, 2.74) | 2.04 (1.91, 2.17) |
| Intermediate | 1.00 (0.93, 1.07) | 4.12 (3.67, 4.63) | 2.69 (2.03, 3.58) | 2.34 (2.18, 2.51) |
| High | 1.03 (0.96, 1.12) | 4.86 (4.32, 5.46) | 4.25 (3.31, 5.47) | 2.48 (2.30, 2.68) |
| PM_10_ |  |  |  |  |
| Low | Ref | 3.69 (3.31, 4.12) | 2.43 (1.87, 3.15) | 2.16 (2.02, 2.30) |
| Intermediate | 1.07 (0.99, 1.14) | 4.18 (3.73, 4.69) | 2.39 (1.79, 3.21) | 2.36 (2.20, 2.53) |
| High | 1.13 (1.05, 1.22) | 4.99 (4.44, 5.62) | 4.63 (3.60, 5.94) | 2.64 (2.44, 2.85) |
| NO_2_ |  |  |  |  |
| Low | Ref | 3.55 (3.17, 3.97) | 2.29 (1.75, 2.99) | 2.04 (1.91, 2.18) |
| Intermediate | 0.96 (0.90, 1.03) | 3.93 (3.49, 4.41) | 2.29 (1.70, 3.09) | 2.30 (2.14, 2.46) |
| High | 1.11 (1.02, 1.19) | 4.85 (4.33, 5.44) | 4.35 (3.42, 5.54) | 2.55 (2.36, 2.75) |
| NO_x_ |  |  |  |  |
| Low | Ref | 3.57 (3.19, 4.00) | 2.27 (1.74, 2.97) | 2.04 (1.91, 2.18) |
| Intermediate | 0.96 (0.89, 1.03) | 3.83 (3.40, 4.31) | 2.25 (1.66, 3.04) | 2.28 (2.13, 2.45) |
| High | 1.10 (1.02, 1.19) | 4.89 (4.37, 5.48) | 4.40 (3.46, 5.59) | 2.54 (2.36, 2.75) |
| **Liver cancer** |  |  |  |  |
| PM_2.5_ |  |  |  |  |
| Low | Ref | 5.12 (3.69, 7.12) | 2.05 (0.82, 5.08) | 1.64 (1.28, 2.10) |
| Intermediate | 1.22 (0.95, 1.57) | 5.20 (3.61, 7.49) | 1.09 (0.26, 4.48) | 1.55 (1.17, 2.06) |
| High | 1.18 (0.88, 1.57) | 4.31 (2.81, 6.62) | 1.36 (0.33, 5.58) | 1.78 (1.33, 2.40) |
| PM_10_ |  |  |  |  |
| Low | Ref | 5.97 (4.31, 8.28) | 2.20 (0.89, 5.48) | 1.67 (1.29, 2.16) |
| Intermediate | 1.28 (1.00, 1.64) | 4.27 (2.91, 6.27) | 0.53 (0.07, 3.81) | 1.72 (1.31, 2.26) |
| High | 1.30 (0.98, 1.73) | 5.05 (3.32, 7.67) | 2.27 (0.71, 7.28) | 1.83 (1.36, 2.46) |
| NO_2_ |  |  |  |  |
| Low | Ref | 4.18 (2.91, 6.02) | 2.10 (0.85, 5.19) | 1.54 (1.20, 1.99) |
| Intermediate | 1.07 (0.83, 1.38) | 5.61 (3.99, 7.87) | 0.49 (0.07, 3.57) | 1.59 (1.22, 2.08) |
| High | 1.15 (0.86, 1.53) | 4.37 (2.92, 6.53) | 1.86 (0.58, 6.01) | 1.60 (1.19, 2.15) |
| NO_x_ |  |  |  |  |
| Low | Ref | 4.21 (2.93, 6.06) | 2.11 (0.85, 5.24) | 1.55 (1.20, 2.00) |
| Intermediate | 1.06 (0.83, 1.37) | 5.78 (4.13, 8.10) | 0.50 (0.07, 3.61) | 1.62 (1.24, 2.12) |
| High | 1.19 (0.90, 1.59) | 4.28 (2.85, 6.43) | 1.88 (0.58, 6.05) | 1.60 (1.19, 2.14) |
| **CAD** |  |  |  |  |
| PM_2.5_ |  |  |  |  |
| Low | Ref | 1.65 (1.53, 1.78) | 0.98 (0.81, 1.19) | 1.20 (1.16, 1.25) |
| Intermediate | 0.97 (0.93, 1.01) | 2.03 (1.88, 2.20) | 1.29 (1.07, 1.55) | 1.24 (1.18, 1.29) |
| High | 0.92 (0.88, 0.96) | 1.90 (1.75, 2.06) | 1.18 (0.96, 1.46) | 1.23 (1.18, 1.30) |
| PM_10_ |  |  |  |  |
| Low | Ref | 1.71 (1.58, 1.84) | 0.93 (0.76, 1.14) | 1.20 (1.15, 1.24) |
| Intermediate | 0.99 (0.95, 1.03) | 1.92 (1.78, 2.07) | 1.35 (1.13, 1.60) | 1.26 (1.21, 1.32) |
| High | 0.95 (0.90, 0.99) | 2.01 (1.86, 2.18) | 1.21 (0.98, 1.50) | 1.27 (1.21, 1.33) |
| NO_2_ |  |  |  |  |
| Low | Ref | 1.69 (1.57, 1.83) | 1.07 (0.89, 1.29) | 1.23 (1.18, 1.28) |
| Intermediate | 0.97 (0.93, 1.01) | 1.89 (1.75, 2.05) | 1.15 (0.95, 1.39) | 1.20 (1.15, 1.25) |
| High | 0.96 (0.92, 1.01) | 2.01 (1.86, 2.19) | 1.23 (1.00, 1.51) | 1.27 (1.21, 1.34) |
| NO_x_ |  |  |  |  |
| Low | Ref | 1.70 (1.58, 1.84) | 1.08 (0.89, 1.30) | 1.23 (1.18, 1.28) |
| Intermediate | 0.97 (0.94, 1.01) | 1.90 (1.76, 2.05) | 1.16 (0.96, 1.41) | 1.21 (1.16, 1.26) |
| High | 0.97 (0.93, 1.01) | 2.02 (1.86, 2.19) | 1.22 (0.99, 1.50) | 1.28 (1.22, 1.34) |
| **IS** |  |  |  |  |
| PM_2.5_ |  |  |  |  |
| Low | Ref | 1.98 (1.70, 2.30) | 1.16 (0.77, 1.74) | 1.16 (1.06, 1.27) |
| Intermediate | 0.98 (0.90, 1.07) | 1.94 (1.65, 2.29) | 0.95 (0.59, 1.54) | 1.19 (1.08, 1.32) |
| High | 0.92 (0.84, 1.02) | 1.80 (1.51, 2.16) | 1.34 (0.86, 2.09) | 1.09 (0.97, 1.21) |
| PM_10_ |  |  |  |  |
| Low | Ref | 1.90 (1.63, 2.22) | 1.00 (0.65, 1.55) | 1.19 (1.09, 1.30) |
| Intermediate | 0.97 (0.89, 1.05) | 1.98 (1.69, 2.33) | 1.13 (0.74, 1.74) | 1.14 (1.03, 1.25) |
| High | 0.92 (0.83, 1.01) | 1.83 (1.53, 2.19) | 1.33 (0.84, 2.11) | 1.10 (0.99, 1.22) |
| NO_2_ |  |  |  |  |
| Low | Ref | 1.90 (1.61, 2.23) | 1.33 (0.90, 1.96) | 1.21 (1.10, 1.32) |
| Intermediate | 0.98 (0.90, 1.07) | 2.10 (1.79, 2.46) | 1.07 (0.68, 1.69) | 1.19 (1.08, 1.31) |
| High | 1.01 (0.92, 1.11) | 1.89 (1.60, 2.25) | 1.07 (0.65, 1.76) | 1.13 (1.01, 1.26) |
| NO_x_ |  |  |  |  |
| Low | Ref | 1.87 (1.58, 2.20) | 1.34 (0.90, 1.97) | 1.21 (1.11, 1.33) |
| Intermediate | 0.99 (0.91, 1.08) | 2.12 (1.81, 2.49) | 1.02 (0.64, 1.63) | 1.20 (1.09, 1.32) |
| High | 1.01 (0.91, 1.11) | 1.92 (1.62, 2.27) | 1.14 (0.70, 1.85) | 1.12 (1.01, 1.25) |
| **PAD** |  |  |  |  |
| PM_2.5_ |  |  |  |  |
| Low | Ref | 2.15 (1.85, 2.48) | 1.37 (0.98, 1.92) | 0.98 (0.90, 1.08) |
| Intermediate | 1.04 (0.94, 1.15) | 2.79 (2.39, 3.26) | 1.85 (1.29, 2.64) | 0.99 (0.88, 1.10) |
| High | 1.03 (0.92, 1.15) | 2.48 (2.07, 2.97) | 0.61 (0.30, 1.23) | 0.96 (0.84, 1.10) |
| PM_10_ |  |  |  |  |
| Low | Ref | 2.39 (2.07, 2.76) | 1.37 (0.97, 1.94) | 0.99 (0.90, 1.09) |
| Intermediate | 1.04 (0.95, 1.14) | 2.44 (2.09, 2.85) | 1.58 (1.09, 2.27) | 0.98 (0.88, 1.09) |
| High | 1.04 (0.93, 1.16) | 2.47 (2.06, 2.95) | 0.94 (0.53, 1.68) | 0.97 (0.85, 1.10) |
| NO_2_ |  |  |  |  |
| Low | Ref | 2.23 (1.91, 2.60) | 1.13 (0.76, 1.68) | 0.98 (0.89, 1.08) |
| Intermediate | 0.96 (0.87, 1.06) | 2.55 (2.19, 2.97) | 1.86 (1.32, 2.62) | 0.98 (0.88, 1.09) |
| High | 1.08 (0.97, 1.20) | 2.44 (2.05, 2.90) | 0.97 (0.58, 1.62) | 0.94 (0.83, 1.07) |
| NO_x_ |  |  |  |  |
| Low | Ref | 2.27 (1.95, 2.65) | 1.18 (0.80, 1.73) | 0.98 (0.89, 1.08) |
| Intermediate | 0.97 (0.88, 1.06) | 2.51 (2.15, 2.94) | 1.89 (1.34, 2.66) | 1.00 (0.89, 1.11) |
| High | 1.10 (0.99, 1.23) | 2.49 (2.10, 2.95) | 0.91 (0.53, 1.55) | 0.95 (0.84, 1.08) |
| **CKD** |  |  |  |  |
| PM_2.5_ |  |  |  |  |
| Low | Ref | 3.47 (3.18, 3.79) | 1.16 (0.89, 1.53) | 1.25 (1.19, 1.33) |
| Intermediate | 1.17 (1.10, 1.23) | 3.98 (3.62, 4.37) | 1.62 (1.21, 2.17) | 1.39 (1.31, 1.48) |
| High | 1.20 (1.12, 1.27) | 3.90 (3.52, 4.31) | 1.02 (0.67, 1.54) | 1.51 (1.41, 1.63) |
| PM_10_ |  |  |  |  |
| Low | Ref | 3.48 (3.19, 3.79) | 1.07 (0.79, 1.43) | 1.22 (1.15, 1.29) |
| Intermediate | 1.10 (1.04, 1.16) | 3.76 (3.41, 4.14) | 1.53 (1.16, 2.02) | 1.39 (1.31, 1.48) |
| High | 1.20 (1.13, 1.28) | 3.90 (3.52, 4.31) | 1.16 (0.79, 1.72) | 1.48 (1.38, 1.59) |
| NO_2_ |  |  |  |  |
| Low | Ref | 3.38 (3.07, 3.71) | 1.17 (0.90, 1.52) | 1.25 (1.18, 1.33) |
| Intermediate | 1.11 (1.05, 1.18) | 3.81 (3.48, 4.18) | 1.27 (0.91, 1.76) | 1.36 (1.28, 1.45) |
| High | 1.11 (1.04, 1.19) | 3.79 (3.43, 4.18) | 1.34 (0.94, 1.91) | 1.38 (1.29, 1.48) |
| NO_x_ |  |  |  |  |
| Low | Ref | 3.40 (3.09, 3.73) | 1.16 (0.89, 1.51) | 1.25 (1.18, 1.32) |
| Intermediate | 1.10 (1.04, 1.16) | 3.74 (3.41, 4.10) | 1.21 (0.86, 1.69) | 1.35 (1.27, 1.44) |
| High | 1.12 (1.05, 1.20) | 3.82 (3.46, 4.21) | 1.42 (1.01, 2.01) | 1.39 (1.30, 1.49) |
| **All-cause mortality** |  |  |  |  |
| PM_2.5_ |  |  |  |  |
| Low | Ref | 1.93 (1.80, 2.06) | 1.43 (1.22, 1.67) | 1.16 (1.12, 1.21) |
| Intermediate | 1.00 (0.96, 1.04) | 1.77 (1.63, 1.92) | 1.26 (1.03, 1.53) | 1.15 (1.09, 1.20) |
| High | 1.02 (0.97, 1.06) | 1.80 (1.65, 1.97) | 1.81 (1.49, 2.20) | 1.12 (1.06, 1.18) |
| PM_10_ |  |  |  |  |
| Low | Ref | 1.97 (1.84, 2.10) | 1.42 (1.20, 1.66) | 1.15 (1.11, 1.20) |
| Intermediate | 0.97 (0.94, 1.01) | 1.65 (1.51, 1.79) | 1.30 (1.08, 1.57) | 1.11 (1.06, 1.16) |
| High | 1.02 (0.98, 1.07) | 1.85 (1.70, 2.03) | 1.75 (1.44, 2.14) | 1.14 (1.09, 1.21) |
| NO_2_ |  |  |  |  |
| Low | Ref | 1.91 (1.78, 2.05) | 1.34 (1.13, 1.59) | 1.17 (1.13, 1.22) |
| Intermediate | 0.99 (0.95, 1.02) | 1.84 (1.70, 2.00) | 1.60 (1.35, 1.90) | 1.11 (1.06, 1.16) |
| High | 1.05 (1.00, 1.09) | 1.79 (1.64, 1.95) | 1.52 (1.24, 1.86) | 1.17 (1.11, 1.23) |
| NO_x_ |  |  |  |  |
| Low | Ref | 1.93 (1.80, 2.06) | 1.35 (1.14, 1.59) | 1.17 (1.13, 1.22) |
| Intermediate | 0.98 (0.94, 1.02) | 1.81 (1.67, 1.97) | 1.57 (1.32, 1.87) | 1.11 (1.06, 1.16) |
| High | 1.05 (1.01, 1.10) | 1.80 (1.65, 1.96) | 1.54 (1.26, 1.89) | 1.16 (1.11, 1.23) |

The hazard ratios and 95% confidence intervals were calculated using the time-varying Cox proportional hazard regression models adjusted for age, sex, ethnicity, alcohol consumption, smoking status, diet score, physical activity, annual household income, education, Townsend Deprivation Index, triglycerides, high-density lipoprotein cholesterol, C-reactive protein, and estimated Glomerular Filtration Rate.

**Abbreviation:** MAFLD, metabolic-associated fatty liver disease; PM_2.5_, fine particulate matter with diameter <2.5 μm; PM_10_, particulate matter with diameter <10 μm; NO_2_, nitrogen dioxide; NO_x_, nitrogen oxides; CAD, coronary artery disease; IS, ischemic stroke; PAD, peripheral artery disease; CKD, chronic kidney disease.

**Supplemental Table 8. Hazard ratios (95% CIs) for the intrahepatic and extrahepatic morbidity and all-cause mortality associated with the four air pollutants among participants with different subtypes of MAFLD** **after varying the exposure time window to 1-year averages**

| **Air pollution** | **MAFLD subtype** | | | |
| --- | --- | --- | --- | --- |
|  | **Non-MAFLD** | **MAFLD-diabetes** | **MAFLD-lean** | **MAFLD-overweight/obesity** |
| **Cirrhosis** |  |  |  |  |
| PM_2.5_ |  |  |  |  |
| Low | Ref | 3.74 (3.39, 4.14) | 2.20 (1.72, 2.82) | 2.19 (2.07, 2.32) |
| Intermediate | 0.95 (0.89, 1.02) | 4.46 (4.00, 4.98) | 3.07 (2.35, 4.00) | 2.33 (2.19, 2.49) |
| High | 0.91 (0.84, 0.99) | 5.06 (4.52, 5.67) | 3.89 (3.01, 5.03) | 2.54 (2.36, 2.74) |
| PM_10_ |  |  |  |  |
| Low | Ref | 4.22 (3.82, 4.66) | 2.47 (1.93, 3.15) | 2.29 (2.16, 2.43) |
| Intermediate | 0.97 (0.91, 1.04) | 4.24 (3.79, 4.73) | 2.55 (1.92, 3.38) | 2.36 (2.21, 2.52) |
| High | 1.04 (0.96, 1.12) | 5.14 (4.59, 5.75) | 4.45 (3.49, 5.68) | 2.67 (2.49, 2.87) |
| NO_2_ |  |  |  |  |
| Low | Ref | 3.84 (3.46, 4.27) | 2.47 (1.92, 3.17) | 2.19 (2.06, 2.32) |
| Intermediate | 0.94 (0.88, 1.01) | 4.41 (3.95, 4.92) | 2.35 (1.76, 3.16) | 2.43 (2.27, 2.59) |
| High | 1.05 (0.98, 1.14) | 5.33 (4.78, 5.95) | 4.51 (3.56, 5.71) | 2.70 (2.52, 2.90) |
| NO_x_ |  |  |  |  |
| Low | Ref | 3.87 (3.49, 4.30) | 2.45 (1.91, 3.14) | 2.19 (2.07, 2.33) |
| Intermediate | 0.94 (0.88, 1.01) | 4.34 (3.88, 4.85) | 2.37 (1.77, 3.18) | 2.42 (2.27, 2.59) |
| High | 1.06 (0.98, 1.14) | 5.40 (4.84, 6.02) | 4.54 (3.58, 5.75) | 2.70 (2.51, 2.90) |
| **Liver cancer** |  |  |  |  |
| PM_2.5_ |  |  |  |  |
| Low | Ref | 5.45 (4.04, 7.34) | 1.90 (0.78, 4.66) | 1.63 (1.31, 2.03) |
| Intermediate | 1.17 (0.92, 1.49) | 5.10 (3.56, 7.29) | 1.66 (0.52, 5.28) | 1.62 (1.25, 2.10) |
| High | 1.29 (0.98, 1.69) | 5.15 (3.45, 7.69) | 2.77 (1.02, 7.56) | 1.90 (1.43, 2.52) |
| PM_10_ |  |  |  |  |
| Low | Ref | 6.66 (4.96, 8.95) | 2.15 (0.87, 5.27) | 1.66 (1.32, 2.09) |
| Intermediate | 1.34 (1.05, 1.70) | 4.62 (3.19, 6.70) | 2.20 (0.80, 6.04) | 1.94 (1.51, 2.49) |
| High | 1.36 (1.04, 1.78) | 5.23 (3.51, 7.80) | 2.20 (0.70, 6.92) | 1.87 (1.41, 2.47) |
| NO_2_ |  |  |  |  |
| Low | Ref | 4.58 (3.31, 6.35) | 2.76 (1.28, 5.95) | 1.60 (1.28, 2.00) |
| Intermediate | 1.08 (0.85, 1.37) | 5.60 (4.04, 7.75) | 0.47 (0.07, 3.40) | 1.69 (1.33, 2.16) |
| High | 1.06 (0.80, 1.40) | 4.42 (2.99, 6.52) | 2.37 (0.87, 6.47) | 1.37 (1.02, 1.83) |
| NO_x_ |  |  |  |  |
| Low | Ref | 4.60 (3.33, 6.36) | 2.73 (1.27, 5.87) | 1.60 (1.28, 2.00) |
| Intermediate | 1.05 (0.83, 1.33) | 5.39 (3.87, 7.50) | 0.47 (0.07, 3.40) | 1.64 (1.29, 2.10) |
| High | 1.08 (0.82, 1.43) | 4.54 (3.09, 6.68) | 2.37 (0.87, 6.46) | 1.38 (1.04, 1.85) |
| **CAD** |  |  |  |  |
| PM_2.5_ |  |  |  |  |
| Low | Ref | 1.99 (1.86, 2.13) | 1.13 (0.95, 1.35) | 1.37 (1.33, 1.42) |
| Intermediate | 0.89 (0.85, 0.92) | 2.14 (1.98, 2.31) | 1.29 (1.07, 1.57) | 1.33 (1.27, 1.38) |
| High | 0.86 (0.82, 0.90) | 2.13 (1.97, 2.31) | 1.32 (1.08, 1.61) | 1.34 (1.28, 1.41) |
| PM_10_ |  |  |  |  |
| Low | Ref | 2.01 (1.87, 2.15) | 1.14 (0.95, 1.37) | 1.38 (1.33, 1.42) |
| Intermediate | 0.93 (0.90, 0.97) | 2.21 (2.05, 2.39) | 1.37 (1.15, 1.64) | 1.37 (1.32, 1.42) |
| High | 0.88 (0.84, 0.92) | 2.16 (1.99, 2.33) | 1.28 (1.04, 1.58) | 1.39 (1.33, 1.45) |
| NO_2_ |  |  |  |  |
| Low | Ref | 2.02 (1.88, 2.16) | 1.24 (1.04, 1.48) | 1.40 (1.35, 1.45) |
| Intermediate | 0.96 (0.93, 1.00) | 2.26 (2.09, 2.43) | 1.25 (1.03, 1.52) | 1.40 (1.34, 1.45) |
| High | 0.95 (0.91, 0.99) | 2.30 (2.12, 2.49) | 1.40 (1.15, 1.71) | 1.46 (1.40, 1.53) |
| NO_x_ |  |  |  |  |
| Low | Ref | 2.03 (1.89, 2.17) | 1.22 (1.02, 1.45) | 1.41 (1.36, 1.46) |
| Intermediate | 0.96 (0.92, 0.99) | 2.24 (2.08, 2.41) | 1.29 (1.07, 1.56) | 1.38 (1.33, 1.44) |
| High | 0.95 (0.91, 0.99) | 2.30 (2.12, 2.49) | 1.39 (1.14, 1.70) | 1.46 (1.40, 1.53) |
| **IS** |  |  |  |  |
| PM_2.5_ |  |  |  |  |
| Low | Ref | 1.97 (1.70, 2.28) | 1.06 (0.72, 1.57) | 1.20 (1.10, 1.30) |
| Intermediate | 1.00 (0.92, 1.09) | 2.07 (1.76, 2.42) | 0.96 (0.60, 1.53) | 1.25 (1.14, 1.37) |
| High | 0.94 (0.85, 1.03) | 1.98 (1.66, 2.36) | 1.47 (0.97, 2.24) | 1.12 (1.01, 1.25) |
| PM_10_ |  |  |  |  |
| Low | Ref | 1.94 (1.67, 2.26) | 0.90 (0.59, 1.38) | 1.19 (1.10, 1.30) |
| Intermediate | 0.93 (0.86, 1.01) | 2.00 (1.71, 2.34) | 1.19 (0.80, 1.79) | 1.18 (1.08, 1.29) |
| High | 0.92 (0.84, 1.01) | 1.90 (1.60, 2.26) | 1.31 (0.85, 2.03) | 1.10 (0.99, 1.22) |
| NO_2_ |  |  |  |  |
| Low | Ref | 1.99 (1.70, 2.32) | 1.13 (0.76, 1.68) | 1.24 (1.14, 1.35) |
| Intermediate | 0.97 (0.90, 1.06) | 1.94 (1.65, 2.28) | 1.17 (0.77, 1.77) | 1.20 (1.09, 1.31) |
| High | 0.97 (0.88, 1.06) | 2.07 (1.76, 2.44) | 1.11 (0.69, 1.77) | 1.12 (1.01, 1.25) |
| NO_x_ |  |  |  |  |
| Low | Ref | 2.02 (1.73, 2.36) | 1.13 (0.76, 1.68) | 1.24 (1.14, 1.34) |
| Intermediate | 0.99 (0.91, 1.07) | 1.90 (1.61, 2.24) | 1.18 (0.78, 1.78) | 1.20 (1.10, 1.31) |
| High | 0.98 (0.89, 1.07) | 2.12 (1.80, 2.50) | 1.12 (0.70, 1.80) | 1.15 (1.04, 1.28) |
| **PAD** |  |  |  |  |
| PM_2.5_ |  |  |  |  |
| Low | Ref | 2.50 (2.18, 2.87) | 1.60 (1.18, 2.18) | 1.13 (1.04, 1.23) |
| Intermediate | 1.04 (0.94, 1.14) | 3.21 (2.76, 3.73) | 1.68 (1.13, 2.48) | 1.15 (1.03, 1.27) |
| High | 0.99 (0.88, 1.11) | 2.68 (2.23, 3.22) | 1.00 (0.56, 1.78) | 1.07 (0.94, 1.21) |
| PM_10_ |  |  |  |  |
| Low | Ref | 2.61 (2.26, 3.01) | 1.71 (1.25, 2.33) | 1.17 (1.07, 1.28) |
| Intermediate | 1.04 (0.95, 1.14) | 3.04 (2.62, 3.52) | 1.59 (1.08, 2.33) | 1.11 (1.00, 1.23) |
| High | 1.03 (0.93, 1.15) | 2.75 (2.31, 3.29) | 1.00 (0.56, 1.77) | 1.09 (0.96, 1.23) |
| NO_2_ |  |  |  |  |
| Low | Ref | 2.53 (2.18, 2.94) | 1.39 (0.97, 1.99) | 1.13 (1.03, 1.24) |
| Intermediate | 0.99 (0.90, 1.09) | 2.97 (2.56, 3.45) | 1.91 (1.35, 2.70) | 1.15 (1.04, 1.28) |
| High | 1.09 (0.98, 1.21) | 2.95 (2.49, 3.49) | 1.19 (0.73, 1.97) | 1.11 (0.99, 1.26) |
| NO_x_ |  |  |  |  |
| Low | Ref | 2.45 (2.11, 2.85) | 1.37 (0.96, 1.97) | 1.14 (1.04, 1.24) |
| Intermediate | 0.99 (0.90, 1.09) | 3.00 (2.58, 3.48) | 1.88 (1.32, 2.67) | 1.15 (1.04, 1.27) |
| High | 1.11 (1.00, 1.24) | 3.11 (2.63, 3.68) | 1.29 (0.80, 2.09) | 1.13 (1.00, 1.27) |
| **CKD** |  |  |  |  |
| PM_2.5_ |  |  |  |  |
| Low | Ref | 3.52 (3.25, 3.81) | 1.23 (0.95, 1.58) | 1.58 (1.50, 1.65) |
| Intermediate | 1.10 (1.04, 1.16) | 4.04 (3.70, 4.41) | 1.49 (1.11, 2.01) | 1.62 (1.53, 1.72) |
| High | 1.13 (1.06, 1.20) | 3.91 (3.55, 4.30) | 0.98 (0.66, 1.47) | 1.81 (1.69, 1.92) |
| PM_10_ |  |  |  |  |
| Low | Ref | 3.57 (3.30, 3.88) | 1.14 (0.87, 1.49) | 1.53 (1.45, 1.60) |
| Intermediate | 1.06 (1.01, 1.12) | 3.87 (3.55, 4.21) | 1.47 (1.11, 1.95) | 1.66 (1.57, 1.75) |
| High | 1.09 (1.03, 1.16) | 3.72 (3.39, 4.09) | 1.08 (0.74, 1.57) | 1.73 (1.63, 1.84) |
| NO_2_ |  |  |  |  |
| Low | Ref | 3.59 (3.31, 3.88) | 1.16 (0.89, 1.52) | 1.56 (1.49, 1.64) |
| Intermediate | 1.06 (1.01, 1.12) | 3.58 (3.26, 3.94) | 1.19 (0.87, 1.63) | 1.62 (1.54, 1.72) |
| High | 1.05 (0.99, 1.12) | 3.93 (3.59, 4.30) | 1.37 (0.98, 1.91) | 1.66 (1.55, 1.76) |
| NO_x_ |  |  |  |  |
| Low | Ref | 3.56 (3.29, 3.86) | 1.16 (0.89, 1.51) | 1.57 (1.50, 1.65) |
| Intermediate | 1.06 (1.01, 1.12) | 3.58 (3.26, 3.94) | 1.26 (0.93, 1.71) | 1.61 (1.52, 1.70) |
| High | 1.06 (0.99, 1.12) | 3.96 (3.62, 4.33) | 1.30 (0.92, 1.83) | 1.66 (1.56, 1.77) |
| **All-cause mortality** |  |  |  |  |
| PM_2.5_ |  |  |  |  |
| Low | Ref | 1.90 (1.78, 2.02) | 1.41 (1.23, 1.62) | 1.15 (1.11, 1.19) |
| Intermediate | 0.99 (0.96, 1.03) | 1.77 (1.64, 1.92) | 1.25 (1.03, 1.51) | 1.11 (1.06, 1.16) |
| High | 1.01 (0.96, 1.05) | 1.71 (1.56, 1.87) | 1.77 (1.47, 2.14) | 1.10 (1.05, 1.16) |
| PM_10_ |  |  |  |  |
| Low | Ref | 1.95 (1.83, 2.08) | 1.45 (1.25, 1.67) | 1.15 (1.11, 1.19) |
| Intermediate | 1.01 (0.98, 1.05) | 1.73 (1.59, 1.87) | 1.20 (1.00, 1.45) | 1.14 (1.09, 1.19) |
| High | 1.03 (0.98, 1.07) | 1.78 (1.63, 1.94) | 1.86 (1.55, 2.23) | 1.13 (1.07, 1.19) |
| NO_2_ |  |  |  |  |
| Low | Ref | 1.90 (1.78, 2.03) | 1.38 (1.19, 1.60) | 1.16 (1.12, 1.21) |
| Intermediate | 1.00 (0.97, 1.04) | 1.81 (1.67, 1.96) | 1.43 (1.20, 1.70) | 1.11 (1.07, 1.16) |
| High | 1.06 (1.02, 1.11) | 1.82 (1.67, 1.98) | 1.69 (1.40, 2.03) | 1.16 (1.11, 1.22) |
| NO_x_ |  |  |  |  |
| Low | Ref | 1.92 (1.80, 2.05) | 1.34 (1.15, 1.55) | 1.16 (1.12, 1.21) |
| Intermediate | 1.00 (0.97, 1.04) | 1.78 (1.64, 1.93) | 1.51 (1.27, 1.79) | 1.11 (1.07, 1.16) |
| High | 1.07 (1.02, 1.12) | 1.83 (1.68, 1.99) | 1.69 (1.40, 2.04) | 1.17 (1.11, 1.23) |

The hazard ratios and 95% confidence intervals were calculated using the time-varying Cox proportional hazard regression models adjusted for age, sex, ethnicity, alcohol consumption, smoking status, diet score, physical activity, annual household income, education, and Townsend Deprivation Index.

**Abbreviation:** MAFLD, metabolic-associated fatty liver disease; PM_2.5_, fine particulate matter with diameter <2.5 μm; PM_10_, particulate matter with diameter <10 μm; NO_2_, nitrogen dioxide; NO_x_, nitrogen oxides; CAD, coronary artery disease; IS, ischemic stroke; PAD, peripheral artery disease; CKD, chronic kidney disease.

**Supplemental Table 9. Hazard ratios (95% CIs) for the intrahepatic and extrahepatic morbidity and all-cause mortality associated with the four air pollutants among participants with different subtypes of MAFLD** **after varying the exposure time window to 5-year averages**

| **Air pollution** | **MAFLD subtype** | | | |
| --- | --- | --- | --- | --- |
|  | **Non-MAFLD** | **MAFLD-diabetes** | **MAFLD-lean** | **MAFLD-overweight/obesity** |
| **Cirrhosis** |  |  |  |  |
| PM_2.5_ |  |  |  |  |
| Low | Ref | 3.91 (3.53, 4.34) | 2.25 (1.74, 2.92) | 2.23 (2.10, 2.37) |
| Intermediate | 1.01 (0.95, 1.09) | 4.71 (4.23, 5.25) | 2.94 (2.24, 3.85) | 2.59 (2.43, 2.76) |
| High | 1.09 (1.01, 1.17) | 5.49 (4.91, 6.13) | 4.54 (3.56, 5.80) | 2.75 (2.56, 2.96) |
| PM_10_ |  |  |  |  |
| Low | Ref | 4.11 (3.71, 4.56) | 2.51 (1.95, 3.23) | 2.33 (2.19, 2.48) |
| Intermediate | 1.08 (1.01, 1.15) | 4.84 (4.35, 5.40) | 2.57 (1.93, 3.41) | 2.58 (2.42, 2.75) |
| High | 1.15 (1.07, 1.24) | 5.63 (5.02, 6.30) | 5.13 (4.04, 6.52) | 2.94 (2.74, 3.16) |
| NO_2_ |  |  |  |  |
| Low | Ref | 4.05 (3.63, 4.51) | 2.53 (1.95, 3.27) | 2.23 (2.10, 2.37) |
| Intermediate | 0.98 (0.91, 1.04) | 4.40 (3.93, 4.91) | 2.44 (1.83, 3.25) | 2.50 (2.35, 2.67) |
| High | 1.09 (1.01, 1.17) | 5.30 (4.76, 5.90) | 4.39 (3.47, 5.55) | 2.70 (2.51, 2.89) |
| NO_x_ |  |  |  |  |
| Low | Ref | 4.04 (3.62, 4.50) | 2.42 (1.86, 3.15) | 2.23 (2.10, 2.37) |
| Intermediate | 0.97 (0.91, 1.04) | 4.38 (3.92, 4.89) | 2.63 (1.99, 3.46) | 2.49 (2.33, 2.65) |
| High | 1.08 (1.01, 1.17) | 5.30 (4.77, 5.90) | 4.30 (3.40, 5.45) | 2.70 (2.52, 2.90) |
| **Liver cancer** |  |  |  |  |
| PM_2.5_ |  |  |  |  |
| Low | Ref | 6.24 (4.65, 8.38) | 2.46 (1.08, 5.63) | 1.64 (1.31, 2.06) |
| Intermediate | 1.16 (0.91, 1.48) | 4.66 (3.25, 6.68) | 0.51 (0.07, 3.67) | 1.54 (1.19, 2.00) |
| High | 1.24 (0.95, 1.63) | 4.42 (2.94, 6.65) | 3.28 (1.33, 8.10) | 1.92 (1.46, 2.53) |
| PM_10_ |  |  |  |  |
| Low | Ref | 6.51 (4.86, 8.71) | 2.47 (1.08, 5.64) | 1.68 (1.34, 2.10) |
| Intermediate | 1.22 (0.96, 1.54) | 4.20 (2.90, 6.07) | 0.49 (0.07, 3.53) | 1.61 (1.25, 2.07) |
| High | 1.19 (0.90, 1.56) | 4.62 (3.07, 6.94) | 3.49 (1.42, 8.60) | 1.80 (1.36, 2.38) |
| NO_2_ |  |  |  |  |
| Low | Ref | 4.85 (3.49, 6.73) | 2.52 (1.10, 5.74) | 1.57 (1.25, 1.98) |
| Intermediate | 1.09 (0.85, 1.40) | 5.82 (4.18, 8.11) | 1.01 (0.25, 4.11) | 1.63 (1.27, 2.10) |
| High | 1.11 (0.83, 1.48) | 4.40 (2.93, 6.60) | 1.36 (0.34, 5.52) | 1.42 (1.05, 1.92) |
| NO_x_ |  |  |  |  |
| Low | Ref | 4.88 (3.51, 6.78) | 2.54 (1.11, 5.79) | 1.58 (1.25, 1.99) |
| Intermediate | 1.08 (0.84, 1.39) | 6.00 (4.31, 8.34) | 1.02 (0.25, 4.14) | 1.65 (1.28, 2.12) |
| High | 1.15 (0.86, 1.54) | 4.29 (2.84, 6.48) | 1.37 (0.34, 5.55) | 1.43 (1.06, 1.94) |
| **CAD** |  |  |  |  |
| PM_2.5_ |  |  |  |  |
| Low | Ref | 1.92 (1.78, 2.06) | 1.11 (0.92, 1.34) | 1.40 (1.35, 1.45) |
| Intermediate | 1.00 (0.96, 1.04) | 2.46 (2.29, 2.65) | 1.50 (1.26, 1.79) | 1.46 (1.40, 1.51) |
| High | 0.94 (0.90, 0.98) | 2.30 (2.13, 2.49) | 1.36 (1.11, 1.67) | 1.44 (1.38, 1.51) |
| PM_10_ |  |  |  |  |
| Low | Ref | 1.95 (1.82, 2.10) | 1.05 (0.87, 1.28) | 1.38 (1.34, 1.44) |
| Intermediate | 1.00 (0.96, 1.03) | 2.37 (2.20, 2.55) | 1.50 (1.27, 1.78) | 1.45 (1.40, 1.51) |
| High | 0.95 (0.91, 0.99) | 2.37 (2.19, 2.57) | 1.46 (1.20, 1.79) | 1.49 (1.42, 1.56) |
| NO_2_ |  |  |  |  |
| Low | Ref | 2.00 (1.86, 2.15) | 1.24 (1.03, 1.49) | 1.42 (1.37, 1.47) |
| Intermediate | 0.96 (0.93, 1.00) | 2.24 (2.08, 2.41) | 1.39 (1.17, 1.67) | 1.39 (1.33, 1.44) |
| High | 0.96 (0.92, 1.01) | 2.34 (2.17, 2.54) | 1.25 (1.01, 1.54) | 1.46 (1.40, 1.53) |
| NO_x_ |  |  |  |  |
| Low | Ref | 2.00 (1.86, 2.15) | 1.23 (1.02, 1.48) | 1.42 (1.37, 1.47) |
| Intermediate | 0.96 (0.93, 1.00) | 2.25 (2.08, 2.42) | 1.41 (1.18, 1.68) | 1.39 (1.33, 1.44) |
| High | 0.96 (0.92, 1.01) | 2.33 (2.15, 2.51) | 1.24 (1.01, 1.53) | 1.46 (1.40, 1.53) |
| **IS** |  |  |  |  |
| PM_2.5_ |  |  |  |  |
| Low | Ref | 2.04 (1.76, 2.37) | 0.99 (0.65, 1.50) | 1.21 (1.11, 1.32) |
| Intermediate | 0.98 (0.90, 1.07) | 1.94 (1.66, 2.28) | 1.25 (0.84, 1.85) | 1.20 (1.09, 1.31) |
| High | 0.90 (0.82, 0.99) | 1.91 (1.61, 2.27) | 1.16 (0.73, 1.83) | 1.10 (0.99, 1.22) |
| PM_10_ |  |  |  |  |
| Low | Ref | 1.97 (1.70, 2.29) | 0.91 (0.60, 1.40) | 1.21 (1.12, 1.32) |
| Intermediate | 0.96 (0.88, 1.04) | 1.96 (1.67, 2.30) | 1.27 (0.86, 1.87) | 1.20 (1.10, 1.31) |
| High | 0.93 (0.84, 1.02) | 1.97 (1.66, 2.34) | 1.24 (0.78, 1.95) | 1.08 (0.97, 1.20) |
| NO_2_ |  |  |  |  |
| Low | Ref | 1.86 (1.58, 2.19) | 1.33 (0.92, 1.94) | 1.25 (1.15, 1.36) |
| Intermediate | 0.99 (0.91, 1.07) | 2.12 (1.81, 2.48) | 0.97 (0.62, 1.52) | 1.20 (1.10, 1.32) |
| High | 0.98 (0.89, 1.07) | 2.07 (1.76, 2.43) | 1.13 (0.71, 1.78) | 1.14 (1.03, 1.27) |
| NO_x_ |  |  |  |  |
| Low | Ref | 1.87 (1.58, 2.20) | 1.37 (0.94, 1.99) | 1.25 (1.15, 1.36) |
| Intermediate | 1.02 (0.94, 1.11) | 2.16 (1.85, 2.53) | 0.92 (0.58, 1.47) | 1.24 (1.13, 1.35) |
| High | 0.99 (0.91, 1.09) | 2.12 (1.81, 2.49) | 1.20 (0.77, 1.87) | 1.17 (1.05, 1.29) |
| **PAD** |  |  |  |  |
| PM_2.5_ |  |  |  |  |
| Low | Ref | 2.42 (2.09, 2.80) | 1.42 (1.01, 2.00) | 1.18 (1.08, 1.29) |
| Intermediate | 1.06 (0.96, 1.16) | 3.28 (2.84, 3.80) | 2.11 (1.51, 2.95) | 1.11 (1.00, 1.24) |
| High | 1.04 (0.94, 1.16) | 2.89 (2.42, 3.44) | 0.91 (0.50, 1.65) | 1.10 (0.97, 1.24) |
| PM_10_ |  |  |  |  |
| Low | Ref | 2.61 (2.26, 3.01) | 1.59 (1.15, 2.20) | 1.16 (1.06, 1.27) |
| Intermediate | 1.09 (1.00, 1.20) | 2.95 (2.54, 3.43) | 1.68 (1.16, 2.43) | 1.19 (1.08, 1.32) |
| High | 1.05 (0.94, 1.17) | 3.12 (2.63, 3.71) | 1.24 (0.73, 2.10) | 1.09 (0.96, 1.24) |
| NO_2_ |  |  |  |  |
| Low | Ref | 2.45 (2.10, 2.86) | 1.25 (0.85, 1.85) | 1.16 (1.05, 1.27) |
| Intermediate | 0.97 (0.89, 1.07) | 3.02 (2.60, 3.50) | 2.04 (1.47, 2.85) | 1.14 (1.03, 1.26) |
| High | 1.10 (0.99, 1.22) | 2.94 (2.50, 3.46) | 1.23 (0.77, 1.96) | 1.07 (0.95, 1.20) |
| NO_x_ |  |  |  |  |
| Low | Ref | 2.48 (2.12, 2.90) | 1.27 (0.86, 1.87) | 1.15 (1.05, 1.27) |
| Intermediate | 0.99 (0.90, 1.08) | 3.05 (2.63, 3.53) | 2.12 (1.53, 2.94) | 1.15 (1.04, 1.27) |
| High | 1.11 (1.00, 1.23) | 2.93 (2.49, 3.46) | 1.16 (0.71, 1.87) | 1.09 (0.97, 1.23) |
| **CKD** |  |  |  |  |
| PM_2.5_ |  |  |  |  |
| Low | Ref | 3.54 (3.26, 3.85) | 1.09 (0.83, 1.44) | 1.57 (1.50, 1.65) |
| Intermediate | 1.18 (1.12, 1.24) | 4.20 (3.86, 4.58) | 1.86 (1.43, 2.40) | 1.74 (1.65, 1.84) |
| High | 1.12 (1.06, 1.19) | 3.98 (3.62, 4.37) | 0.87 (0.56, 1.33) | 1.81 (1.70, 1.93) |
| PM_10_ |  |  |  |  |
| Low | Ref | 3.49 (3.21, 3.79) | 1.04 (0.78, 1.38) | 1.54 (1.46, 1.62) |
| Intermediate | 1.08 (1.03, 1.14) | 4.03 (3.70, 4.38) | 1.55 (1.18, 2.04) | 1.70 (1.61, 1.80) |
| High | 1.17 (1.10, 1.24) | 4.03 (3.67, 4.42) | 1.22 (0.85, 1.76) | 1.81 (1.70, 1.93) |
| NO_2_ |  |  |  |  |
| Low | Ref | 3.46 (3.19, 3.75) | 1.13 (0.86, 1.49) | 1.56 (1.49, 1.65) |
| Intermediate | 1.05 (1.00, 1.11) | 3.76 (3.43, 4.12) | 1.29 (0.96, 1.74) | 1.61 (1.52, 1.70) |
| High | 1.03 (0.97, 1.10) | 3.77 (3.45, 4.12) | 1.25 (0.89, 1.76) | 1.61 (1.52, 1.72) |
| NO_x_ |  |  |  |  |
| Low | Ref | 3.46 (3.19, 3.76) | 1.12 (0.85, 1.48) | 1.56 (1.48, 1.64) |
| Intermediate | 1.05 (0.99, 1.10) | 3.76 (3.44, 4.12) | 1.32 (0.98, 1.76) | 1.62 (1.53, 1.71) |
| High | 1.06 (1.00, 1.12) | 3.82 (3.49, 4.17) | 1.26 (0.90, 1.77) | 1.64 (1.54, 1.75) |
| **All-cause mortality** |  |  |  |  |
| PM_2.5_ |  |  |  |  |
| Low | Ref | 1.89 (1.77, 2.02) | 1.43 (1.24, 1.65) | 1.15 (1.11, 1.19) |
| Intermediate | 1.01 (0.97, 1.05) | 1.75 (1.62, 1.89) | 1.30 (1.08, 1.56) | 1.13 (1.08, 1.18) |
| High | 1.04 (0.99, 1.08) | 1.86 (1.71, 2.03) | 1.77 (1.47, 2.13) | 1.16 (1.10, 1.21) |
| PM_10_ |  |  |  |  |
| Low | Ref | 1.93 (1.80, 2.06) | 1.36 (1.18, 1.58) | 1.14 (1.10, 1.18) |
| Intermediate | 1.00 (0.96, 1.04) | 1.69 (1.57, 1.83) | 1.39 (1.17, 1.64) | 1.14 (1.10, 1.19) |
| High | 1.06 (1.02, 1.11) | 1.91 (1.75, 2.09) | 1.83 (1.51, 2.21) | 1.16 (1.10, 1.22) |
| NO_2_ |  |  |  |  |
| Low | Ref | 1.87 (1.75, 2.00) | 1.38 (1.19, 1.61) | 1.16 (1.11, 1.20) |
| Intermediate | 0.97 (0.94, 1.01) | 1.82 (1.68, 1.97) | 1.52 (1.29, 1.80) | 1.10 (1.06, 1.14) |
| High | 1.06 (1.02, 1.11) | 1.80 (1.66, 1.96) | 1.50 (1.24, 1.82) | 1.16 (1.10, 1.21) |
| NO_x_ |  |  |  |  |
| Low | Ref | 1.86 (1.74, 1.99) | 1.37 (1.18, 1.60) | 1.16 (1.12, 1.20) |
| Intermediate | 0.98 (0.95, 1.02) | 1.85 (1.71, 2.00) | 1.54 (1.31, 1.82) | 1.10 (1.06, 1.15) |
| High | 1.06 (1.02, 1.11) | 1.80 (1.66, 1.96) | 1.51 (1.24, 1.83) | 1.16 (1.11, 1.22) |

The hazard ratios and 95% confidence intervals were calculated using the time-varying Cox proportional hazard regression models adjusted for age, sex, ethnicity, alcohol consumption, smoking status, diet score, physical activity, annual household income, education, and Townsend Deprivation Index.

**Abbreviation:** MAFLD, metabolic-associated fatty liver disease; PM_2.5_, fine particulate matter with diameter <2.5 μm; PM_10_, particulate matter with diameter <10 μm; NO_2_, nitrogen dioxide; NO_x_, nitrogen oxides; CAD, coronary artery disease; IS, ischemic stroke; PAD, peripheral artery disease; CKD, chronic kidney disease.

**Supplemental Table 10. Hazard ratios (95% CIs) for the intrahepatic and extrahepatic morbidity and all-cause mortality associated with the four air pollutants among participants with different subtypes of MAFLD** **in two-pollutant model**

| **Air pollution** | **MAFLD subtype** | | | |
| --- | --- | --- | --- | --- |
|  | **Non-MAFLD** | **MAFLD-diabetes** | **MAFLD-lean** | **MAFLD-overweight/obesity** |
| **Cirrhosis** |  |  |  |  |
| PM_2.5_+NO_2_ |  |  |  |  |
| Low | Ref | 3.85 (3.49, 4.26) | 2.18 (1.67, 2.84) | 2.20 (2.08, 2.34) |
| Intermediate | 0.91 (0.84, 0.97) | 4.16 (3.73, 4.65) | 2.55 (1.94, 3.37) | 2.35 (2.20, 2.51) |
| High | 0.92 (0.84, 0.99) | 4.77 (4.26, 5.34) | 3.83 (3.01, 4.88) | 2.33 (2.15, 2.52) |
| PM_2.5_+NO_x_ |  |  |  |  |
| Low | Ref | 3.86 (3.49, 4.26) | 2.18 (1.67, 2.84) | 2.21 (2.08, 2.34) |
| Intermediate | 0.90 (0.84, 0.97) | 4.15 (3.72, 4.64) | 2.55 (1.93, 3.36) | 2.34 (2.19, 2.50) |
| High | 0.91 (0.84, 0.99) | 4.73 (4.23, 5.30) | 3.80 (2.99, 4.84) | 2.31 (2.13, 2.50) |
| PM_10_+NO_2_ |  |  |  |  |
| Low | Ref | 4.15 (3.74, 4.59) | 2.61 (2.03, 3.35) | 2.33 (2.20, 2.48) |
| Intermediate | 1.04 (0.97, 1.11) | 4.57 (4.11, 5.09) | 2.35 (1.76, 3.14) | 2.50 (2.34, 2.67) |
| High | 1.04 (0.96, 1.13) | 5.12 (4.57, 5.73) | 4.33 (3.40, 5.52) | 2.62 (2.42, 2.83) |
| PM_10_+NO_x_ |  |  |  |  |
| Low | Ref | 4.15 (3.74, 4.59) | 2.61 (2.03, 3.35) | 2.33 (2.20, 2.48) |
| Intermediate | 1.03 (0.96, 1.11) | 4.56 (4.10, 5.07) | 2.34 (1.75, 3.14) | 2.50 (2.34, 2.66) |
| High | 1.03 (0.95, 1.12) | 5.08 (4.54, 5.69) | 4.30 (3.38, 5.48) | 2.60 (2.40, 2.81) |
| NO_2_+PM_2.5_ |  |  |  |  |
| Low | Ref | 3.96 (3.56, 4.41) | 2.45 (1.88, 3.18) | 2.24 (2.11, 2.38) |
| Intermediate | 0.89 (0.83, 0.96) | 4.09 (3.67, 4.55) | 2.29 (1.72, 3.05) | 2.27 (2.13, 2.42) |
| High | 1.00 (0.92, 1.08) | 4.93 (4.41, 5.50) | 3.89 (3.07, 4.94) | 2.51 (2.32, 2.71) |
| NO_2_+PM_10_ |  |  |  |  |
| Low | Ref | 3.96 (3.56, 4.41) | 2.44 (1.88, 3.18) | 2.24 (2.11, 2.38) |
| Intermediate | 0.88 (0.82, 0.94) | 4.02 (3.61, 4.48) | 2.25 (1.70, 3.00) | 2.23 (2.09, 2.38) |
| High | 0.96 (0.89, 1.04) | 4.73 (4.24, 5.27) | 3.74 (2.95, 4.74) | 2.41 (2.23, 2.60) |
| NO_x_+PM_2.5_ |  |  |  |  |
| Low | Ref | 3.97 (3.57, 4.42) | 2.49 (1.92, 3.23) | 2.24 (2.10, 2.38) |
| Intermediate | 0.90 (0.84, 0.96) | 4.11 (3.69, 4.58) | 2.26 (1.70, 3.02) | 2.29 (2.14, 2.44) |
| High | 1.02 (0.94, 1.10) | 4.99 (4.47, 5.57) | 3.94 (3.10, 4.99) | 2.54 (2.35, 2.75) |
| NO_x_+PM_10_ |  |  |  |  |
| Low | Ref | 3.97 (3.57, 4.42) | 2.49 (1.92, 3.23) | 2.24 (2.10, 2.38) |
| Intermediate | 0.88 (0.82, 0.95) | 4.04 (3.63, 4.51) | 2.22 (1.67, 2.97) | 2.25 (2.11, 2.40) |
| High | 0.97 (0.90, 1.05) | 4.78 (4.29, 5.33) | 3.78 (2.98, 4.79) | 2.44 (2.26, 2.63) |
| **Liver cancer** |  |  |  |  |
| PM_2.5_+NO_2_ |  |  |  |  |
| Low | Ref | 6.47 (4.78, 8.74) | 2.64 (1.16, 6.00) | 1.81 (1.44, 2.28) |
| Intermediate | 1.24 (0.97, 1.59) | 4.69 (3.26, 6.75) | 1.02 (0.25, 4.14) | 1.62 (1.25, 2.12) |
| High | 1.25 (0.93, 1.68) | 4.48 (2.96, 6.77) | 2.46 (0.90, 6.75) | 1.68 (1.23, 2.30) |
| PM_2.5_+NO_x_ |  |  |  |  |
| Low | Ref | 6.46 (4.78, 8.74) | 2.64 (1.16, 6.00) | 1.81 (1.44, 2.28) |
| Intermediate | 1.24 (0.96, 1.59) | 4.68 (3.25, 6.73) | 1.02 (0.25, 4.13) | 1.62 (1.24, 2.11) |
| High | 1.24 (0.92, 1.67) | 4.47 (2.95, 6.76) | 2.45 (0.89, 6.73) | 1.67 (1.22, 2.29) |
| PM_10_+NO_2_ |  |  |  |  |
| Low | Ref | 6.84 (5.07, 9.23) | 2.72 (1.20, 6.17) | 1.81 (1.44, 2.29) |
| Intermediate | 1.32 (1.04, 1.69) | 4.55 (3.15, 6.58) | 1.04 (0.26, 4.20) | 1.72 (1.33, 2.22) |
| High | 1.37 (1.02, 1.83) | 5.04 (3.35, 7.58) | 2.77 (1.01, 7.58) | 1.88 (1.39, 2.55) |
| PM_10_+NO_x_ |  |  |  |  |
| Low | Ref | 6.84 (5.07, 9.23) | 2.72 (1.20, 6.17) | 1.81 (1.44, 2.29) |
| Intermediate | 1.32 (1.03, 1.68) | 4.54 (3.14, 6.56) | 1.03 (0.25, 4.19) | 1.71 (1.32, 2.22) |
| High | 1.37 (1.02, 1.83) | 5.03 (3.34, 7.56) | 2.76 (1.01, 7.57) | 1.88 (1.38, 2.55) |
| NO_2_+PM_2.5_ |  |  |  |  |
| Low | Ref | 5.11 (3.67, 7.12) | 2.64 (1.16, 6.00) | 1.70 (1.35, 2.15) |
| Intermediate | 1.14 (0.89, 1.46) | 5.72 (4.12, 7.95) | 0.94 (0.23, 3.81) | 1.61 (1.25, 2.07) |
| High | 1.12 (0.84, 1.51) | 4.14 (2.77, 6.18) | 2.22 (0.81, 6.09) | 1.52 (1.12, 2.07) |
| NO_2_+PM_10_ |  |  |  |  |
| Low | Ref | 5.11 (3.67, 7.12) | 2.64 (1.16, 5.99) | 1.70 (1.35, 2.15) |
| Intermediate | 1.12 (0.88, 1.43) | 5.64 (4.06, 7.82) | 0.92 (0.23, 3.75) | 1.58 (1.23, 2.04) |
| High | 1.07 (0.80, 1.43) | 3.94 (2.64, 5.87) | 2.11 (0.77, 5.79) | 1.45 (1.07, 1.97) |
| NO_x_+PM_2.5_ |  |  |  |  |
| Low | Ref | 5.08 (3.64, 7.09) | 3.14 (1.47, 6.73) | 1.74 (1.37, 2.20) |
| Intermediate | 1.17 (0.91, 1.49) | 5.92 (4.27, 8.22) | 0.48 (0.07, 3.41) | 1.64 (1.27, 2.11) |
| High | 1.15 (0.86, 1.55) | 4.22 (2.82, 6.31) | 2.26 (0.82, 6.21) | 1.53 (1.12, 2.09) |
| NO_x_+PM_10_ |  |  |  |  |
| Low | Ref | 5.08 (3.63, 7.09) | 3.13 (1.46, 6.72) | 1.74 (1.37, 2.20) |
| Intermediate | 1.15 (0.90, 1.47) | 5.83 (4.21, 8.08) | 0.47 (0.07, 3.35) | 1.61 (1.25, 2.08) |
| High | 1.09 (0.82, 1.47) | 4.01 (2.69, 5.98) | 2.15 (0.78, 5.90) | 1.46 (1.07, 1.98) |
| **CAD** |  |  |  |  |
| PM_2.5_+NO_2_ | Ref | 1.94 (1.82, 2.08) | 1.09 (0.92, 1.29) | 1.39 (1.34, 1.44) |
| Low | 0.90 (0.87, 0.94) | 2.25 (2.09, 2.42) | 1.38 (1.16, 1.65) | 1.32 (1.27, 1.38) |
| Intermediate | 0.83 (0.79, 0.87) | 2.00 (1.85, 2.17) | 1.26 (1.03, 1.53) | 1.29 (1.22, 1.35) |
| High |  |  |  |  |
| PM_2.5_+NO_x_ | Ref | 1.94 (1.82, 2.08) | 1.09 (0.92, 1.29) | 1.39 (1.34, 1.44) |
| Low | 0.90 (0.87, 0.94) | 2.25 (2.09, 2.41) | 1.38 (1.16, 1.65) | 1.32 (1.27, 1.38) |
| Intermediate | 0.83 (0.79, 0.87) | 2.00 (1.84, 2.17) | 1.25 (1.03, 1.52) | 1.28 (1.22, 1.35) |
| High |  |  |  |  |
| PM_10_+NO_2_ | Ref | 1.99 (1.86, 2.13) | 1.06 (0.89, 1.27) | 1.39 (1.34, 1.44) |
| Low | 0.99 (0.95, 1.03) | 2.27 (2.11, 2.44) | 1.48 (1.25, 1.76) | 1.45 (1.39, 1.50) |
| Intermediate | 0.91 (0.87, 0.96) | 2.30 (2.13, 2.49) | 1.40 (1.15, 1.70) | 1.41 (1.34, 1.48) |
| High |  |  |  |  |
| PM_10_+NO_x_ | Ref | 1.99 (1.86, 2.13) | 1.06 (0.89, 1.27) | 1.39 (1.34, 1.44) |
| Low | 0.99 (0.95, 1.03) | 2.27 (2.11, 2.43) | 1.48 (1.25, 1.76) | 1.45 (1.39, 1.50) |
| Intermediate | 0.91 (0.87, 0.95) | 2.30 (2.12, 2.48) | 1.40 (1.15, 1.70) | 1.41 (1.34, 1.48) |
| High |  |  |  |  |
| NO_2_+PM_2.5_ |  |  |  |  |
| Low | Ref | 1.98 (1.85, 2.13) | 1.22 (1.03, 1.44) | 1.41 (1.36, 1.46) |
| Intermediate | 0.92 (0.88, 0.95) | 2.14 (1.99, 2.30) | 1.25 (1.04, 1.50) | 1.33 (1.27, 1.38) |
| High | 0.95 (0.91, 1.00) | 2.32 (2.15, 2.50) | 1.39 (1.14, 1.68) | 1.44 (1.37, 1.51) |
| NO_2_+PM_10_ |  |  |  |  |
| Low | Ref | 1.98 (1.85, 2.13) | 1.21 (1.03, 1.43) | 1.41 (1.36, 1.46) |
| Intermediate | 0.90 (0.86, 0.93) | 2.09 (1.94, 2.25) | 1.22 (1.02, 1.46) | 1.29 (1.24, 1.35) |
| High | 0.90 (0.86, 0.95) | 2.20 (2.04, 2.38) | 1.32 (1.09, 1.60) | 1.37 (1.31, 1.44) |
| NO_x_+PM_2.5_ |  |  |  |  |
| Low | Ref | 1.99 (1.85, 2.13) | 1.23 (1.04, 1.45) | 1.41 (1.36, 1.46) |
| Intermediate | 0.91 (0.88, 0.95) | 2.14 (1.99, 2.30) | 1.20 (1.00, 1.45) | 1.33 (1.28, 1.38) |
| High | 0.95 (0.91, 1.00) | 2.33 (2.16, 2.51) | 1.43 (1.18, 1.72) | 1.44 (1.37, 1.51) |
| NO_x_+PM_10_ |  |  |  |  |
| Low | Ref | 1.98 (1.85, 2.13) | 1.23 (1.04, 1.45) | 1.41 (1.36, 1.46) |
| Intermediate | 0.89 (0.86, 0.93) | 2.08 (1.94, 2.24) | 1.17 (0.97, 1.41) | 1.30 (1.25, 1.35) |
| High | 0.91 (0.87, 0.95) | 2.21 (2.05, 2.39) | 1.35 (1.12, 1.64) | 1.37 (1.31, 1.44) |
| IS |  |  |  |  |
| PM_2.5_+NO_2_ |  |  |  |  |
| Low | Ref | 2.05 (1.77, 2.37) | 1.03 (0.68, 1.55) | 1.21 (1.12, 1.32) |
| Intermediate | 0.95 (0.87, 1.04) | 2.04 (1.75, 2.38) | 1.24 (0.84, 1.83) | 1.20 (1.10, 1.32) |
| High | 0.88 (0.79, 0.97) | 1.78 (1.50, 2.12) | 1.15 (0.75, 1.76) | 1.03 (0.92, 1.16) |
| PM_2.5_+NO_x_ |  |  |  |  |
| Low | Ref | 2.05 (1.77, 2.38) | 1.03 (0.68, 1.55) | 1.21 (1.12, 1.32) |
| Intermediate | 0.95 (0.87, 1.03) | 2.04 (1.75, 2.38) | 1.24 (0.84, 1.82) | 1.20 (1.10, 1.32) |
| High | 0.87 (0.79, 0.97) | 1.77 (1.49, 2.11) | 1.14 (0.75, 1.75) | 1.03 (0.92, 1.15) |
| PM_10_+NO_2_ |  |  |  |  |
| Low | Ref | 1.93 (1.66, 2.24) | 0.95 (0.63, 1.45) | 1.21 (1.12, 1.32) |
| Intermediate | 0.95 (0.88, 1.04) | 2.02 (1.73, 2.35) | 1.27 (0.87, 1.87) | 1.19 (1.09, 1.30) |
| High | 0.88 (0.79, 0.97) | 1.95 (1.65, 2.31) | 1.21 (0.79, 1.86) | 1.05 (0.94, 1.18) |
| PM_10_+NO_x_ |  |  |  |  |
| Low | Ref | 1.93 (1.66, 2.24) | 0.95 (0.63, 1.45) | 1.21 (1.12, 1.32) |
| Intermediate | 0.95 (0.88, 1.03) | 2.01 (1.73, 2.35) | 1.27 (0.87, 1.86) | 1.18 (1.08, 1.30) |
| High | 0.87 (0.79, 0.97) | 1.94 (1.64, 2.30) | 1.21 (0.79, 1.85) | 1.05 (0.94, 1.17) |
| NO_2_+PM_2.5_ |  |  |  |  |
| Low | Ref | 1.91 (1.63, 2.24) | 1.41 (0.98, 2.03) | 1.23 (1.13, 1.34) |
| Intermediate | 0.95 (0.87, 1.03) | 2.14 (1.84, 2.49) | 0.91 (0.58, 1.43) | 1.20 (1.10, 1.31) |
| High | 1.02 (0.92, 1.13) | 2.11 (1.79, 2.49) | 1.25 (0.82, 1.91) | 1.19 (1.07, 1.33) |
| NO_2_+PM_10_ |  |  |  |  |
| Low | Ref | 1.91 (1.63, 2.24) | 1.41 (0.98, 2.02) | 1.23 (1.13, 1.34) |
| Intermediate | 0.95 (0.87, 1.03) | 2.13 (1.83, 2.48) | 0.90 (0.57, 1.42) | 1.19 (1.09, 1.31) |
| High | 1.01 (0.91, 1.12) | 2.08 (1.77, 2.45) | 1.23 (0.80, 1.89) | 1.17 (1.05, 1.31) |
| NO_x_+PM_2.5_ |  |  |  |  |
| Low | Ref | 1.87 (1.59, 2.19) | 1.37 (0.94, 1.97) | 1.23 (1.13, 1.34) |
| Intermediate | 0.95 (0.87, 1.03) | 2.13 (1.83, 2.48) | 0.96 (0.61, 1.49) | 1.20 (1.10, 1.32) |
| High | 1.03 (0.93, 1.14) | 2.18 (1.85, 2.56) | 1.25 (0.82, 1.92) | 1.19 (1.07, 1.33) |
| NO_x_+PM_10_ |  |  |  |  |
| Low | Ref | 1.87 (1.59, 2.19) | 1.36 (0.94, 1.97) | 1.23 (1.13, 1.34) |
| Intermediate | 0.95 (0.87, 1.03) | 2.12 (1.82, 2.47) | 0.95 (0.61, 1.48) | 1.20 (1.09, 1.31) |
| High | 1.01 (0.91, 1.12) | 2.14 (1.82, 2.52) | 1.23 (0.81, 1.89) | 1.17 (1.05, 1.31) |
| PAD |  |  |  |  |
| PM_2.5_+NO_2_ |  |  |  |  |
| Low | Ref | 2.50 (2.19, 2.86) | 1.64 (1.20, 2.25) | 1.14 (1.05, 1.24) |
| Intermediate | 0.97 (0.88, 1.07) | 3.03 (2.62, 3.51) | 1.66 (1.15, 2.40) | 1.05 (0.95, 1.17) |
| High | 0.93 (0.83, 1.05) | 2.60 (2.18, 3.10) | 1.00 (0.60, 1.68) | 0.98 (0.86, 1.12) |
| PM_2.5_+NO_x_ |  |  |  |  |
| Low | Ref | 2.51 (2.19, 2.86) | 1.64 (1.20, 2.25) | 1.14 (1.05, 1.24) |
| Intermediate | 0.97 (0.88, 1.07) | 3.04 (2.62, 3.51) | 1.66 (1.16, 2.40) | 1.05 (0.95, 1.17) |
| High | 0.93 (0.82, 1.05) | 2.59 (2.17, 3.09) | 1.00 (0.60, 1.67) | 0.98 (0.86, 1.12) |
| PM_10_+NO_2_ |  |  |  |  |
| Low | Ref | 2.67 (2.33, 3.05) | 1.60 (1.15, 2.23) | 1.14 (1.04, 1.24) |
| Intermediate | 1.06 (0.97, 1.17) | 3.08 (2.66, 3.55) | 1.76 (1.23, 2.50) | 1.14 (1.03, 1.26) |
| High | 0.98 (0.88, 1.11) | 2.71 (2.28, 3.23) | 1.17 (0.71, 1.92) | 1.06 (0.94, 1.21) |
| PM_10_+NO_x_ |  |  |  |  |
| Low | Ref | 2.67 (2.33, 3.05) | 1.60 (1.15, 2.23) | 1.14 (1.04, 1.24) |
| Intermediate | 1.06 (0.97, 1.17) | 3.08 (2.66, 3.55) | 1.76 (1.24, 2.50) | 1.14 (1.03, 1.26) |
| High | 0.98 (0.87, 1.10) | 2.71 (2.27, 3.23) | 1.16 (0.71, 1.92) | 1.06 (0.93, 1.21) |
| NO_2_+PM_2.5_ |  |  |  |  |
| Low | Ref | 2.64 (2.29, 3.04) | 1.28 (0.88, 1.88) | 1.15 (1.05, 1.26) |
| Intermediate | 0.94 (0.85, 1.03) | 2.85 (2.46, 3.29) | 2.05 (1.49, 2.83) | 1.08 (0.97, 1.19) |
| High | 1.09 (0.97, 1.23) | 2.88 (2.44, 3.40) | 1.22 (0.77, 1.92) | 1.08 (0.95, 1.22) |
| NO_2_+PM_10_ |  |  |  |  |
| Low | Ref | 2.64 (2.29, 3.04) | 1.28 (0.87, 1.88) | 1.15 (1.05, 1.26) |
| Intermediate | 0.92 (0.84, 1.01) | 2.79 (2.42, 3.23) | 2.01 (1.46, 2.78) | 1.06 (0.96, 1.17) |
| High | 1.06 (0.94, 1.19) | 2.79 (2.36, 3.28) | 1.17 (0.74, 1.86) | 1.04 (0.92, 1.18) |
| NO_x_+PM_2.5_ |  |  |  |  |
| Low | Ref | 2.63 (2.29, 3.03) | 1.28 (0.87, 1.88) | 1.15 (1.05, 1.25) |
| Intermediate | 0.93 (0.84, 1.02) | 2.85 (2.46, 3.30) | 2.10 (1.53, 2.89) | 1.07 (0.97, 1.19) |
| High | 1.10 (0.98, 1.23) | 2.87 (2.43, 3.39) | 1.15 (0.72, 1.85) | 1.08 (0.95, 1.23) |
| NO_x_+PM_10_ |  |  |  |  |
| Low | Ref | 2.63 (2.28, 3.03) | 1.28 (0.87, 1.87) | 1.14 (1.04, 1.25) |
| Intermediate | 0.91 (0.83, 1.00) | 2.80 (2.42, 3.24) | 2.06 (1.50, 2.84) | 1.05 (0.95, 1.17) |
| High | 1.06 (0.95, 1.19) | 2.78 (2.35, 3.27) | 1.11 (0.69, 1.78) | 1.05 (0.92, 1.19) |
| CKD |  |  |  |  |
| PM_2.5_+NO_2_ |  |  |  |  |
| Low | Ref | 3.52 (3.26, 3.81) | 1.13 (0.86, 1.49) | 1.57 (1.49, 1.65) |
| Intermediate | 1.10 (1.04, 1.16) | 4.22 (3.87, 4.59) | 1.55 (1.17, 2.06) | 1.66 (1.57, 1.76) |
| High | 1.12 (1.05, 1.20) | 3.97 (3.61, 4.38) | 1.08 (0.74, 1.59) | 1.79 (1.67, 1.92) |
| PM_2.5_+NO_x_ |  |  |  |  |
| Low | Ref | 3.53 (3.26, 3.81) | 1.13 (0.86, 1.49) | 1.57 (1.49, 1.65) |
| Intermediate | 1.10 (1.04, 1.16) | 4.21 (3.87, 4.58) | 1.55 (1.16, 2.05) | 1.66 (1.56, 1.76) |
| High | 1.11 (1.03, 1.18) | 3.92 (3.55, 4.32) | 1.07 (0.73, 1.57) | 1.76 (1.64, 1.89) |
| PM_10_+NO_2_ |  |  |  |  |
| Low | Ref | 3.58 (3.31, 3.88) | 1.06 (0.79, 1.43) | 1.53 (1.46, 1.62) |
| Intermediate | 1.11 (1.06, 1.18) | 4.27 (3.93, 4.63) | 1.55 (1.19, 2.03) | 1.77 (1.67, 1.87) |
| High | 1.20 (1.12, 1.28) | 4.15 (3.77, 4.56) | 1.24 (0.86, 1.79) | 1.86 (1.74, 2.00) |
| PM_10_+NO_x_ |  |  |  |  |
| Low | Ref | 3.58 (3.31, 3.88) | 1.06 (0.79, 1.43) | 1.53 (1.46, 1.62) |
| Intermediate | 1.11 (1.05, 1.17) | 4.26 (3.92, 4.62) | 1.55 (1.19, 2.03) | 1.77 (1.67, 1.87) |
| High | 1.18 (1.11, 1.27) | 4.09 (3.72, 4.51) | 1.22 (0.85, 1.77) | 1.84 (1.72, 1.97) |
| NO_2_+PM_2.5_ |  |  |  |  |
| Low | Ref | 3.56 (3.29, 3.86) | 1.15 (0.88, 1.51) | 1.56 (1.49, 1.65) |
| Intermediate | 0.98 (0.93, 1.04) | 3.48 (3.20, 3.78) | 1.14 (0.83, 1.55) | 1.52 (1.43, 1.60) |
| High | 0.93 (0.87, 1.00) | 3.57 (3.25, 3.91) | 1.18 (0.84, 1.65) | 1.47 (1.37, 1.57) |
| NO_2_+PM_10_ |  |  |  |  |
| Low | Ref | 3.56 (3.28, 3.86) | 1.14 (0.87, 1.50) | 1.56 (1.49, 1.65) |
| Intermediate | 0.97 (0.92, 1.03) | 3.45 (3.17, 3.75) | 1.12 (0.82, 1.53) | 1.50 (1.42, 1.59) |
| High | 0.90 (0.84, 0.96) | 3.44 (3.14, 3.77) | 1.14 (0.81, 1.60) | 1.42 (1.32, 1.52) |
| NO_x_+PM_2.5_ |  |  |  |  |
| Low | Ref | 3.57 (3.30, 3.87) | 1.12 (0.85, 1.48) | 1.56 (1.48, 1.64) |
| Intermediate | 0.97 (0.92, 1.02) | 3.45 (3.17, 3.76) | 1.17 (0.86, 1.59) | 1.51 (1.43, 1.60) |
| High | 0.96 (0.89, 1.02) | 3.60 (3.29, 3.95) | 1.18 (0.84, 1.66) | 1.49 (1.39, 1.60) |
| NO_x_+PM_10_ |  |  |  |  |
| Low | Ref | 3.57 (3.29, 3.87) | 1.11 (0.85, 1.47) | 1.55 (1.48, 1.64) |
| Intermediate | 0.96 (0.91, 1.01) | 3.42 (3.14, 3.72) | 1.16 (0.85, 1.57) | 1.49 (1.41, 1.58) |
| High | 0.92 (0.86, 0.99) | 3.48 (3.17, 3.81) | 1.14 (0.81, 1.60) | 1.44 (1.35, 1.54) |
| All-cause mortality |  |  |  |  |
| PM_2.5_+NO_2_ |  |  |  |  |
| Low | Ref | 1.90 (1.79, 2.01) | 1.43 (1.24, 1.64) | 1.15 (1.11, 1.19) |
| Intermediate | 0.93 (0.90, 0.97) | 1.66 (1.54, 1.79) | 1.23 (1.03, 1.48) | 1.04 (1.00, 1.09) |
| High | 0.92 (0.87, 0.96) | 1.57 (1.44, 1.72) | 1.51 (1.26, 1.82) | 1.02 (0.96, 1.07) |
| PM_2.5_+NO_x_ |  |  |  |  |
| Low | Ref | 1.90 (1.79, 2.01) | 1.43 (1.24, 1.64) | 1.15 (1.11, 1.19) |
| Intermediate | 0.93 (0.90, 0.97) | 1.66 (1.54, 1.79) | 1.23 (1.03, 1.48) | 1.05 (1.00, 1.09) |
| High | 0.91 (0.87, 0.96) | 1.57 (1.44, 1.71) | 1.51 (1.26, 1.81) | 1.01 (0.96, 1.07) |
| PM_10_+NO_2_ |  |  |  |  |
| Low | Ref | 1.91 (1.80, 2.03) | 1.38 (1.19, 1.60) | 1.13 (1.09, 1.17) |
| Intermediate | 0.96 (0.93, 1.00) | 1.65 (1.54, 1.78) | 1.26 (1.06, 1.50) | 1.11 (1.06, 1.15) |
| High | 0.99 (0.95, 1.04) | 1.79 (1.64, 1.94) | 1.76 (1.48, 2.10) | 1.10 (1.05, 1.16) |
| PM_10_+NO_x_ |  |  |  |  |
| Low | Ref | 1.91 (1.80, 2.03) | 1.38 (1.19, 1.60) | 1.13 (1.09, 1.17) |
| Intermediate | 0.97 (0.93, 1.00) | 1.65 (1.54, 1.78) | 1.26 (1.06, 1.50) | 1.11 (1.06, 1.15) |
| High | 0.99 (0.95, 1.04) | 1.78 (1.63, 1.94) | 1.76 (1.47, 2.10) | 1.10 (1.04, 1.16) |
| NO_2_+PM_2.5_ |  |  |  |  |
| Low | Ref | 1.89 (1.77, 2.01) | 1.34 (1.15, 1.56) | 1.16 (1.11, 1.20) |
| Intermediate | 0.94 (0.91, 0.98) | 1.73 (1.61, 1.86) | 1.52 (1.29, 1.78) | 1.06 (1.02, 1.11) |
| High | 1.03 (0.98, 1.08) | 1.76 (1.62, 1.91) | 1.48 (1.23, 1.79) | 1.13 (1.08, 1.19) |
| NO_2_+PM_10_ |  |  |  |  |
| Low | Ref | 1.88 (1.77, 2.01) | 1.34 (1.15, 1.56) | 1.15 (1.11, 1.20) |
| Intermediate | 0.93 (0.90, 0.96) | 1.71 (1.59, 1.83) | 1.49 (1.27, 1.75) | 1.04 (1.00, 1.09) |
| High | 0.98 (0.94, 1.03) | 1.68 (1.55, 1.82) | 1.41 (1.17, 1.70) | 1.08 (1.03, 1.14) |
| NO_x_+PM_2.5_ |  |  |  |  |
| Low | Ref | 1.88 (1.77, 2.01) | 1.37 (1.18, 1.59) | 1.16 (1.12, 1.20) |
| Intermediate | 0.94 (0.91, 0.98) | 1.73 (1.61, 1.86) | 1.49 (1.27, 1.75) | 1.06 (1.01, 1.10) |
| High | 1.04 (0.99, 1.09) | 1.77 (1.63, 1.92) | 1.48 (1.22, 1.78) | 1.14 (1.08, 1.20) |
| NO_x_+PM_10_ |  |  |  |  |
| Low | Ref | 1.88 (1.77, 2.01) | 1.37 (1.18, 1.59) | 1.16 (1.12, 1.20) |
| Intermediate | 0.93 (0.89, 0.96) | 1.70 (1.58, 1.83) | 1.47 (1.25, 1.72) | 1.04 (1.00, 1.08) |
| High | 0.99 (0.94, 1.04) | 1.69 (1.55, 1.83) | 1.41 (1.17, 1.70) | 1.09 (1.03, 1.14) |

The hazard ratios and 95% confidence intervals were calculated using the time-varying Cox proportional hazard regression models adjusted for age, sex, ethnicity, alcohol consumption, smoking status, diet score, physical activity, annual household income, education, and Townsend Deprivation Index.

**Abbreviation:** MAFLD, metabolic-associated fatty liver disease; PM_2.5_, fine particulate matter with diameter <2.5 μm; PM_10_, particulate matter with diameter <10 μm; NO_2_, nitrogen dioxide; NO_x_, nitrogen oxides; CAD, coronary artery disease; IS, ischemic stroke; PAD, peripheral artery disease; CKD, chronic kidney disease.


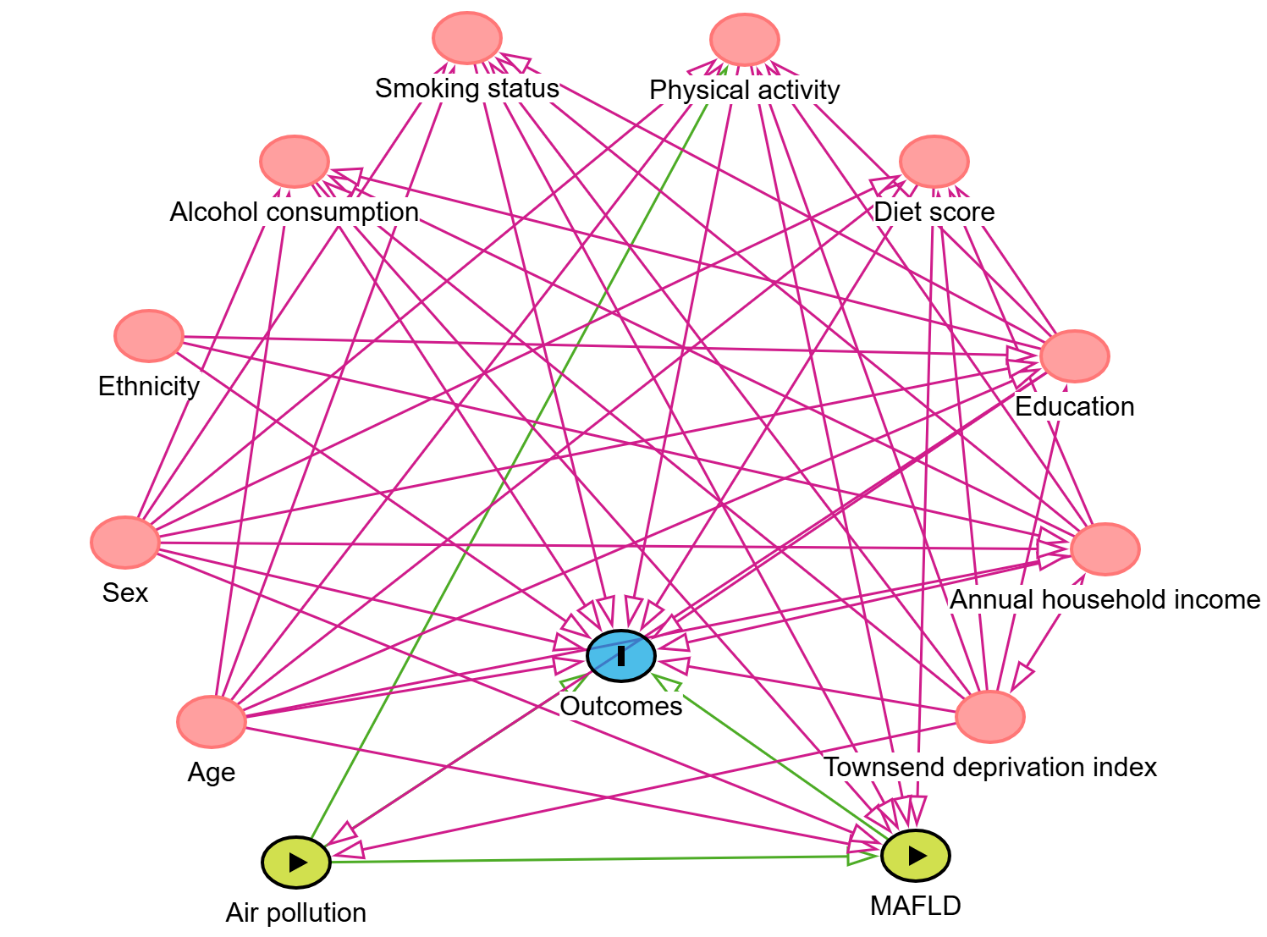


**Supplemental Figure 1. Directed acyclic graph of exposure to ambient air pollution and MAFLD with health outcomes.**


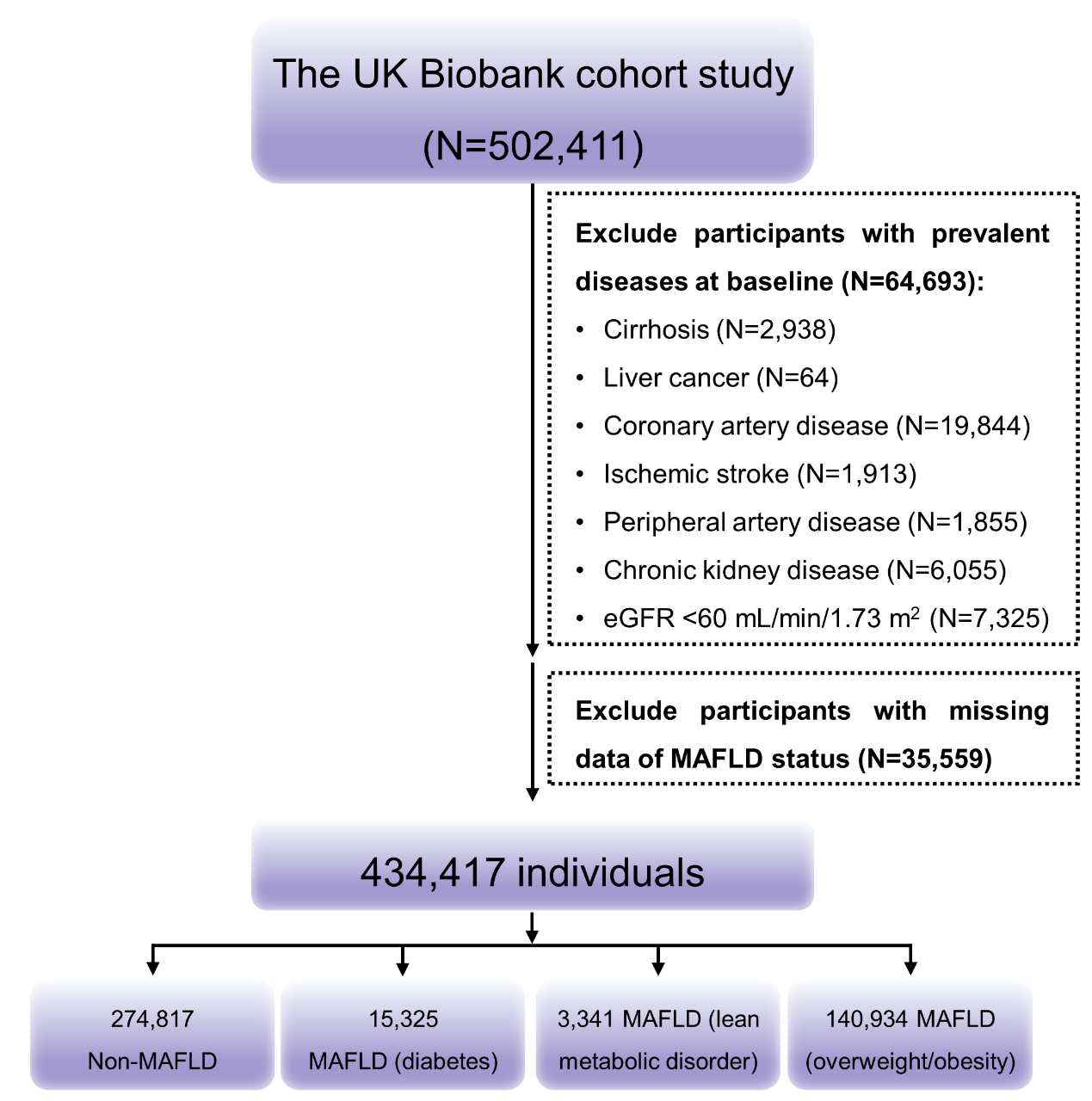


**Supplemental Figure 2. Study population flow chart.**


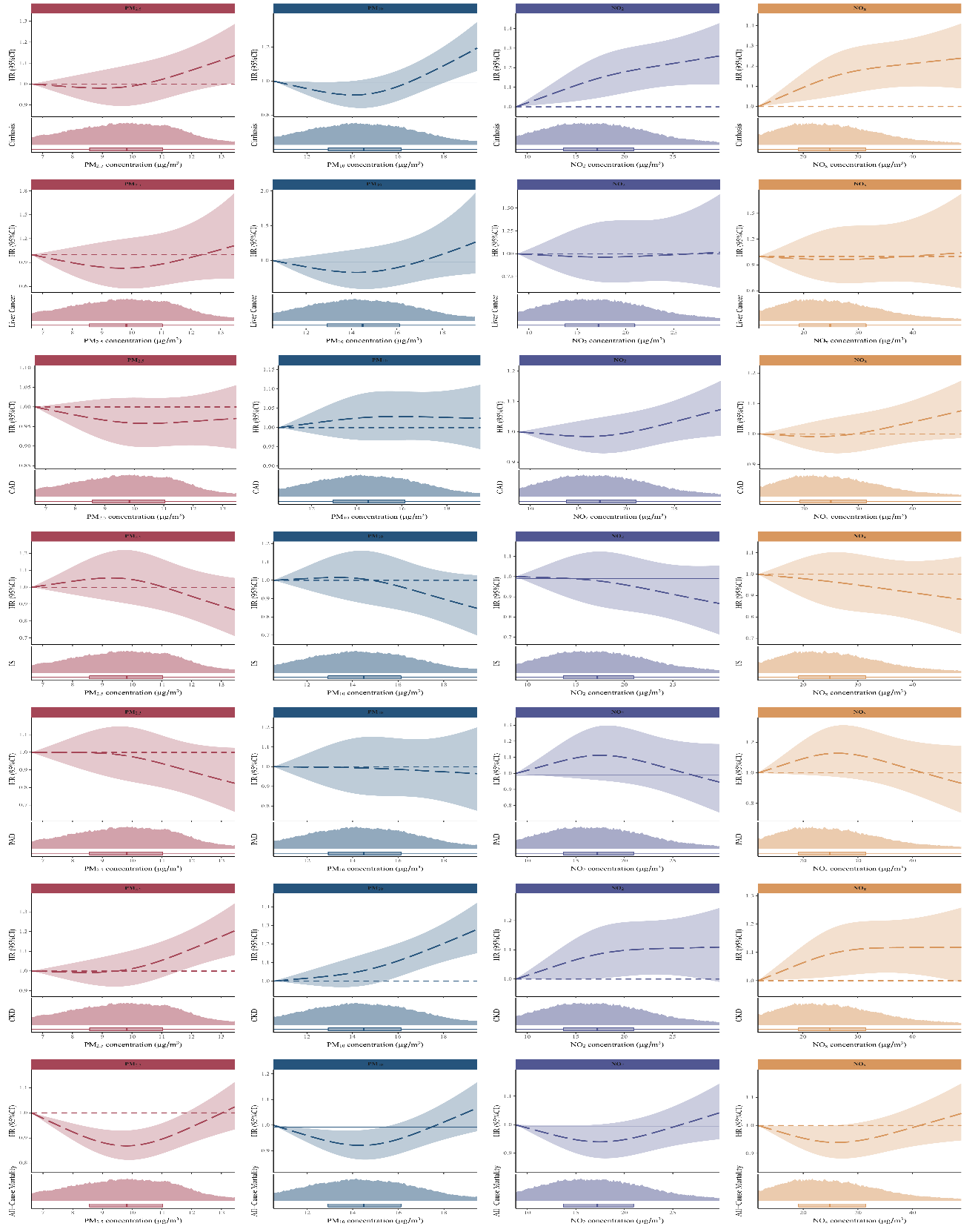
**Supplemental Figure 3. Concentration-response curves for long-term exposure to air pollutants and risk of** **intrahepatic and extrahepatic morbidity and all-cause mortality.** A restricted cubic spline regression model was used to estimate the dose-response relationship. Hazard ratios (dashed lines) and 95% confidence intervals (shaded areas) were adjusted for age, sex, ethnicity, alcohol consumption, smoking status, diet score, physical activity, annual household income, education, and Townsend Deprivation Index.
